# DNA/MVA Vaccination of HIV-1 Infected Participants with Viral Suppression on Antiretroviral Therapy, followed by Treatment Interruption: Elicitation of Immune Responses without Control of Re-Emergent Virus

**DOI:** 10.1371/journal.pone.0163164

**Published:** 2016-10-06

**Authors:** Melanie Thompson, Sonya L. Heath, Bentley Sweeton, Kathy Williams, Pamela Cunningham, Brandon F. Keele, Sharon Sen, Brent E. Palmer, Nicolas Chomont, Yongxian Xu, Rahul Basu, Michael S. Hellerstein, Suefen Kwa, Harriet L. Robinson

**Affiliations:** 1 AIDS Research Consortium of Atlanta, Atlanta, Georgia, United States of America; 2 Department of Medicine, University of Alabama at Birmingham, Birmingham, Alabama, United States of America; 3 Alabama Vaccine Research Clinic, University of Alabama at Birmingham, Birmingham, Alabama, United States of America; 4 AIDS and Cancer Virus Program, Leidos Biomedical Research, Inc., Frederick National Laboratory for Cancer Research, Frederick, Maryland, United States of America; 5 University of Colorado Anschutz Medical Campus, Aurora, Colorado, United States of America; 6 Centre de recherche du CHUM and Department of Microbiology, Infectiology and Immunology, Université de Montréal, Montreal, Canada; 7 Hope Clinic of the Emory Vaccine Center, Division of Infectious Diseases, Department of Medicine, School of Medicine, Emory University, Atlanta, Georgia, United States of America; 8 GeoVax, Inc., Atlanta, Georgia, United States of America; Azienda Ospedaliera Universitaria di Perugia, ITALY

## Abstract

GV-TH-01, a Phase 1 open-label trial of a DNA prime—Modified Vaccinia Ankara (MVA) boost vaccine (GOVX-B11), was undertaken in HIV infected participants on antiretroviral treatment (ART) to evaluate safety and vaccine-elicited T cell responses, and explore the ability of elicited CD8+ T cells to control viral rebound during analytical treatment interruption (TI). Nine men who began antiretroviral therapy (ART) within 18 months of seroconversion and had sustained plasma HIV-1 RNA <50 copies/mL for at least 6 months were enrolled. Median age was 38 years, median pre-ART HIV-1 RNA was 140,000 copies/ml and mean baseline CD4 count was 755/μl. Two DNA, followed by 2 MVA, inoculations were given 8 weeks apart. Eight subjects completed all vaccinations and TI. Clinical and laboratory adverse events were generally mild, with no serious or grade 4 events. Only reactogenicity events were considered related to study drug. No treatment emergent viral resistance was seen. The vaccinations did not reduce viral reservoirs and virus re-emerged in all participants during TI, with a median time to re-emergence of 4 weeks. Eight of 9 participants had CD8+ T cells that could be stimulated by vaccine-matched Gag peptides prior to vaccination. Vaccinations boosted these responses as well as eliciting previously undetected CD8+ responses. Elicited T cells did not display signs of exhaustion. During TI, temporal patterns of viral re-emergence and Gag-specific CD8+ T cell expansion suggested that vaccine-specific CD8+ T cells had been stimulated by re-emergent virus in only 2 of 8 participants. In these 2, transient decreases in viremia were associated with Gag selection in known CD8+ T cell epitopes. We hypothesize that escape mutations, already archived in the viral reservoir, plus a poor ability of CD8+ T cells to traffic to and control virus at sites of re-emergence, limited the therapeutic efficacy of the DNA/MVA vaccine.

**Trial Registration:**

clinicaltrials.gov NCT01378156

## Introduction

Over the past two decades, increasingly potent and tolerable antiretroviral therapy (ART) has transformed the death sentence of AIDS into a long-term chronic infection with potential for a normal lifespan[1]. Decreased mortality due to ART and the ongoing high rate of new infections, approximately 2 million in 2014 alone, have resulted in steadily increasing numbers of people living with HIV. Of an estimated 37 million persons living with HIV at the end of 2014, only about 15.8 million were on ART as of mid-2015[2, 3]. Immediate access to ART at the time of HIV diagnosis, regardless of CD4 count, recommended by some since 2012, is now recommended worldwide based on data from the START study [4–7]. These global recommendations will more than double the number of people considered to be in need of ART. Delivering lifelong uninterrupted access to ART is a daunting challenge globally, requiring not only funding for drugs, but delivery systems, adequate medical workforce, and accessible care infrastructures. While more tolerable than ever before, ART is not without toxicity and the need for ongoing safety monitoring increases the global burden on both funding and infrastructure. Likewise, interruptions in access to therapy or inadequate medication adherence frequently lead to drug resistance. For successful viral suppression, patients must be linked to care, retained in care, offered ART, and they must successfully adhere to ART. In the absence of a cure, we are increasingly challenged to explore novel means of maintaining viral suppression. A safe and effective therapeutic vaccine could decrease, delay, or eliminate our reliance on long-term ART by stimulating an immune response capable of virologic control. The current study was undertaken to evaluate the safety and immunogenic potential of the GOVX-B11 vaccine in HIV-infected adults on successful ART and during an analytic treatment interruption, and to explore its potential for limiting viral rebound upon discontinuation of therapy.

GOVX-B11 is a vaccine that uses DNA priming and MVA boosting to elicit both T cell and antibody (Ab) responses. Both DNA and MVA components express clade B Gag, Pol and Env and produce immunogenic virus-like particles. In a Phase 2a prevention trial conducted by the HIV Vaccine Trials Network (HVTN 205), GOVX-B11 elicited Env-specific Ab in 93%, CD4+ T cells in 66% and CD8+ T cells in 22% of participants [8]. In HIV-infected persons with pre-existing CD8+ T cell responses, it was hypothesized that the GOVX-B11 vaccination could expand pre-existing CD8+ T cell responses due to the ability of the MVA component to boost previously primed CD8+ T cells [9].

The primary objective of the study was to evaluate the safety of the therapeutic use of GOVX-B11 during vaccination, analytic treatment interruption and treatment reinstitution. Secondary objectives were to evaluate the immunogenicity of GOVX-B11 during the vaccination phase of the trial and to evaluate HIV-1 RNA levels, CD4+ T cell counts, and HIV-1-specific immune responses during the analytic treatment interruption phase of the trial.

## Materials and Methods

### Clinical Trial

#### Human Subjects Protection

**Fig 1:**
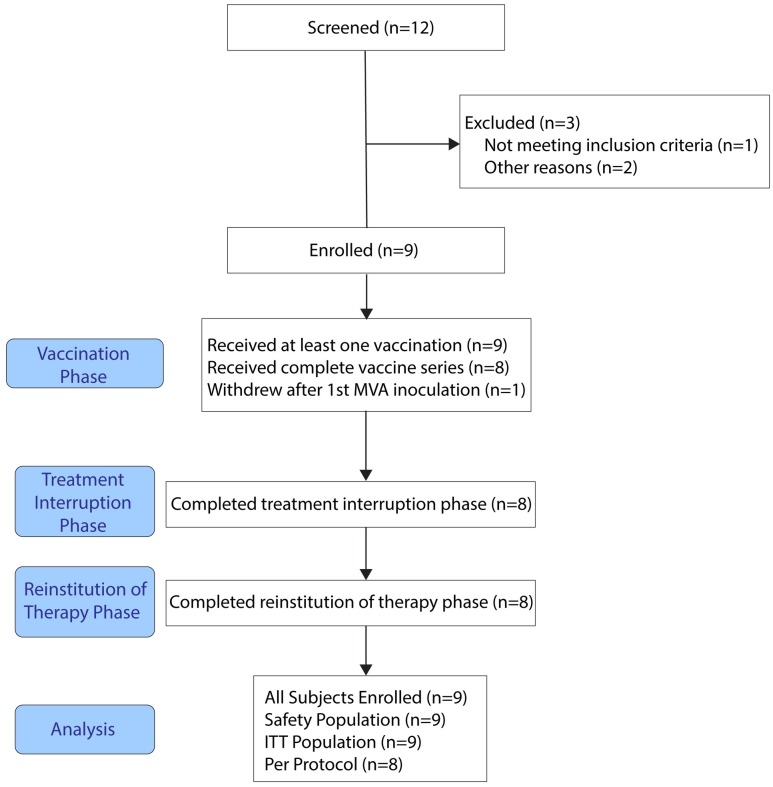
Patient Disposition (CONSORT) flow chart. ITT, intent to treat.

The study was conducted at the AIDS Research Consortium of Atlanta (ARCA), the University of Alabama School of Medicine (Birmingham; UAB), and the AIDS Research Alliance (ARA), Los Angeles with the approval and oversight of duly-constituted Institutional Review Boards at each site. Written informed consent was obtained from each participant before any study procedures were conducted. The study was conducted in accordance with the Declaration of Helsinki and Transparent Reporting of Evaluation with Nonrandomized Designs (TREND) (S1 File) and is registered at clinical trials.gov as trial number NCT01378156. Participant recruitment and follow up was from June 2010 until May 2014. The consort diagram for the study is presented in (Fig 1).

#### Study Design

**Fig 2:**
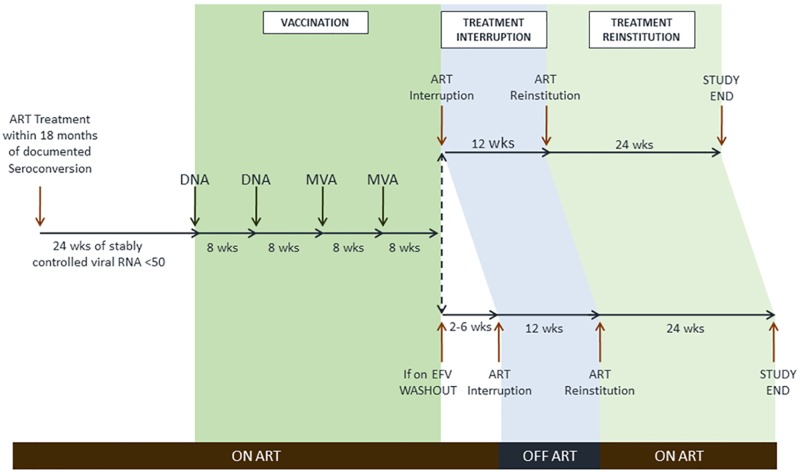
Trial Schema. The phases of the trial are indicated for screening, vaccination, treatment interruption and treatment reinstitution. An efavirenz washout period occurred for those on an efavirenz-containing regimen. ART, antiretroviral treatment; EFV, efavirenz.

The three phases of this open label study, vaccination, treatment interruption, and treatment reinstitution, are shown in Fig 2 (S2 File, Protocol). Written informed consent was obtained at a screening visit within 21 days of the baseline visit. The vaccination phase began at the baseline visit. Two DNA prime inoculations (3 mg each) were given eight weeks apart, followed by two MVA inoculations (1x10^8^ tissue culture infectious doses (TCID)_50_), also eight weeks apart. All inoculations were intra-muscular in the upper arm using a needle and syringe. A 12-week treatment interruption was instituted at 8 weeks post the 2^nd^ MVA inoculation in participants not on efavirenz. Because of the heightened risk of emergent viral resistance due to the prolonged half-life of efavirenz, participants on efavirenz were changed to a boosted protease inhibitor or integrase inhibitor regimen prior to treatment interruption until efavirenz levels were undetectable. Thresholds for immediate reinstitution of therapy during treatment interruption included CD4 count below 350/μL (two consecutive measurements at least 2 weeks apart) or HIV-1 RNA >300,000 c/mL (two consecutive measurements at least 2 weeks apart). Following treatment interruption, participants restarted ART and were followed for 24 weeks. Participants with viral suppression, or low level viremia, who declined to reinstitute therapy at the designated time were followed until therapy was reinstituted. Throughout the trial participants were regularly monitored for safety, CD4 count, HIV-1 RNA, and the emergence of drug-resistant virus. Specimens were obtained for intensive immunologic assays (described below) at baseline, one to two weeks after each immunization, at treatment interruption, 2 weeks after the re-emergence of virus, at treatment reinstitution, and at 3 and 6 months after ART reinstitution. Specimens were banked at each visit for viral resistance testing in the event of virologic failure while on therapy, or viral rebound when off therapy. Virologic failure or rebound was defined as HIV-1 RNA above 200 copies/mL, at two time points at least 2 weeks apart.

#### Study Participants

Self-selected eligible participants were 18–45 years old, had begun ART within 18 months of the most recent negative HIV antibody or documented acute HIV infection and had been on stable ART for at least 6 months at the time of screening. Participants had documented pretreatment CD4 and HIV-1 RNA levels, no history of virologic failure (defined as HIV-1 RNA above 50 copies/mL (RT-PCR) or 75 copies/mL (bDNA)), screening HIV-1 RNA below 50 copies/mL, screening CD4 T cell count of at least 500 cells/μL, and CD4 nadir above 350 cells/μL. Those with hepatitis B or C, prior cardiovascular disease, or a Framingham Global Risk Assessment score consistent with high short-term risk for cardiac disease were excluded. Initially, the protocol required pre-treatment CD4 and HIV-1 RNA to be drawn at least 8 weeks following diagnosis of acute infection, to better allow for estimation of viral set point. During the course of the trial, this entry requirement was eliminated in order to accept participants who had begun ART prior to 8 weeks after diagnosis of acute infection.

#### Study Procedures

Screening procedures after administration of informed consent included physical examination with vital signs; 12-lead electrocardiogram (at screen and baseline); recording of medical and vaccination history and concomitant medications; and laboratory monitoring consisting of complete blood count with differential, serum chemistries (including electrolytes, AST, ALT, bilirubin, alkaline phosphatase, calcium, and creatine phosphokinase), fasting lipid profile, hepatitis B and C serology, serum pregnancy test for females of childbearing potential; urinalysis; CD4 cell count; and HIV-1 RNA. Cardiovascular risk was assessed using Framingham Global Risk Assessment score, due to the increased risk of cardiovascular events in persons who discontinue antiretroviral therapy [10]. Baseline and subsequent study visit evaluations included symptom-driven physical examination with vital signs; hematology and chemistry, urinalysis; urine pregnancy test for females; CD4 cell count; HIV-1 RNA; stored samples for immunology and virology at specified visits; and assessment of concomitant medications and adverse events. Reactogenicity was assessed at the time of each vaccination and three days afterward. Adverse events were coded according to the Medical Dictionary for Regulatory Activities (MedDRA), and graded for intensity according to the National Institute of Allergy and Infectious Diseases Division of AIDS “Table for Grading the Severity of Adult and Pediatric Adverse Events.” Severity and relationship to study drug were assessed by the study investigator. Plasma HIV-1 RNA was quantified using The Roche COBAS^®^ TaqMan^®^ HIV-1RNA assay version 2.0 (lower limit of quantification of 20 copies/mL), and CD4 cell counts were quantified using flow cytometry at UAB’s Flow Cytometry Lab and at Quest Laboratories (ARCA and ARA). Efavirenz levels were quantified by HPLC through Advanced Diagnostic Laboratories, National Jewish Health, Denver, CO. Viral resistance was evaluated using the Monogram Biosciences PhenoSense GT assay.

#### Vaccine Product

The GOVX-B11 vaccine consists of a Clade B recombinant DNA prime (pGA2/JS7) [11, 12] and a Clade B recombinant MVA boost (MVA62B)[13]. Both the prime and boost express Gag-Pol and Env and produce non-infectious virus-like particles displaying trimeric membrane bound Env. The DNA expresses a full length Env (gp160), whereas the MVA expresses an Env that is truncated for 115 C-terminal amino acids of the endodomain of gp41 to enhance cell surface expression and stability during manufacture[14]. The Gag sequences in the vaccine are from HIV-HXB-2 and BH10; and, the Env sequences from the CCR-5-tropic HIV-ADA[12].

### Assays

#### Intracellular cytokine staining

Assays for intracellular cytokine staining (ICS) used pools of vaccine-matched 15-mer peptides overlapping by 11 amino acids (21st Century Biochemicals). Peptides were dissolved in dimethyl sulfoxide (DMSO; Sigma) and pooled by the major HIV antigens: Gag (group-specific antigen, 1 pool), Env (envelope, 2 pools), and Pol (polymerase, 1 pool), and diluted to a final individual concentration of 1 μg/mL. To map Gag responses, 11 consecutive pools were prepared with 11 peptides each. For assays, frozen PBMCs were thawed and rested overnight in complete media at 37°C in a 5% CO2 atmosphere and then tested using standard conditions. Cells were first stained with Live Dead Fixable Green Dead Cell Stain Kit (Life Technologies) at room temperature for 30 minutes. Next, samples were fixed and permeabilized with Cytofix/Cytoperm (BD) at 4°C for 20 minutes. Finally, samples were stained with CD3 APC-CY7 (BD, clone SP34-2), CD4 PE-Cy7 (BD, clone L200), CD8 PerCP (BD, clone SK7), IFN-γ APC (BD, clone B27), and IL-2 PE (R&D, clone 5334). Results were collected using a FACSCanto flow cytometer and the software FACSDiva. Analysis was performed using FlowJo software. Cytokine responses were scored as a percent of total CD4+ or CD8+ T cells. Responses were considered enhanced if they were at least 2-times higher than the same response scored at baseline, at least two times the no-stimulation value and > 0.02% of total CD4+ or CD8+ T cells.

#### Immunofluorescent staining of inhibitory receptors

For staining of inhibitory receptors, stimulated PBMCs were surface stained with anti-CD4 (V500; BD Biosciences), anti-CD3 (APC-H7; BD Biosciences), anti-CD8 (Alexa Fluor 405; Invitrogen), anti-PD-1 (FITC; BD Bioscience) and anti-human TIM-3 antibody (PE; R&D Systems) for 30 min at 4°C. Cells were washed, fixed, permeabilized (Invitrogen), and intracellularly stained with anti-IFN-γ (PE-Cy7; BD Biosciences), anti-IL-2 (BV605; BD Biosciences), and anti-CTLA-4 (APC; BD Biosciences) for 30 min at 4°C, washed and fixed with 1% formaldehyde. Cells were analyzed using a LSR-II flow cytometer (BD Immunocytometry Systems). One million events were collected. Electronic compensation was performed with Ab capture beads (BD Biosciences) stained separately with individual monoclonal Abs used in the test samples. The data files were analyzed using Diva (BD) and FlowJo Software (Treestar, Co). The expression of TIM-3, PD-1 and CTLA-4 was examined on cytokine-producing cells with frequencies ≥0.03% above the background (media control tube) to ensure an adequate number of events for analysis as previously validated by our laboratory [15–18]. Flourescence minus one (FMO) controls were used to set the gates for determining the mean fluorescent intensity (MFI) of TIM-3, PD-1 and CTLA-4 on Gag-responsive T cells expressing IFN-γ or IL-2.

#### Binding antibody

Assays for binding antibody used standard ELISA procedures with HEK293-produced HIV-1Bal gp120 and E. Coli-produced gp41 (both obtained from the NIH AIDS Reagent Program) at 1 μg/mL (50ng per well) for coating Nunc Maxisorp 96-well plates (Thermo Scientific) and goat anti-human IgG (Fc) horseradish peroxidase (Accurate Chemical & Scientific Corporation) and SureBlue TMB Microwell Peroxidase 1-component Substrate (KPL) for detection. All assays included a standard curve for human IgG (Sigma), and sera from an HIV-positive patient served as a reference standard. ELISA plates were read using a Vmax kinetic plate microreader (Molecular Devices) and concentrations of gp120 and gp41-specific antibody estimated by interpolation. Antibody responses were considered positive if they were at least 1.5-fold above pre-vaccination responses.

#### Measure of the HIV Reservoir

The size of the HIV reservoir was assessed by measuring the frequency of CD4+ T cells harboring integrated HIV DNA, as previously described [19]. In addition, the recently developed *Tat/Rev* Induced Limiting Dilution assay (TILDA) was used to measure the frequency of CD4+ T cells producing multiply-spliced (tat/rev) HIV RNA upon stimulation with PMA/ionomycin [20].

#### Viral Sequencing

In subjects 01–4 and 01–8, viral RNA from blood plasma was sequenced from samples obtained immediately following viral rebound and just prior to treatment reinstitution. The HIV *gag* gene was amplified and sequenced using a limiting-dilution PCR known as single genome amplification (SGA) followed by direct Sanger sequencing as previously described [21]. All PCR positive amplicons were directly sequenced using BigDye Terminator chemistry (Applied Biosystems). Any sequence with evidence of double peaks was excluded from further analysis. All 95 *gag* sequences were deposited to GenBank under accession numbers KU140953-KU141047.

#### Statistical Analysis

Descriptive analysis was performed on all safety parameters, CD4 count, and HIV-1 RNA levels. Medians (25^th^, 75^th^ quartiles) are reported for levels of HIV-1 RNA and elicited T and B cell responses. Means and standard deviations are reported for absolute CD4 and CD8 T cells counts. Mann-Whitney test was used for analysis of T cell exhaustion markers.

## Results

### Subjects

#### Disposition

Nine participants met the entry criteria and were enrolled in the study. Eight participants completed the entire study. One participant withdrew consent after the 3^rd^ vaccine administration (Fig 1).

#### Baseline Characteristics

Table 1 summarizes the baseline characteristics of the 9 participants. All were male, 2 were African American and one was Hispanic/Latino, and mean age was 38 years. Prior to initiation of ART, the median HIV-1 RNA level was 140,000 copies per mL (range 421–15,300,000). Two participants began ART before seroconversion. One of these (01–6) began ART in the same week as diagnosis of acute HIV infection, based upon an HIV-1 RNA level of 15,300,000 copies/mL and a negative HIV antibody. The mean pre-ART CD4 count was 755 per μl in 8 participants and was missing in one participant. The median time from HIV seroconversion to initiation of ART was 7 weeks with a range of 2 weeks to 45 weeks. The median time between initiation of ART and first vaccination was 62 weeks with a range of 31 to 373 weeks. At baseline, all subjects had controlled HIV-1 RNA below 50 copies/ml for a minimum of 6 months.

**Table 1:** Demographics and History of HIV Seroconversion and Antiretroviral Therapy.

Subject ID^1^	Age	Viral Load at ART Initiation, Copies/ML	Weeks from HIV Diagnosis to ART Initiation	Weeks from ART Initiation to First Vaccination	CD4 Count at First Vaccination, Cells/μL (Percent)	ART at Baseline^6^
01–1	45	140,000	10	50	1612 (42%)	MVC, TDF/FTC
01–2^2^	24	421	45	38	674 (N/A^5^)	EFV/TDF/FTC
01–3^3^	37	132,510	30	62	501 (39%)	EFV/TDF/FTC
01–4	47	1,575	0.5^4^	373	918 (46%)	ATV /rtv + ABC/LAM
01–5	38	159,830	4	54	708 (35%)	EFV/TDF/FTC
01–6	36	15,300,000	0	198	734 (N/A^5^)	RAL + TDF/FTC
01–7	45	181,979	7	81	535 (41%)	EFV/TDF/FTC
01–8^2^	23	6,423	20	31	534 (36%)	EFV/TDF/FTC
01–9	42	780,000	2	70	585 (43%)	EFV/TDF/FTC

^1^All participants were male;

^2^Black or African American;

^3^Hispanic/Latino;

^4^Participant started on ART approximately 2 weeks before seroconversion;

^5^N/A, not available;

^6^Abbreviations: ABC = abacavir; ATV = atazanavir; EFV = efavirenz; FTC = emtricitabine; LAM = lamivudine; MVC = maraviroc; RAL = raltegravir; rtv = ritonavir (low dose); TDF = tenofovir disoproxil fumerate.

#### Safety

Most clinical adverse events were mild, transient, and deemed not to be related to study drug, except for those associated with vaccination reactogenicity. There were 121 clinical adverse events including 106 mild 15 moderate, and no severe events, no serious adverse events occurred, and no clinical adverse events resulted in study product discontinuation. The most common clinical adverse events included headache (5 mild and 2 moderate), upper respiratory tract infection, myalgia, oropharyngeal pain, and nasal congestion (6 each); cough (5), diarrhea and back pain (4 each); lymphadenopathy, vomiting, fatigue and hematoma at venipuncture site (3 each), and contusion, sciatica, arthralgia, insomnia, productive cough, pulmonary congestion, and night sweats (2 each). Mild clinical adverse events with 2 or more occurrences and all moderate events are listed in S1 Table.

There were 28 vaccine reactogenicity adverse events (S2 Table) of which 24 were mild and 4 were moderate. None were severe or serious. The most common event was injection site pain (17 cases, 15 mild, two moderate, one of which lasted eight days). Other events included fatigue (one mild, one moderate); two instances of generalized pruritis (mild), and one instance each of asthenia (moderate), diarrhea (mild), dysgeusia (mild), throat irritation (mild), and injection site erythema, edema, or warmth (all mild).

Laboratory adverse events included 102 Grade 1 events, 12 Grade 2 events and 8 grade 3 events. (S3 Table). There were no Grade 4 adverse events and no events resulted in study drug discontinuation. Two Grade 3 and three Grade 1 CPK elevations occurred in three patients. All were transient and considered by the investigators to be associated with exercise. A single, transient Grade 3 decrease in albumin occurred. Five Grade 3 and eight Grade 2 bilirubin elevations occurred in four patients, all of whom were taking atazanavir as ongoing therapy or during efavirenz washout. Other than bilirubin, transient Grade 2 changes in potassium, blood glucose, calcium and total cholesterol occurred once in 4 patients. The most common Grade 1 abnormalities, included changes in total bilirubin (26), carbon dioxide (22), blood glucose (14). Transient Grade 1 elevations of AST (12) and ALT (10) occurred. No laboratory adverse events were considered related to study product.

Six participants underwent efavirenz washout lasting from 2 to 14 weeks. The participant with the longest duration of efavirenz washout was an African-American male with low body mass who had reported substantial efavirenz side effects upon instituting initial therapy. His efavirenz level did not become undetectable until week 38.

### Virologic Response

During the Vaccination Phase, 6 of 9 participants experienced 13 episodes of transient low-level viremia (<200 copies/mL) that spontaneously re-suppressed. These episodes of low level viremia occurred on the day of DNA (twice) or MVA inoculation (once) and at one week following DNA inoculation (5 episodes in 4 patients) or MVA inoculation (one episode). One patient (01–3) experienced 3 episodes of transient low-level viremia at other times during the Vaccination Phase. HIV-1 RNA was below 50 copies/mL for all participants at the end of the Vaccination Phase.

**Fig 3:**
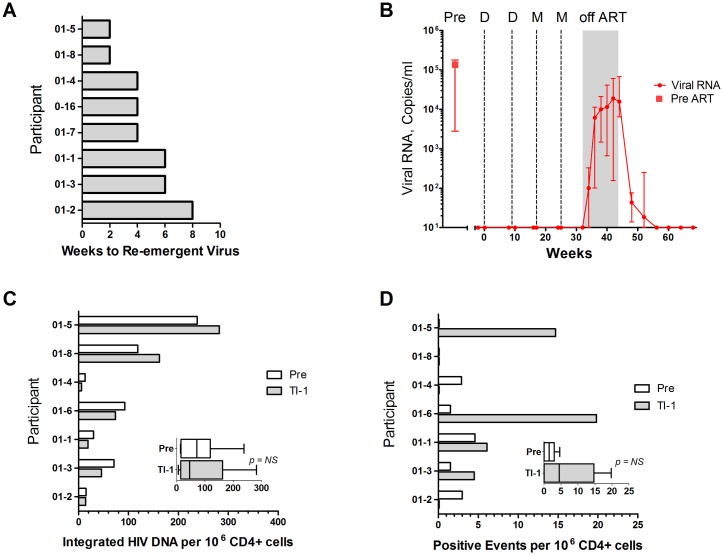
Timing and Height of Re-emergent Virus and Viral Reservoirs. Panel A. presents weeks to re-emergent virus and panel B presents temporal levels of viral RNA throughout the study. Temporal levels of viral RNA are given as medians and interquartile ranges. In panel B, the median pre-ART level of viral RNA is indicated with a square symbol. Panel C shows the frequency of circulating CD4+ T cells harboring integrated HIV DNA. Panel D shows the number of CD4+ cells with inducible Tat/ Rev mRNA as measured by TILDA. Insets in C and D give the medians and interquartile ranges for all participants. Participants are listed in the same order in A, C, and D. Participant 01–7 is not included in reservoir analyses because pre-vaccination cells were not available. Pre designates the day of vaccination and TI-1, the day of treatment interruption. Samples with HIV-1 RNA <50 copies/mL are plotted at 10 copies per ml.

During the 12-week Treatment Interruption Phase, virus re-emerged (>200 copies/mL) in all participants (Fig 3). The median time to rebound was 4 weeks (range from 2–8 weeks) (Fig 3A). Median HIV-1 RNA at Week 4 after TI was 6,100 copies/ml and peak median HIV-1 RNA was 18,974 copies/ml at Week 10 after TI (Fig 3B). At Week 12 after TI, median HIV-1 RNA was 15,686 copies/ml. One participant (01–1) decided to delay reinstitution of treatment for 26 weeks because viremia remained low (636 copies/mL at 12 weeks following TI). This patient reinstituted therapy at 56 weeks, when HIV-1 RNA was 6328 copies/mL.

Vaccination did not measurably affect the size of the viral reservoirs in CD4+ T cells isolated from PBMCs. Levels of proviral DNA ranged from 13 to 238 copies per million CD4+ T cells at baseline; and, from 6 to 282 copies per million CD4+ T cells at the time of TI (Fig 3C). The frequency of CD4+ T cells with inducible HIV was measured using PMA and ionomycin stimulation followed by limiting dilution analyses for expression of Tat and Rev mRNAs. These assays scored from 0 to 19.8 positive events per million CD4+ T cells. The frequency of events did not show a trend with vaccination. Participants with the least time to viral re-emergence tended to have the highest levels of integrated DNA (Fig 3A and 3C). Patterns of inducible RNA varied among the participants and were generally low, as expected in these participants who started ART within 18 months following seroconversion. [22]

### Immunologic Response

#### CD4+ and CD8+ T cell responses

**Fig 4:**
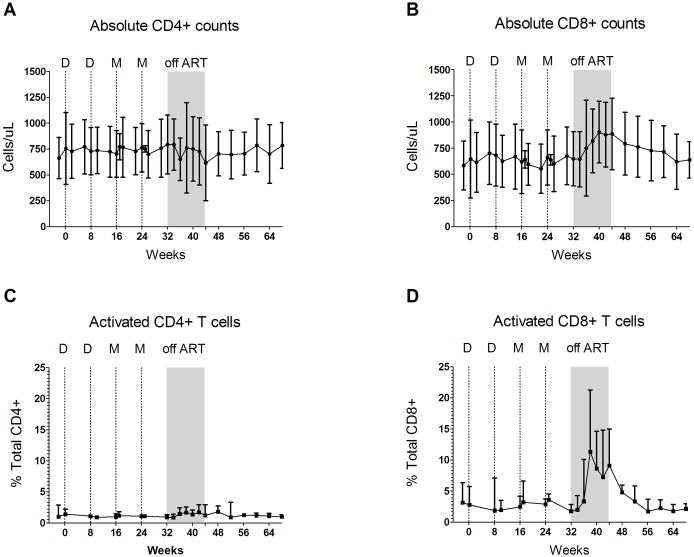
Temporal Levels of Absolute CD4+ and CD8+ T Cells Throughout the Study. All data are means with the error bars indicating standard deviations. Panels A and B present absolute CD4+ and CD8+ T cell counts as cells/μl. Panels C and D present activated cells as a percent of total CD4+ and CD8+ T cells respectively. Cells displaying CD38+ and HLA-DR surface markers are considered activated T cells.

Temporal mean CD4+ count was 756 and 793 cells/μL at baseline and pre-TI, respectively, declined to 616 cells/μL at the week of reinstitution and returned to 799 cells/μL at the end of treatment reinstitution, (Fig 4A). In contrast, mean absolute CD8+ T cells increased from 645 and 647 cells/μL at baseline and pre TI, respectively, to 885 cells/μL at the end of TI (Fig 4B). On the last study visit, 24 weeks following treatment reinstitution, mean CD8 count had decreased to 621 cells/μL, comparable to baseline levels.

During TI, pronounced changes were seen in the display of the CD38 and HLA-DR markers of activation on CD8+ T cells [23] (Fig 4D). Mean activated CD8+ T cells remained near the baseline level of 3.4% of total CD8+ T cells until TI, spiked as virus re-emerged to peak at 6 weeks post TI at 13.2% of total CD8+ T cells, fell to 10.4% of total CD8+ T cells by the end of TI and returned to baseline levels within 12 weeks of treatment reinstitution. Testing of total CD4+ T cells for CD38 and HLA-DR expression did not reveal activation during treatment interruption. Activated cells as a percent of total CD4+ T cells averaged 1.6% of total CD4+ T cells over the different phases of the trial (Fig 4C).

Before vaccination, all 9 participants had CD8+ T cells that produced IFN-γ in response to stimulation with vaccine-matched peptides. These responses ranged from 0.09% to 0.83% of total CD8+ T cells. Of the 9 participants, 8 had pre-vaccination CD8+ T cells responding to Gag peptides, 3 to Env peptides, and 3 to Pol peptides. Pre-vaccination, responding CD4+ T cells were detected in 5 participants (0.09 to 0.26% of total CD4+ T cells). Four participants had CD4+ T cells responding to Gag, 2 to Env and 1 to Pol. Baseline levels of antibody to the gp120 and gp41 subunits of Env ranged from estimated values of 0.02 to 5.2 μg/ml for gp120 and 0.3 to 12 μg/ml for gp41.

**Fig 5:**
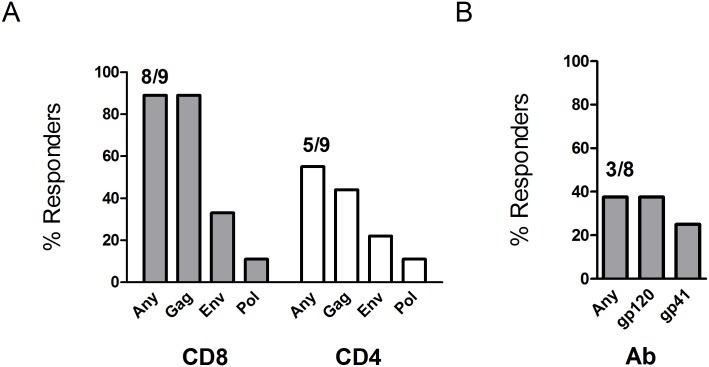
Response rates. Response rates are for (A) vaccine enhanced CD8+ and CD4+ T cells scored by ICS and (B) vaccine enhanced Ab to gp120 and gp41 scored by ELISA.

Vaccinations enhanced both T cell and antibody responses to the vaccine. The most frequently enhanced response to vaccination was for IFNγ producing CD8+ T cells, which were stimulated to at least 2-fold higher levels than observed at baseline in 8/9 participants for Gag, 3/9 participants for Env, and 1/9 participants for Pol (Fig 5). The highest level of responding IFNγ CD8+ T cells was 2.4% of total CD8+ T cells (participant 01–6). IFNγ producing CD4+ T cells were enhanced in 5/9 participants (4 for Gag, 2 for Env and 1 for Pol). The highest induced IFNγ CD4+ Gag response was 0.18% of total CD4+ T cells (participant 01–4). Antibody responses were enhanced in 3 participants for gp120 and 2 for gp41 (Fig 5B). The participants with enhanced antibody responses had the lowest levels of baseline Env-specific antibody.

**Fig 6:**
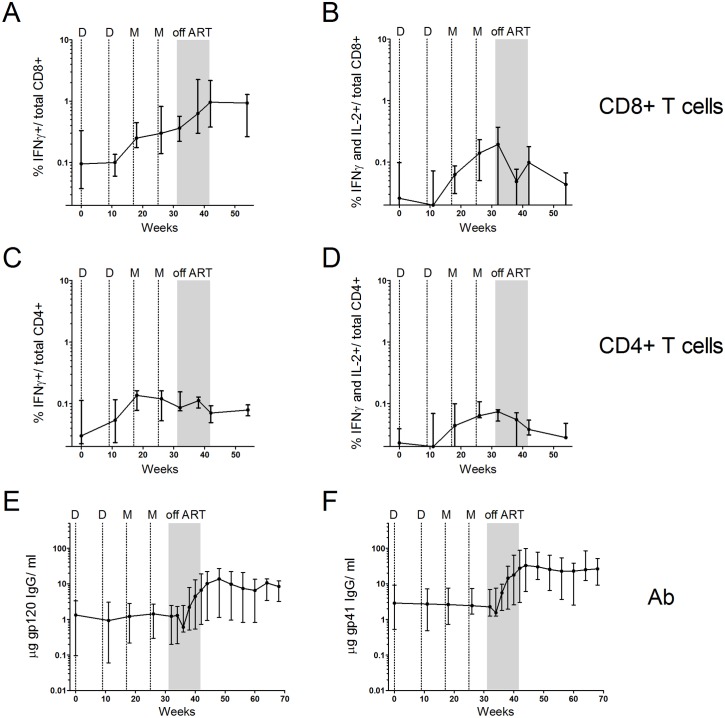
Temporal magnitudes of vaccine stimulated T cell and Ab responses. Panels A-D: medians with interquartile ranges for Gag-stimulated T cell responses scored using ICS. IFNγ (panel A) and IFNγ + IL-2 co-producing (Panel B) CD8+ cells as % of total CD8+ T cells. IFNγ (Panel C) and IFNγ + IL-2 (Panel D) co-producing CD4+ cells as % of total CD4+ T cells. Panels E and F: gp120 and gp41 Ab (μg/ml). Data are for the vaccination, treatment interruption (indicated in grey) and treatment reinstitution phases of the trial. Dotted lines indicate the timing of DNA (D) and MVA (M) immunizations. All 9 participants are included in T cell data through the 1^st^ MVA inoculation after which data are for the 8 participants that completed the trial. The Ab data are for the 8 participants that completed the trial.

The median frequencies of CD8+ T cells producing IFNγ in response to Gag peptides rose during the vaccination and TI phases of the trial, whereas the frequencies of IFNγ and IL-2 co-producing cells (stimulated in a subset of participants) rose during the vaccination phase, but declined during the TI phase of the trial (Fig 6A and 6B). The median IFNγ Gag response was 0.095% of total CD8+ T cells at baseline, 0.3% after the last MVA boost, and 0.96% post-TI. Median levels of IFNγ and IL-2 co-producing cells were 0.03% at baseline, 0.14% following the last MVA boost and 0.1% at the end of TI.

In contrast to the CD8+ T cell response, the median frequencies of IFNγ producing Gag-specific CD4+ T cell responses, rose during the vaccination phase, but did not expand during TI (Fig 6C). IFNγ and Il-2 co-producing CD4+ T cells responses, which were about 50% of the total CD4+ T cell response also rose during the vaccination phase of the trial, but declined during TI (Fig 6D). Env-specific Ab responses showed modest increases in 3 of 8 participants during the vaccination phase and increased in all 8 participants during TI (Fig 6E and 6F). At the end of TI, the median gp120-specific Ab response was 5-times higher, and the median gp41 response 11-times higher, than after the last MVA.

**Fig 7:**
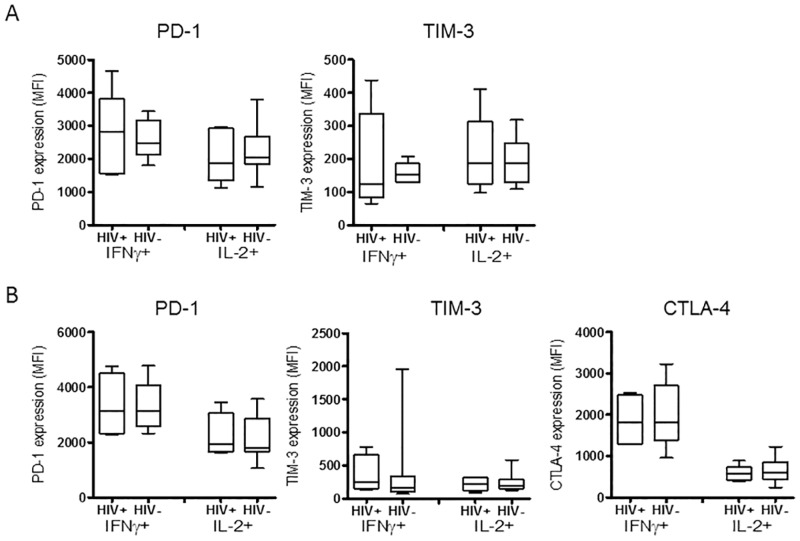
Inhibitory receptor expression on Gag-specific IFNγ and IL-2 producing CD8+ and CD4+ T cells. Panel A shows the mean fluorescent intensity (MFI) for PD1 and TIM-3 on Gag-specific CD8+ T cells. Panel B shows MFI for PD-1, CTLA-4 and TIM-3 on Gag-specific CD4+ T cells. HIV+ designates virologically suppressed, ART treated subjects undergoing therapeutic vaccination whereas HIV- designates uninfected individuals undergoing prophylactic vaccination in the HVTN 205 trial. Inhibitory receptor expression was determined for IFNγ and IL-2 responding cells, indicated below the X axis. Box plots show median values and interquartile ranges; the whiskers indicate the lowest and highest points within 1.5 interquartile ranges of the lower and higher quartiles. No significant difference in inhibitory receptor expression was found between HIV+ and HIV- subjects using Mann-Whitney T test.

Because T cells responding to a chronic infection can become exhausted [24], exhibiting a phenotype manifested by the expression of high levels of inhibitory receptors, the levels of inhibitory receptors on responding CD4+ and CD8+ T cells were compared to those elicited by GOVX-B11 immunizations in uninfected people. This was done to allow comparison of the levels of inhibitory receptors in the infected, ART-treated and then vaccinated population, with the levels in vaccinated uninfected people, who were not at risk for having exhausted T cells. Using flow cytometry, surface expression of programmed death 1 (PD-1), cytotoxic T lymphocyte-associated protein 4 (CTLA-4) and T cell immunoglobulin mucin-3 (TIM-3) were measured on Gag-responding T cells expressing IFNγ or IL-2. Cells from two weeks after the last MVA immunization were used for this analysis (Fig 7). The expression of inhibitory receptors PD-1 or TIM-3 on Gag-stimulated CD8+ T cells (Fig 7A) or of PD-1, CTLA-4 or TIM-3 on Gag-stimulated CD4+ T cells (Fig 7B) were similar in vaccinated HIV infected and HIV uninfected subjects, suggesting that exhaustion was not present.

#### Temporal association of Gag-specific CD8+ T cells and re-emergent virus

The temporal association of Gag-specific CD8+ T cell responses and re-emergent virus was examined in the 8 individuals who underwent TI. Participants were ordered starting with the individual (01–1) who delayed re-institution, followed by the remaining individuals (01–2 to 01–8), ordered according to the level of viral rebound during TI measured by the area under the curve (Table 2).

**Table 2:** HIV-1 RNA, Area Under the Curve (AUC) During TI.

Participant	HIV-1 RNA, AUC
01–1	22,147
01–2	1,131
01–3	53,599
01–4	55,992
01–5	149,248
01–6	434,770
01–7	533,914
01–8	701,984

**Fig 8:**
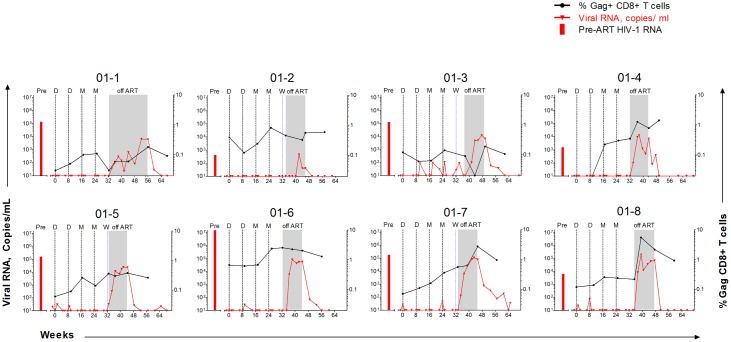
Temporal viral RNA and responding T Cells. Panels show data for the 8 participants in the trial who completed the study ordered from the individual who delayed treatment re-institution (01–1) and then participants with increasing levels of viral RNA during the TI phase (Table 2, measured by area under the curve). The red bar at the left side of each panel indicates pre-ART levels of viral RNA. Red lines indicate temporal levels of viral RNA and black lines, temporal levels of Gag-specific IFNγ expressing CD8+ T cells. DNA and MVA immunizations are indicated by dashed black vertical lines designated D and M respectively. The initiation of an efavirenz washout is indicated by a dashed vertical blue line, W. Levels of HIV-1 RNA < 50 copies/mL are plotted as 20 copies/ml. In 01–3, a low CD8 ICS response at TI3 reflected high background at this time point.

Temporal patterns of re-emergent virus and Gag-specific CD8+ T cells differed among participants (Fig 8). In 01–4 and 01–8, Gag-specific CD8+ T cell responses expanded and contracted concomitant with re-emergent virus in plasma. In 01–4, re-emergent virus declined from 13,099 to 507 copies per ml; and, in 01–8, from 196,532 to 12,520 copies per ml of plasma. Subsequent increase in viremia was not associated with increased CD8+ T cell frequency in 01–8, but was associated with a late increase in 01–4.

To evaluate whether anti-Gag CD8+ T cells led to viral escape, the emerging virus from 01–4 and 01–8 was sequenced at rebound (2 weeks after TI for 01–8 and 4 weeks after TI for 01–4) and at treatment reinstitution. In 01–4, sequence variations were found in a known epitope for the A*02–02 haplotype of this participant (Table 3 and S1 Fig). Very limited mutations (4 out of 10,500 sequenced amino acids) occurred outside of this region suggesting viral escape by selection for mutations that occurred within the CD8 restricted epitope. The low number of mutations found in the virus from this participant was consistent with the individual having initiated ART before seroconversion.

**Table 3:** Sequence changes consistent with CD8+ T cell selection of re-emergent virus in 01–4 and 01–8.

Participant	HLA haplotype	Known epitope position	Source of Virus	Sequence
**01–4**	A*02–02	**368–376**	**TI-3**	**23/23 SQVTNTATI**
			**R-1**	**10/21 ---------**
				**10/21 ----S----**
				**01/21 -----A---**
**01–8**	A*24–02	**28–36**	**TI-2**	**15/22 RYRLKHIVW**
				**07/22 K-Q------**
			**R-1**	**07/29 ---------**
				**02/29 K-Q------**
				**20/29 ------L--**
**01–8**	A*02–01	**49–58**	**TI-2**	**21/22 GLLETSDGC**
				**01/22 ------E--**
			**R-1**	**07/29 ---------**
				**22/29 ------E--**
**01–8**	**Human**	**218–226**	**TI-2**	**15/22 AQAGPIAPG**
				**07/22 -----V---**
			**R-1**	**27/29 -----V---**
				**02/29 -----M---**
**01–8**	A*02–01	**267–274**	**TI-2**	15/22 ILGLNKIV
				**07/22 -M------**
			**R-1**	**01/29 --------**
				**19/29 -M------**
				**09/29 VM-------**
**01–8**	A*02–01	**362–370**	**TI-2**	**21/22 ILAEAMSQV**
				**01/22 V--------**
			**R-1**	**12/29 ----------**
				**17/29 V--------**

Rebound virus was sequenced post TI and again at ART reinstitution. Sequence changes are shown that occurred in known T cell epitopes for the participants’ HLA haplotypes. The epitopes are from the Los Alamos database, and positions are indicated relative to the genome of HIV-1-HXB2. The numbers before sequences indicate the number of sequences with the indicated change over the total number of executed sequences. Dashes indicate sequences identical to the most frequent sequence in the rebound virus. Examples for potential selection are limited to reinstitution virus with >50% mutant sequences from the most frequent TI sequence. Changes in sequences between TI and ART reinstitution included changes in abundance of specific sequences (not highlighted) as well as totally new sequences (highlighted in yellow). Complete sequences can be found in S1 and S2 Figs. TI-3 is rebound virus at four weeks post TI and TI2, rebound virus at two weeks post TI.

In contrast to participant 01–4; participant 01–8 had mutations in five known CD8 restricted epitopes that differentiated his virus at reinstitution from his re-emergent virus (Table 3 and S2 Fig). The higher level of mutations in 01–8 than in 01–4 was consistent with ART not being initiated in 01–8 until 20 weeks post seroconversion (Table 1). Similar to 01–4, virus at reinstitution from 01–8 had few sequence variations that occurred outside of the clusters of mutations in known CD8 restricted epitopes.

A very different pattern for re-emergent virus and Gag-specific CD8+ T cells was seen in 01–5 and 01–6. In these participants, re-emergent virus underwent very modest declines while Gag-specific CD8+ T cells remained essentially at their pre TI levels. Yet, another pattern of responses was seen in 01–1 and 01–7, where Gag-specific CD8+ T cells expanded over the time course of re-emergent virus, but did not have a close temporal relationship with the expansion (01–7) or the expansion and contraction of re-emergent virus (01–1). In 01–7, re-emergent virus remained unsuppressed while CD8+ T cells increased over the period of TI. In 01–1, who delayed re-institution, viremia decreased from 303 to 22 copies/mL, rebounded, declined from 636 to 82 copies/mL and rebounded again. While these expansions and contractions of virus were taking place, Gag-specific CD8+ T cells slowly increased but did not show evidence for concomitant expansion with the appearance of rebound virus.

#### Mapping of Gag response

**Fig 9:**
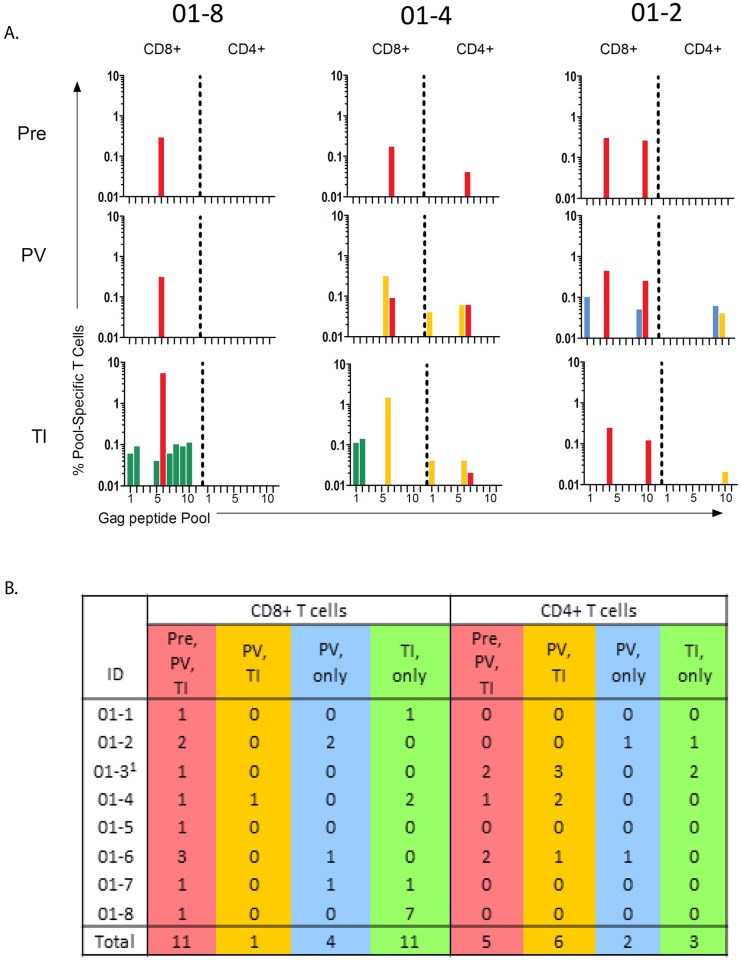
Mapping of Elicited T Cell Responses to 11 Pools of Sequential Gag Peptides. Panel A shows examples of the CD8+ and CD4+ responses in three participants. Panel B summarizes responses for the eight participants that completed the trial. ^1^01–3 had a high background at TI allowing only dominant responses to be scored. Pre, responses present before vaccination; PV, responses scored after the 2^nd^ MVA inoculation; TI, Responses scored at 2 weeks post the re-emergence of virus during treatment interruption. Red indicates responses present at baseline, post vaccination and either present or absent post TI, yellow indicates responses observed post vaccination and post treatment interruption, blue indicates responses scored only post vaccination and green indicates responses scored only during treatment interruption.

Vaccine-elicited T cell responses were further assessed by mapping the T cell response to 11 sequential Gag peptide pools. This was done at the time of the first DNA vaccination, after the last MVA vaccination and 2 weeks after the first detection of re-emergent virus. Four patterns of responses were identified (Fig 9). The first response pattern was primed by the infection (present at baseline), boosted by the vaccine and either stimulated or not stimulated by the re-emergent virus. The second pattern was detectable only post-vaccination and stimulated by the re-emergent infection. A third pattern was detectable only post-vaccination but not stimulated by the re-emergent infection, and the final pattern was detectable only post viral re-emergence. The first two patterns reached frequencies greater than 0.1% of CD8+ cells, but the latter two patterns were generally subdominant, and lower than 0.1% of total CD8+ cells.

Different participants had different patterns of Gag CD8+ T cell responses. In Fig 9, patient 01–8 demonstrates a single dominant CD8+ response (pool 6) at baseline, post vaccination and post TI (red). However, an additional 7 subdominant responses (green) appeared during TI. In participant 01–4, a response to pool 6 is present pre and post vaccination and a response to pool 5 appears post vaccination. The response to pool 5, but not to pool 6, expanded during TI. Two subdominant responses to pools 1 and 2 appeared during TI. Yet another pattern of responses is seen in participant 01–2. In this participant, pools 4 and 10 stimulated responses at baseline, post vaccination and post TI. However, after vaccination, responses to pools 1 and 9 appear. Neither of these responses was stimulated by the re-emergent virus. Gag-CD4+ T cell responses showed the same categories of responses, but with responses being lower and less frequent than the CD8+ T cell responses (Fig 9).

Fig 9B summarizes the mapping patterns for peptide pools for each participant and totals the responses for each pattern (see total). The most frequent patterns for CD8+ T cell stimulations were the relatively dominant responses present at baseline, post vaccination and post TI (11 responses) and the subdominant responses, first stimulated at TI (11 responses). Only one response was first seen post vaccination and then again post TI. In contrast 4 responses were first detected post vaccination, which were not stimulated by the re-emergent virus.

#### CD4/CD8 ratios in individual participants

To better assess the effect of the TI on the immune systems of the participants, the ratio of CD4+ to CD8+ T cells was computed for 7 of 8 participants at baseline, pre-TI, ART reinstitution, and at 24 weeks after reinstitution (Table 4). Data were not available for one participant. This analysis revealed that all but one participant (01–5) initiated the trial with a CD4/CD8 ratio greater than 1. These ratios were stable until the time of TI. During TI, the ratios dropped in all participants with the 3 participants with the highest HIV-1 RNA during TI (01–6, 01–7 and 01–8) dropping below 1 by the time ART was restarted. The ratio in participant 01–5, which was below 1 at the start, underwent a modest decline and recovered to pre-trial levels by 6 months after the re-initiation of ART. By the end of the trial, the CD4/CD8 ratios in all participants except for 01–8 (the individual with the highest HIV-1 RNA during TI), had returned to above 1 and the ratio in 01–8 was approaching 1 (value of 0.94).

**Table 4:** Ratio of Absolute CD4+ to Absolute CD8+ T cell Counts^1^.

ID	Day of 1st inoculation	Start of TI	Reinstitution of ART	Reinstitution Week 24
**01–1**	**1.10**	**1.34**	**1.07**	**1.29**
**01–2**	**1.22**	**1.40**	**1.09**	**1.17**
**01–3**	**1.40**	**1.43**	**1.07**	**1.36**
**01–5**	**0.79**	**0.64**	**0.62**	**0.83**
**01–6**	**1.18**	**1.41**	**0.33**	**1.23**
**01–7**	**1.18**	**1.17**	**0.69**	**1.45**
**01–8**	**1.22**	**1.42**	**0.45**	**0.94**

^*1*^Data from patient 01–4 are not included because all time points were not available

## Discussion

The DNA/MVA vaccine appeared safe and well tolerated in our cohort. Clinical and laboratory adverse events were generally mild to moderate and transient. There was no grade 4 clinical or laboratory adverse events, no serious adverse events, and no discontinuations due to adverse events. No Grade 3 adverse events were considered related to study product. Only reactogenicity events were felt to be study drug related. There was no evidence of emergent drug resistance during treatment interruption. Of note, one subject experienced a prolonged efavirenz washout, illustrating the importance of providing transitional therapy to avoid the selection of viral resistance in the setting of analytic treatment interruption of efavirenz. It is well established that certain CYP2B6 polymorphisms, found more frequently in African-Americans, are associated with delayed clearance of efavirenz[25]. There were no safety issues associated with treatment interruption. By 24 weeks following reinstitution of therapy, CD4 T cell levels had returned to baseline and all participants suppressed virus to below 50 copies/mL.

All participants in this trial experienced viral rebound following treatment interruption. Lack of randomization precludes evaluation of whether vaccine impacted time to viral reemergence, or its peak level. Time to reemergence, however, was similar to that seen in several other trials incorporating treatment interruption[26, 27] [28, 29] but shorter than seen in trials with very early initiation of therapy such as SPARTAC[30], VISCONTI,[31], and an NIAID acute infection cohort[32]. In SPARTAC, 26% maintained HIV-1 RNA below 400 copies/mL through 12 weeks of TI, with 9% and 4% maintaining suppression through 52 and 104 weeks. Even longer duration of viral suppression was seen in 14 patients in the VISCONTI cohort. This suggests that very early initiation of therapy alone, in some circumstances, may be associated with better virologic control, independent of interventions such as vaccination.

The DNA/MVA vaccine, like other vectored vaccines[33, 34], [35] [36] [37, 38] and dendritic cell vaccines [39], proved highly capable of eliciting CD8+ T and to a lesser extent, CD4+ T cells. However, neither in this study, nor the studies of others, have these elicited responses prevented viral re-emergence or provided long-term control of re-emergent virus [36, 39, 40].

Gag-specific CD8+ T cells expanded with vaccination in 8 of the 9 participants in our study and were elevated at the time of TI compared to pre-vaccination in 5 of the 8. The elicited CD8+ T cells did not appear to be exhausted based on the ability of a subset to co-express IL-2 [41] and the presence of low levels of PD1, CTLA-4 and TIM-3 inhibitory receptors[24, 42–44]. Yet, Gag-CD8+ T cell responses, as high as 2.5% of total CD8+ cells at TI failed to prevent viral rebound. Indeed, rapid expansion of CD8+ T cells in response to rebound virus, a hallmark for T cell recognition of an infection, occurred in only two participants (01–4 and 01–8). In these two individuals, the expansion correlated with a temporary decline in viremia. Sequence analysis of re-emergent virus and virus present at the time of treatment re-institution revealed the selection of mutations in known CD8+ T cell epitopes. This suggests that in 01–4 and 01–8 T cell responses may have transiently decreased viral load prior to viral escape.

The transiently decreased viremia in 01–4 and 01–8 involved greater than 10-fold reductions in plasma virus present at peak levels of greater than 10,000 (01–4) and 100,000 copies/mL (01–8). Yet decreased viremia was partial and not sustained and virus rapidly rebounded. The T cells also did not prevent viral re-emergence, a phenomenon that starts with foci of expressing cells. This could reflect the responding T cells not having access to sites of reemergence [45–47].

In contrast, 5 participants (01–1, 01–2, 01–5, 01–6 and 01–7) experienced CD8+ T cells expansion in response to vaccination but did not expand in response to re-emergent virus. One explanation for this phenomenon is that the re-emergent viruses had already escaped the T cell response that was boosted by the vaccine. T cell escape is detected within days of infection [48–50] and a substantial fraction of the latent reservoir is comprised of archived escape mutants [51]. Alternatively, our T cell assays may not have been frequent enough to detect concomitant expansions and contractions of viremia and T cells. For example, in 01–01, our two T cell assays within the period of TI did not show obvious expansions and contractions while 5 assays for viremia showed two substantial expansions and contractions. Another possibility is that the vaccine boosted exhausted CD8+ T cells. Exhaustion, however, was not evident in our analyses for co-expression of IFNγ and IL-2 or levels of PD-1, CTLA-4 and TIM-3 inhibitory receptors, which are markers of exhaustion [24, 41] This is consistent with other studies showing the restoration of functional capacity to HIV-specific T cells during prolonged ART [24].

Interestingly, after re-emergence of virus, activated CD8+ T cells (CD38+ and HLA-DR+) were about 10-times more frequent than vaccine-elicited IFN-γ+ Gag-specific CD8+ T cells (Figs 3 and 5). This suggests that fewer than 10% of the potentially active CD8+ T cells were scoring in our Gag-ICS assay. Large expansions of activated CD8+ T cells have been found to represent cells specific for the stimulating infection [52] or vaccine [53]. If this is also true for HIV infections, our vaccine-elicited Gag-specific IFNγ response represented only a fraction of the total CD8+ response against HIV.

Responses elicited by pools of Env peptides matched to our vaccine were substantially less frequent than those elicited by Gag. This leads to speculation about whether a vaccine more closely matched to the entire genome of the infecting virus could enhance control. The closest test of matched genomes has been with dendritic cells primed *ex vivo* with RNA representing the patient’s autologous virus[39]. These achieved a similar median time to viral re-emergence as observed here[54]

Transient low levels of viremia were seen in 6 of 9 participants during the vaccination phase of the trial. The phenomenon of transient viremia following vaccinations has been described previously[55–57]. In our trial, the most frequent temporal relationship of transient viremia to vaccination was at one week after DNA inoculation, when 5 instances of transient viremia occurred in 4 of 9 participants. A post hoc analysis of a clinical trial testing a pneumococcal vaccine in the presence and absence of CpG oligonucleotides showed decreases in the proviral reservoir in those receiving the toll-like receptor 9 (TLR9) agonist [58]. It is possible that the vaccine DNA might have transiently reactivated virus by its CpG sequences stimulating TLR9[59] or other innate pathways[60][58].

Our findings highlight major limitations for therapeutic vaccination. While our vaccine boosted existing responses and elicited some responses that had not been previously detected, these were not sufficient to prevent rebound viremia. In 6 of our 8 participants, the rebound viremia appeared to represent viruses that had already escaped the host’s immune response. Furthermore, in the two patients where CD8 T cell responses correlated with transient declines in viremia, high levels of re-emergent virus allowed rapid escape from the CD8+ T cell response. If therapeutic immunizations are to be successful, the patient will need to initiate antiretroviral therapy early to limit the archiving of escape virus in reservoirs [51]. At present, only rare patients are identified at the time of primary infection, or even during recent infection, thus complicating the ability of therapeutic CD8+ T cell vaccines to achieve optimal efficacy. Additionally, the elicited CD8+ T cells will need to have a broad target specificity that is not easily overcome by escape variants [61]. And finally, the CD8+ T cells will need to traffic to, and act at, the sites of re-emergence to snuff rebounding virus before sufficient replication for escape occurs[45].

## Supporting Information

S1 Fig Sequence Analysis of gag for Patient 01–04 at the Time of Viral Re-emergence and the Time of Drug Re-institution. The sequence at the top, with designated amino acids, is the consensus sequence for the re-emergent virus. Individual sequences are given for gag at the time of drug reinstitution. In these sequences, dashes indicate amino acids that are the same as in the re-emergent virus, and amino acid letters indicate changes from the sequence of the re-emergent virus. Known CD8 epitopes with clustered mutations are highlighted in yellow. Only clustered changes that occur in 50% or more of the mutated sequences are highlighted here. Other, less frequent clustered changes are also in known CD8 epitopes. CD8 epitopes were identified using the Los Alamos National Laboratories HIV Molecular Immunology database.
(TIFF)

S2 Fig Sequence Analysis of gag for Patient 01–8 at the Time of Viral Re-emergence and the Time of Drug Reinstitution. For detail, see legend to S1 Fig.
(TIFF)

S1 File Trendstatement Checklist. (PDF)

S2 File GV-TH-01 Protocol. (PDF)

S1 Table Clinical Adverse Events. (DOCX)

S2 Table Reactogenicity. (DOCX)

S3 Table Laboratory Adverse Events. (DOCX)
