# Supplementary figures and images for: DNA/MVA Vaccination of HIV-1 Infected Participants with Viral Suppression on Antiretroviral Therapy, followed by Treatment Interruption: Elicitation of Immune Responses without Control of Re-Emergent Virus
Source: PLoS One. 2016 Oct 6;11(10):e0163164. doi: 10.1371/journal.pone.0163164 (PMC5053438; doi:10.1371/journal.pone.0163164)

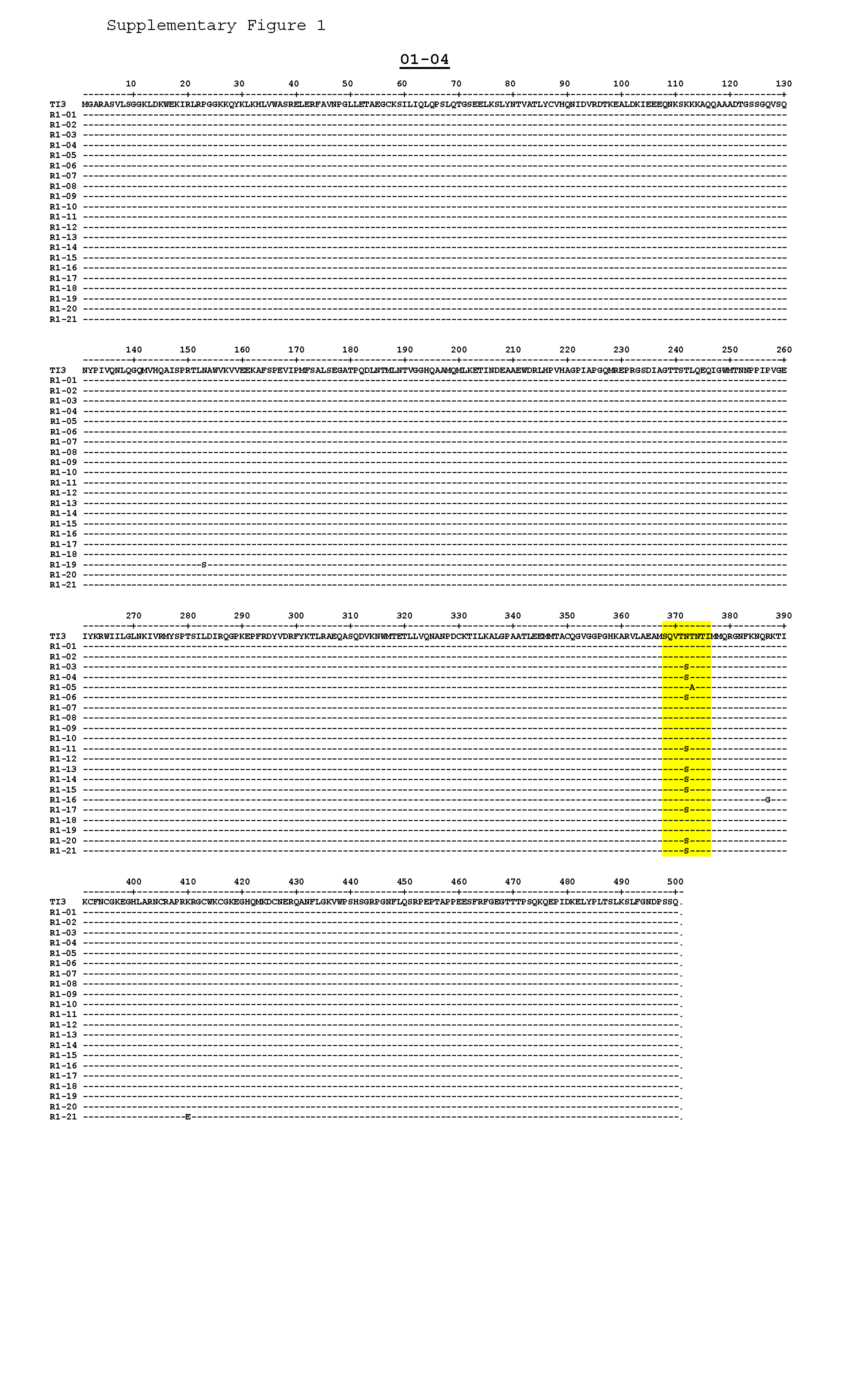

Supplement: S1 Fig — The sequence at the top, with designated amino acids, is the consensus sequence for the re-emergent virus. Individual sequences are given for gag at the time of drug reinstitution. In these sequences, dashes indicate amino acids that are the same as in the re-emergent virus, and amino acid letters indicate changes from the sequence of the re-emergent virus. Known CD8 epitopes with clustered mutations are highlighted in yellow. Only clustered changes that occur in 50% or more of the mutated sequences are highlighted here. Other, less frequent clustered changes are also in known CD8 epitopes. CD8 epitopes were identified using the Los Alamos National Laboratories HIV Molecular Immunology database. (TIFF) [file pone.0163164.s001.tiff]

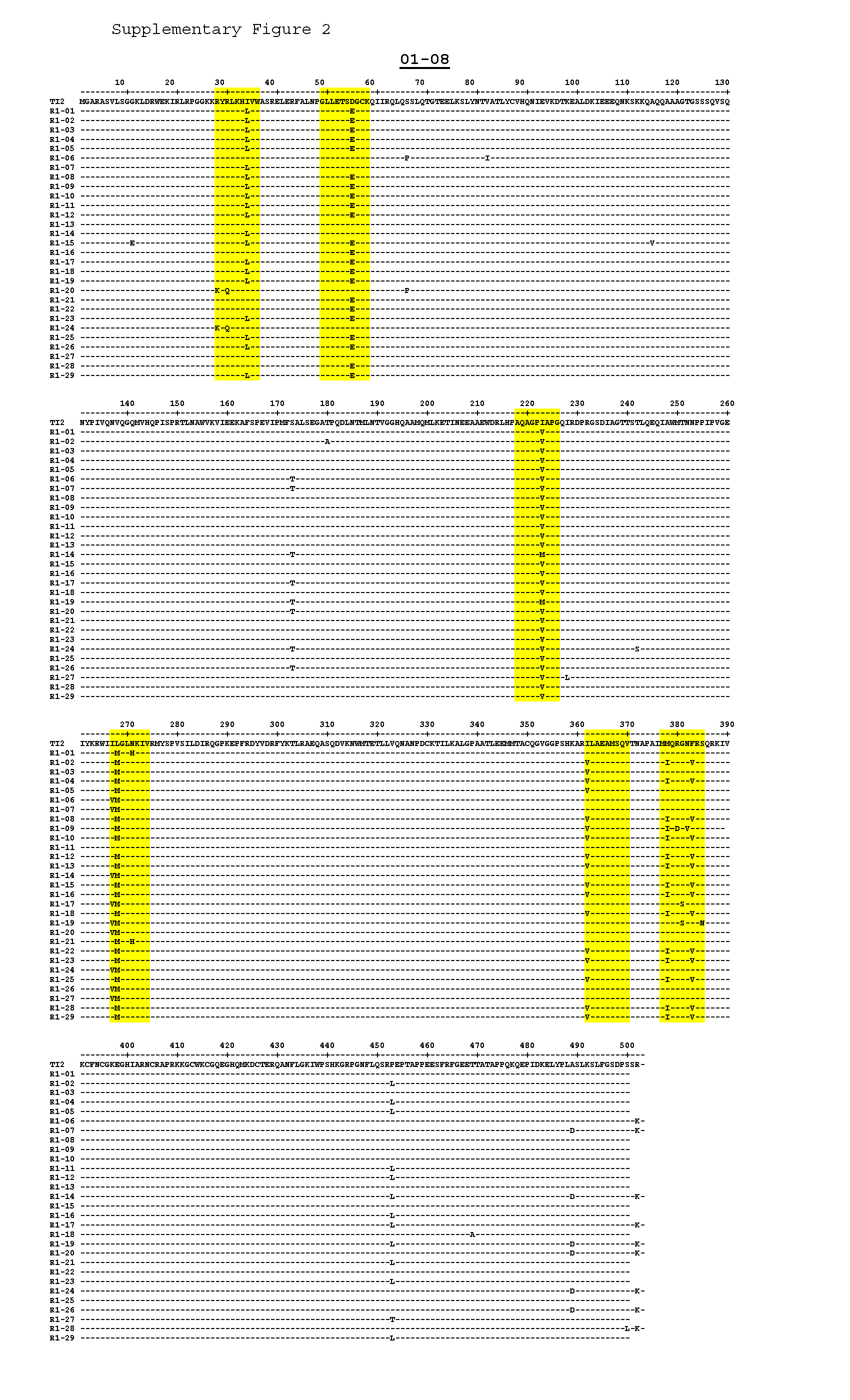

Supplement: S2 Fig — For detail, see legend to S1 Fig. (TIFF) [file pone.0163164.s002.tiff]
