# Supplementary material for: DNA/MVA Vaccination of HIV-1 Infected Participants with Viral Suppression on Antiretroviral Therapy, followed by Treatment Interruption: Elicitation of Immune Responses without Control of Re-Emergent Virus
Source: PLoS One. 2016 Oct 6;11(10):e0163164. doi: 10.1371/journal.pone.0163164 (PMC5053438; doi:10.1371/journal.pone.0163164)
Supplement: S2 File — (PDF) [file pone.0163164.s004.pdf]

CLINICAL STUDY PROTOCOL

A Phase 1 Clinical Trial to Evaluate the Safety and Immunogenicity of a Prime-boost Vaccine Regimen of pGA2/JS7 DNA and MVA/HIV62B in HIV-Infected Adults with Suppressed Viremia Who Started Antiretroviral Therapy Within Eighteen Months of a Negative HIV Antibody Test

**Sponsor:**	GeoVax, Inc.<br>1900 Lake Park Drive, Suite 380<br>Smyrna, GA 30080<br>(678) 384-7220 main number<br>(678) 384-7281 fax
**IND Number:**	14299
**Study Vaccine Name:**	*pGA2/JS7 DNA and MVA/HIV62B*
**Sponsor Protocol No.:**	GV-TH-01
**Study Medical Monitor**	Jean T. Barbey, MD<br>Social & Scientific Systems, Inc.<br>Office Phone: 301-628-3316<br>Mobile Phone: 202-251-6523<br>E-mail: jtbarbey@s-3.com
**Principal Investigators**	Melanie Thompson, MD<br>AIDS Research Consortium of Atlanta<br>Atlanta, Georgia 30308<br><br>Sonya L. Heath, MD<br>Alabama Vaccine Research Clinic<br>University of Alabama at Birmingham<br>Birmingham, Alabama 35294<br><br>Stephen Brown, MD<br>AIDS Research Alliance<br>Los Angeles, CA 90015
**Development Phase:**	I
**Date/Version of Protocol:**	**April 2014 *Version 8.0***

CONFIDENTIAL
This document contains confidential information belonging to GeoVax, Inc. No part of this document may be transferred, duplicated, referenced or recorded in any way or by any means, including electronic transmission, photocopy, or recording without written advance permission from GeoVax, Inc.

SIGNATURE PAGE

Declaration of Sponsor

GV-TH-01: A Phase 1 Clinical Trial to Evaluate the Safety and Immunogenicity of a Prime-boost Vaccine Regimen of pGA2/JS7 DNA and MVA/HIV62B in HIV-Infected Adults with Suppressed Viremia Who Started Antiretroviral Therapy Within Eighteen Months of A Negative HIV Antibody Test.

Date and Version: April 2014, Version 8

This study protocol was subjected to critical review and approved by GeoVax, Inc. The information it contains is consistent with current knowledge of the risks and benefits of the investigational product, as well as with the moral, ethical, and scientific principles governing clinical research as set out in the Declaration of Helsinki, as amended in 1996, and the guidelines on Good Clinical Practice.

____

Harriet Robinson, PhD
GeoVax, Inc.
1900 Lake Park Drive
Suite 380
Smyrna, GA 30080
(678) 384-7220 office telephone
(678) 384-7281 fax

Declaration of the Principal Investigator

Title: A Phase 1 Clinical Trial to Evaluate the Safety and Immunogenicity of a Prime-boost Vaccine Regimen of pGA2/JS7 DNA and MVA/HIV62B in HIV-Infected Adults with Suppressed Viremia Who Started Antiretroviral Therapy Within Eighteen Months of a Negative Antibody Test

This study protocol was subjected to critical review and has been approved by GeoVax, Inc. The information it contains is consistent with the current risk/benefit evaluation of the investigational product as well as with the moral, ethical and scientific principles governing clinical research as set out in the Declaration of Helsinki, as amended in 1996 and the guidelines on Good Clinical Practice.

I have read the protocol including all of the appendices and I agree to conduct the study as outlined herein. Personnel under my supervision will conduct the study according to the protocol and will be familiar with all materials provided to us by GeoVax, Inc.

Principal Investigator

____ ____________________

Name: Melanie Thompson, MD **Date**
Title: Principal Investigator
Institution: AIDS Research Consortium of Atlanta
131 Ponce de Leon Avenue, NE
Suite 130
Atlanta, Georgia 30308

Declaration of the Principal Investigator

Title: A Phase 1 Clinical Trial to Evaluate the Safety and Immunogenicity of a Prime-boost Vaccine Regimen of pGA2/JS7 DNA and MVA/HIV62B in HIV-Infected Adults with Suppressed Viremia Who Started Antiretroviral Therapy Within Eighteen Months of a Negative Antibody Test

This study protocol was subjected to critical review and has been approved by GeoVax, Inc. The information it contains is consistent with the current risk/benefit evaluation of the investigational product as well as with the moral, ethical and scientific principles governing clinical research as set out in the Declaration of Helsinki, as amended in 1996 and the guidelines on Good Clinical Practice.

I have read the protocol including all of the appendices and I agree to conduct the study as outlined herein. Personnel under my supervision will conduct the study according to the protocol and will be familiar with all materials provided to us by GeoVax, Inc.

Principal Investigator

____ ____________________

Name: Sonya L. Heath, MD **Date**
Title: Principal Investigator
Institution: The University of Alabama at Birmingham (UAB)
Alabama Vaccine Research Clinic
Room 328B Community Care Building
908 20th Street, South
Birmingham, Alabama 35294

Declaration of the Principal Investigator

Title: A Phase 1 Clinical Trial to Evaluate the Safety and Immunogenicity of a Prime-boost Vaccine Regimen of pGA2/JS7 DNA and MVA/HIV62B in HIV-Infected Adults with Suppressed Viremia Who Started Antiretroviral Therapy Within Eighteen Months of a Negative Antibody Test

This study protocol was subjected to critical review and has been approved by GeoVax, Inc. The information it contains is consistent with the current risk/benefit evaluation of the investigational product as well as with the moral, ethical and scientific principles governing clinical research as set out in the Declaration of Helsinki, as amended in 1996 and the guidelines on Good Clinical Practice.

I have read the protocol including all of the appendices and I agree to conduct the study as outlined herein. Personnel under my supervision will conduct the study according to the protocol and will be familiar with all materials provided to us by GeoVax, Inc.

Principal Investigator

____ ____________________

Name: Stephen Brown, MD **Date**
Title: Principal Investigator
Institution: AIDS Research Alliance
1400 S. Grand Ave
Suite 701
Los Angeles, CA 90015

STUDY CONTACT INFORMATION

SPONSOR
GeoVax, Inc.
1900 Lake Park Drive
Suite 380
Smyrna, GA 30080
(678) 384-7220 Office telephone
(678) 384-7281 Fax

MEDICAL MONITOR
Jean T. Barbey, MD
Medical Director/Monitor
Social & Scientific Systems, Inc.
8757 Georgia Avenue, Room #9030
Silver Spring, MD 20910
Office Phone : 301-628-3316
Mobile Phone: 202-251-6523
E-mail: jtbarbey@s-3.com

SPONSOR PROJECT MANAGER
Harriet Robinson, PhD
GeoVax, Inc.
1900 Lake Park Drive
Suite 380
Smyrna, GA 30080
(678) 384-7220 Office telephone
(678) 384-7281 FAX

PRINCIPAL INVESTIGATORS

Melanie Thompson, MD
AIDS Research Consortium of Atlanta
131 Ponce de Leon Avenue, NE
Suite 130
Atlanta, GA 30308
Office Phone: 404-876-2317
Mobile Phone: 404-276-7720
E-mail: drmt@mindspring.com

Sonya L. Heath, MD
The University of Alabama at Birmingham
Alabama Vaccine Research Clinic
Room 328B Community Care Building
908 20th Street, South
Birmingham, Alabama 35294
Office Phone: 205-996-2371
E-mail: heaths@uab.edu

Stephen Brown, MD
Vice President & Medical Director
AIDS Research Alliance
1400 South Grand Avenue
Suite 701
Los Angeles, CA 90015
Office Phone: 310-358-2428
E-mail: s.brown@aidsresearch.org

ACRONYMS AND ABBREVIATIONS

AACTG A5197	therapeutic study in HIV-1 infected subjects, sponsored by the Adult AIDS Clinical Trials Group that has a 16 week treatment interruption
ADCVI	antibody-dependent cell mediated virus inhibition
AE	adverse event
ALT	alanine aminotransferase
ANC	absolute neutrophil count
Anti-HCV antibody	anti-hepatitis C virus antibody
ARA	AIDS Research Alliance
ARCA	AIDS Research Consortium of Atlanta
ART	antiretroviral treatment
AVRC	Alabama Vaccine Research Clinic
bDNA	branched DNA
β-HCG	beta human chorionic gonadotropin
BL	baseline
BPI	boosted protease inhibitor
BUN	blood urea nitrogen
C	Centigrade
CD4	cell surface marker for helper T cell
CD8	cell surface marker for cytotoxic T cell
CD38	cell surface marker of T-cell activation
CD40	co-stimulatory protein found on antigen-presenting cells
CD40L	protein primarily expressed on activated cells
CD3/4	cell surface markers that define the helper T cell phenotype
CD3/8	cell surface markers that define the cytotoxic T cell phenotype
CD19	cell surface markers that define the B cell phenotype
CD8/CD38/DR	cell surface makers that define activation of CD8+ T cells
CD4/CD38/HLA-DR	surface makers that define activation of CD4+ T cells
CCR5	chemokine receptor which HIV-1 uses to gain entry into macrophages
CDC	US Centers for Disease Control and Prevention
CEF	chick embryo fibroblasts

CMP	complete metabolic profile
CPK	creatine phosphokinase
CRF	case report forms
cGMP	current Good Manufacturing Practice
CMV	cytomegalovirus
CRF	case report form
CVA	chorioallantois vaccinia Ankara
DAIDS	Division of AIDS (US NIH)
d-Dimer	fibrin degradation product associated with inflammation
DNA	deoxyribonucleic acid
DSMB	Data Safety Monitoring Board
E. coli DH5αT-1	host cell for recombinant manufacture of pGA2/JS7 DNA plasmid vaccine
ECG	electrocardiogram
EDTA	ethylene diaminetetraacetic acid
ELISA	enzyme-linked immunosorbent assay
Env	HIV-1 envelope protein
env	HIV-1 envelope gene
F	Fahrenheit
Fc receptor	receptor that binds the Fc region of antibody, found on surface of natural killer cells, macrophages, neutrophils and mast cells
FDA	US Food and Drug Administration
g/dL	grams per deciliter
Gag	HIV-1 core protein
gag	HIV-1 core gene
GCP	Good Clinical Practice
GLP	Good Laboratory Practice
HBsAg	hepatitis B surface antigen
HCV	hepatitis C virus
HDL	high density lipoprotein
HIPAA	Health Information Portability and Accountability Act
HIV-1	Human Immunodeficiency Virus Type 1
HIV-MN	Laboratory adapted HIV-1 strain belonging to clade B
hsCRP	highly sensitive C-reactive protein, a marker of inflammation

HVTN	HIV Vaccine Trials Network
ICH	International Conference on Harmonisation of Technical Requirements for Registration of Pharmaceuticals for Human Use
ICS	intracellular cytokine staining, a method of scoring responding T cells
ID	intradermal
IFN-γ	interferon gamma
IL-2	interleukin-2
IL-6	interleukin-6, marker for inflammation
IM	intramuscular
IND	Investigational New Drug
IR	intrarectal
IRB	Institutional Review Board
ITT	intent-to-treat
IUD	intrauterine device
LDL	low density lipoprotein
MedDRA	Medical Dictionary for Regulatory Activities (clinically validated international medical terminology used throughout the regulatory process)
mer	the number of amino acids in a peptide
MI	myocardial infarction
µg	microgram
mg	milligram
min	minute
mL	milliliter
MVA	Modified Vaccinia Ankara
MVA/HIV62B	designation for recombinant MVA vaccine study product
ng	nanogram
Nef	HIV-1 protein
NIAID	National Institute of Allergy and Infectious Diseases (US NIH)
NIH	US National Institutes of Health
P450 2B6	Cytochrome P450 Cyp2B6 – main catalyst of efavirenz primary and secondary metabolism
PBMC	peripheral blood mononuclear cell
PBS	phosphate-buffered saline
PCR	polymerase chain reaction

pfu	plaque forming unit
pGA2/JS7 DNA	designation for recombinant DNA vaccine study product
PI	Principal Investigator
Pol	HIV-1 polymerase protein
pol	HIV-1 polymerase gene
PP	per-protocol
PR	HIV-1 protease protein
PR interval	defined as the period that extends from the onset of atrial depolarization (beginning of the P wave) until the onset of ventricular depolarization
PTE	peptides designed to represent potential T-cell epitopes
QT interval	measure of time between start of Q wave and end of T wave
QTc	heart rate-corrected QT interval
Rev	HIV-1 protein
RNA	ribonucleic acid
RT	reverse transcriptase
RT-PCR	real time polymerase chain reaction
SAE	serious adverse event
SHIV	chimeric simian human immunodeficiency virus
SIV	simian immunodeficiency virus
SIV239	simian immunodeficiency virus 239
SIV251	simian immunodeficiency virus 251
SMART	Strategies for Management of Anti-retroviral Therapy – a large-scale therapeutic study of scheduled treatment interruption in HIV-1 infected subjects
SOP	standard operating procedure
SPF	specific pathogen free
SUSAR	suspected unexpected serious adverse reaction
Tat	HIV-1 protein
$TCID_{50}$	50% tissue culture infectious dose
TNFα	tumor necrosis factor alpha
UA	urinalysis
UAB	The University of Alabama at Birmingham
ULN	upper limit of normal

TABLE OF CONTENTS

Section	Heading	Page
**1.**	**OVERVIEW**	**17**
1.1	Protocol Synopsis	**17**
1.2	Protocol Team	**19**
**2.**	**BACKGROUND**	**20**
2.1	Study Product Description	**20**
2.1.1	pGA2/JS7 DNA Vaccine	**21**
2.1.2	MVA/HIV62B Vaccine	**21**
2.2	Preclinical Therapeutic Studies	**22**
2.3	Previous Clinical Studies with JS7 DNA and MVA62B Vaccines	**26**
2.3.1	Safety Results in HVTN 065	**27**
2.3.2	Immunogenicity Results for HVTN 065	**31**
2.3.2.1	Immunogenicity Summary of T Cell Responses	**32**
2.3.2.2	HVTN 065 Immunogenicity Summary of Antibody Responses	**32**
2.3.3	Introduction to HVTN 205	**33**
2.3.4	HVTN 205 Safety	**34**
2.3.4.1	Reactogenicity	**34**
2.3.4.2	Adverse Events	**35**
2.3.4.2.1	Grade 3, Serious, or Life-threatening Adverse Events	**35**
2.3.4.2.2	Mild and Moderate Adverse Events Related to Vaccination	**35**
2.3.5	Immunogenicity of Study Products in HVTN 205	**37**
2.3.5.1	Response Rates for Antibody and T Cell Responses	**37**
2.3.5.1.1	Introduction to HVTN 205 Antibody and T Cell Responses	**37**
2.3.5.1.2	T Cell Responses	**37**
2.3.5.1.3	Antibody Responses	**39**
2.3.5.2	Summary of Prior Preclinical and Immunogenicity Response Data	**41**
2.4	Rationale for Current Trial Design	**43**
2.4.1	Rationale for Study Population Selection	**43**
2.4.2	Rationale for Design of Vaccination Phase	**44**
2.4.3	Rationale for Design of the Analytic Treatment Interruption Phase Including Discontinuation of Efavirenz	**45**
2.4.4	Rationale for Design of the Treatment Reinstitution Phase	**46**
**3.**	**STUDY DESIGN**	**47**
3.1	Vaccination Phase	**47**
3.2	Analytic Treatment Interruption Phase	**47**
3.3	Treatment Reinstitution Phase	**47**

**4.**	**STUDY OBJECTIVES**	**48**
4.1	Primary and Secondary Objectives	**48**
4.2	Exploratory Objectives	**48**
**5.**	**STUDY POPULATION**	**49**
5.1	Inclusion Criteria	**49**
5.2	Exclusion Criteria	**50**
**6.**	**STUDY PRODUCT PREPARATION AND ADMINISTRATION**	**51**
6.1	Study Product Formulation	**51**
6.1.1	pGA2/JS7 DNA	**51**
6.1.2	MVA/HIV62B	**51**
6.2	Preparation, Administration, and Dispensing of Study Products	**51**
6.2.1	Preparation of pGA2/JS7 DNA	**52**
6.2.2	Preparation of MVA/HIV62B 1 x 10^8 $TCID_{50}$	**52**
6.3	Administration	**53**
6.4	Dispensing of Boosted protease Inhibitor (BPI) Regimen for Patients on Efavirenz	**53**
7.	**CLINICAL ASSESSMENTS**	**53**
7.1	The Schedule of Clinical Procedures (Appendix A)	**53**
7.2	Informed Consent	**53**
7.3	Patient Screening	**53**
7.3.1	Screening Visit	**54**
7.4	Enrollment and Study Visits	**54**
7.4.1	Vaccination Phase	**54**
7.4.2	Discontinuation of ART for those on Efavirenz: Schedule of Visits	**61**
7.4.3	Analytic Treatment Interruption Phase: Schedule of Study Visits	**61**
7.4.4	Treatment Reinstitution: Schedule of Study Visits	**65**
7.4.5	Procedure for ART Discontinuation for Patients Not on Efavirenz	**67**
7.4.6	Procedure for Switching to BPI or Raltegravir for Patients on Efavirenz	**68**
7.5	Cardiac Safety Monitoring	**68**
7.5.1	Framingham Global Risk Assessment	**68**
7.5.2	Cardiac Symptoms Assessment	**68**
7.5.3	ECG Testing	**68**
7.5.4	Evaluation of Elevated Creatine Phosphokinase (CPK)	**68**
7.5.5	Evaluation of Suspected Myo/pericarditis	**70**
7.6	Evaluation of Suspected MVA Replication	**70**
7.7	Change in Vaccination Schedule	**71**
7.8	Visit Window and Missed Visits	**71**

7.9	Vaccination Discontinuation	**71**
7.10	Concomitant Medications	**72**
7.11	Assessment of Reactogenicity	**72**
7.11.1	Assessment of Systemic and Local Symptoms	**72**
7.12	Pregnancy	**72**
7.13	Definitions of Virologic Failure	**73**
7.14	Definitions of Immunologic Failure	**73**
7.15	Criteria for Reinstitution of Treatment During Analytic Treatment Interruption Phase	**73**
**8.**	**LABORATORY**	**73**
8.1	Safety Laboratory Assessments	**73**
8.2	Virology Assays	**74**
8.3	Immunology Assays	**74**
8.3.1	Cellular Immune Responses	**74**
8.3.2	Humoral	**75**
8.4	Human Leukocyte Antigen (HLA) Tissue Typing	**75**
8.5	Inflammatory Marker Assays	**75**
8.6	Pharmacology Assays	**75**
8.7	Future Use of Stored Specimens	**75**
8.8	Total Blood Volume	**76**
8.9	Biohazard Containment	**76**
**9.**	**STATISTICAL ANALYSIS**	**76**
9.1	Analysis of Primary, Secondary, and Exploratory Endpoints	**76**
9.1.1	Primary Endpoint: Safety	**76**
9.1.2	Secondary Endpoints	**77**
9.1.3	Exploratory Endpoints	**78**
9.2	Analysis Data Sets	**79**
9.2.1	Intent-to-Treat Analysis	**79**
9.2.2	Per-Protocol Analysis	**79**
9.2.3	Statistical Methods	**79**
9.3	Sample Size and Power Calculations	**79**
**10.**	**SAFETY MONITORING AND SAFETY REVIEW**	**79**
10.1	Adverse Events	**79**
10.1.1	Definitions	**80**
10.2	Classification and Reporting of Adverse Events	**80**
10.3	Emergency Contact	**82**
10.4	Pregnancy	**82**
10.5	Sponsor Reporting of Serious Adverse Events	**83**
10.6	Monitoring of Adverse and Serious Adverse Events	**83**

**11.**	**DATA MANAGEMENT**	**83**
11.1	Data Collection	**83**
11.2	Data Monitoring	**84**
11.3	Confidentiality	**84**
**12.**	**INVESTIGATOR AND SPONSOR RESPONSIBILITIES**	**84**
12.1	Investigator Responsibilities	**84**
12.1.1	Protocol Adherence	**84**
12.1.2	Delegation of Authority	**84**
12.1.3	Regulatory Approval	**84**
12.1.4	Informed Consent	**85**
12.1.4.1	Pre-Screening Informed Consent	**85**
12.1.5	Drug Accountability	**85**
12.1.6	Record Retention	**85**
12.2	Sponsor Responsibilities	**86**
12.2.1	Protocol Modifications	**86**
12.2.2	Study Report and Publications	**86**
12.2.3	Conduct of Study in Accord with FDA Requirements	**86**
12.2.4	Retention and Destruction of Stored Samples	**86**
12.3	Shared Responsibilities of the Investigator and the Sponsor	**86**
12.3.1	Ethical Study Conduct	**86**
12.3.2	Confidentiality	**86**
12.3.3	Premature Study Discontinuation	**87**
**13.**	**REFERENCES**	**88**

APPENDICES

A. Informed Consent Form
B. Schedule of Events
C. Case Definition of Myocarditis/Pericarditis for Use in Adverse Events Monitoring
D. Division of AIDS Table for Grading the Severity of Adult and Pediatric Adverse Events
E. Framingham Heart Study Coronary Heart Disease Risk Assessment Tool

List of Tables Page

Table 1. Summary of therapeutic trials conducted with prototype SIV239 DNA and MVA vaccines 24
Table 2. HVTN 065 Schema 26
Table 3. Trial Schema for HVTN 205 27
Table 4. Maximum severity of reactogenicity symptoms by treatment group in Parts A and B, across all vaccinations 29
Table 5. Grade 3 (severe) or Grade 4 (life-threatening) adverse events (AE) in placebo, Part A, and Part B treatment groups 30
Table 6. Parts A and B: Vaccination completion and reasons for early discontinuation of vaccination 31

List of Figures

Figure 1. Logarithmic differences in levels of viral RNA at the initiation of ART and at 12 or 24 weeks post- treatment interruption for animals placed on ART at different times post infection 25
Figure 2. Immune response rates determined in HVTN end point assays 32
Figure 3. Magnitudes and response rates of Env binding and neutralizing antibodies 33
Figure 4. Local pain and/or tenderness and maximum systemic reactogenicity in HVTN 205 (Unblinded data) 36
Figure 5. Response rates for vaccine-elicited T cells and antibody in HVTN 205 37
Figure 6. Specificity of elicited T cell response 38
Figure 7. Magnitude of CD4+ T cell responses 38
Figure 8. Magnitude of CD8+ T cell responses 39
Figure 9. Antibody responses to Env and Gag 40
Figure 10. Vaccine-elicited IgA responses in serum 40
Figure 11. Breadth of neutralizing antibody responses elicited in DDMM and MMM vaccine groups 41
Figure 12. Titer of antibody responses to Tier 2 isolates 41
Figure 13. Schematic for therapeutic trial showing study phases 48

1. OVERVIEW

1.1 Protocol Synopsis

Protocol Title
A Phase 1 clinical trial to evaluate the safety and immunogenicity of a prime-boost vaccine regimen of pGA2/JS7 DNA and MVA/HIV62B in HIV-infected adults with suppressed viremia who started antiretroviral therapy within eighteen months of a negative HIV antibody test.

Brief Description of Protocol
GV-TH-01 is an open label Phase 1 study involving 9 HIV-1 infected adults with suppressed viremia who started on antiretroviral therapy within 18 months of a documented negative HIV-1 antibody test. Participants will be vaccinated with a DNA plasmid vaccine, designated pGA2/JS7 DNA, encoding Gag, Pol, and Env at Baseline (BL, week 1) and week 9 and boosted with a Modified Vaccinia Ankara (MVA) vaccine, designated MVA/HIV62B, encoding matched Gag, Pol, and Env sequences at weeks 17 and 25. At 8 weeks following the last vaccination (or longer for some patients taking efavirenz), ART will be interrupted for a 12 week period following which ART will be reinstituted and the participants followed for an additional 24 weeks. The goal of this Phase 1 clinical trial is to test safety and immunogenicity during each of the three phases of the trial: vaccination, analytic treatment interruption and treatment reinstitution.

Study Objectives

Primary Objective

- To evaluate the safety of the therapeutic use of pGA2/JS7 DNA and MVA/HIV62B vaccines during vaccination, analytic treatment interruption and treatment reinstitution

Secondary Objectives

- To evaluate the immunogenicity of the pGA2/JS7 DNA and MVA/HIV62B vaccines during the vaccination phase of the trial
- To evaluate HIV-1 RNA levels and CD4+ T cell counts during analytic treatment interruption
- To evaluate HIV-1-specific immune responses during the analytic treatment interruption phase of the trial

Exploratory Objectives

- To explore possible correlations between peak post vaccination immune responses and the control of re-emergent virus during analytic treatment interruption

- To explore possible correlations between control of re-emergent virus levels of markers for inflammation and HIV-1 RNA during the period of analytic treatment interruption
- To explore levels of HIV-1 RNA prior to the initiation of ART and levels in the analytic treatment interruption phase of the trial

Study Products and Routes of Administration

- *DNA vaccine*: pGA2/JS7 DNA (JS7) is a 9.5 kb plasmid DNA expressing the HIV-1 proteins Gag, Pol (PR, RT), Env, Tat, Rev, and Vpu, from a single transcript.
- *MVA vaccine*: Recombinant modified vaccinia Ankara/ HIV-1 clade B *gag-pol-env* (MVA/HIV62B) is a highly attenuated vaccinia virus expressing HIV-1 *gag, pol*, and *env* genes from the same sequences used to construct the JS7 DNA vaccine.
- *Administration*: Intramuscular (IM) injection with needle and syringe (entire contents of vial up to 1 mL administered at a single site in the deltoid muscle).

Participants

Ten participants (9 enrolled), defined as those who receive all immunizations and undergo analytic treatment interruption as scheduled or who meet a criterion for failure before doing so, will be enrolled in this study. These subjects will be HIV-1-infected volunteers ages 18 to 50 years with suppressed viremia (< 50 or 75 copies/mL depending upon assay used) who initiated ART within 18 months of a negative HIV-1 antibody test, have maintained suppressed viremia while on ART, have CD4+ cells > 500 cells/µL and a CD4+ cell nadir greater than 350 cells/µL unless measured in the setting of acute infection.

Study Design

Multicenter, single arm, open label:

- Vaccinations at week 1 (BL), 9, 17, and 25 for all patients
- Analytic treatment interruption for 12 weeks will begin at week 33 for patients not on efavirenz. Patients on efavirenz will be switched to a boosted protease inhibitor or raltegravir regimen at week 31 with serial measurement of efavirenz serum levels. Once efavirenz has disappeared from serum, analytic treatment interruption for 12 weeks will begin. Although not recommended, if a patient declines to restart treatment after 12 weeks because of prolonged viral suppression, the sponsor may allow the patient to continue in the study in an the extension of the treatment interruption phase if the primary care physician and Principal Investigator agree. Treatment reinstitution and follow-up for 24 weeks will begin immediately after the analytic treatment interruption.

Study Duration Per Participant

The maximum time on the study for individual patients will be 69-77 weeks.

- Vaccination Phase: 32 weeks (eight weeks following each of four immunizations)
- Efavirenz Wash Out: 2 to 8 weeks depending on rate of decline of efavirenz serum levels.

- Analytic Treatment Interruption Phase: 12 weeks (or longer if extended)
- Treatment Reinstitution Phase: 24 weeks.

Estimated total study duration
Approximately 155 weeks including 78 weeks for enrollment and 77 weeks (maximum) follow-up for the final enrolled patient. Total study duration may be longer than 155 weeks if the efavirenz wash out or analytic treatment interruption phase is extended.

Investigational New Drug (IND) sponsor
GeoVax, Inc., Smyrna, Georgia, 30080 USA

Vaccine Provider
GeoVax, Inc., Smyrna, Georgia, 30080 USA

Contract Research Organization
Social & Scientific Systems, Inc.
1009 Slater Road, Suite 120
Durham, NC 27703

Data Monitoring and Statistics
Social & Scientific Systems, Inc.
1009 Slater Road, Suite 120
Durham, NC 27703

Study Sites
AIDS Research Consortium of Atlanta (ARCA), Atlanta, Georgia, USA
Alabama Vaccine Research Clinic (AVRC), The University of Alabama at Birmingham (UAB), Birmingham, Alabama, USA
AIDS Research Alliance (ARA), Los Angeles, California, USA

1.2 Protocol Team

ARCA

Melanie Thompson, MD – Principal Investigator
Bentley Sweeton, RPh – Study Coordinator
Dacenta Grice, PA-C – Sub-investigator
Bentley Sweeton, RPh – Pharmacist
Kathy Williams, CLT (NCA) – Clinical Laboratory

UAB – AVRC

Sonya Heath, MD – Principal Investigator
Paul Goepfert, MD – Sub-investigator
John Gnann, MD – Sub-investigator
Susan Duncan, MSN, RN, NP

ARA

Stephen Brown, MD – Principal Investigator
Miles Viljanen – Assistant Clinical Research Coordinator
Chassity Griffin – Regulatory Affairs
Kevin Griggs, PhD – Research Pharmacist
Michele Vertucci, PA/NP
Robert Winters, MD - Sub-investigator

GeoVax

Harriet Robinson, PhD – Chief Scientific Officer
Robert McNally, PhD – CEO
Suzanne Smith, MS – Director, Regulatory Affairs
Suefen Kwa, Ph.D, Assistant Director

2. BACKGROUND

While substantial progress in the reduction of HIV-1-related morbidity and mortality has resulted from advances in combination antiretroviral therapy, the challenges of viral resistance, accumulated drug toxicity, potential of limited access for some individuals to expensive treatments and appropriate monitoring, and the need for a lifetime of stringent regimen adherence continue to limit the benefits of current approaches to treatment. In theory, a therapeutic HIV-1 vaccine could reduce these obstacles through enhanced immune system mediated control of HIV-1 replication, therefore eliminating, significantly delaying, or reducing the need for antiretroviral therapy. Exploration of this hypothesis first requires demonstration of vaccine safety and immunogenicity in HIV-1 infected individuals. In addition, preliminary evidence for the ability of vaccine induced immune responses to control HIV-1 replication must also be demonstrated in a small, intensely studied cohort of patients before larger trials to evaluate efficacy are initiated.

2.1 Study Product Description

Two vaccine products, which are designed for sequential use in individual patients, are described here:

- The first, a plasmid DNA vaccine, pGA2/JS7 (JS7), encodes HIV-1 *gag, pol,* and *env* and was manufactured using cGMP regulations and facilities by Althea Inc., San Diego, California, USA.
- The second product, MVA/HIV62B (MVA62B vaccine), encodes HIV-1 *gag, pol,* and *env* from the same sequences used to construct the JS7 DNA vaccine. This highly attenuated recombinant vaccinia virus was manufactured using cGMP regulations and facilities by BioReliance Ltd, Glasgow, Scotland.

Both vaccine products were manufactured under contract to GeoVax, Inc.

2.1.1 pGA2/JS7 DNA Vaccine

The HIV-1 plasmid DNA vaccine, designated JS7, is a 9.5 kb plasmid DNA composed of a 2.9 kb expression vector termed pGA2 and a 6.6 kb vaccine insert termed JS7(1). The subgenomic splicing is *rev*-dependent and uses the *rev* response element and splicing signals present in the wild type HIV-1 genome. The vaccine insert has a Col E 1 origin of replication, the kanamycin resistance gene and the lambda terminator for stabilizing inserts. Expression of the vaccine insert is driven by a 687 bp sequence from the CMV immediate early promoter and terminated by a 222 bp polyadenylation sequence from the bovine growth hormone.

The 6.6 kb JS7 vaccine insert expresses *pol* sequences of the BH10 strain of HIV-1 IIIB; *tat, rev, vpu*, and *env* sequences from a recombinant of HXB-2 and ADA HIV-1 sequences; and *gag* from HIV-1 HXB-2. The expressed Env uses CCR5 and is largely derived from the macrophage-tropic *env* gene of the ADA primary isolate.

The vaccine product was rendered noninfectious by deletions and/or point mutations in the HIV-1 sequences which include alterations of both long terminal repeats, a portion of the 5′ sequences controlling encapsidation of viral RNA and coding sequences for integrase, Vif, Vpr, and Nef. With the exception of the HIV-1 and kanamycin resistance genes, there are no known protein coding sequences within the JS7 plasmid DNA.

The JS7 DNA vaccine was manufactured using the *E. coli* DH5αT-1 bacterial strain. The JS7 DNA vaccine is supplied as a clear, colorless solution in single-dose vials containing sufficient volume to deliver 1 mL of solution containing 3 mg DNA. The vaccine is formulated in a buffer consisting of PBS, 0.2 mM EDTA and 1% v/v ethanol, pH 7.4.

2.1.2 MVA/HIV62B Vaccine

The recombinant viral vaccine, Modified Vaccinia Ankara (MVA62B), is a highly attenuated vaccinia virus encoding HIV-1 *gag, pol,* and *env* genes from the same sequences used to construct the JS7 DNA (2). MVA62B contains the same mutations in Pol as JS7 DNA. It does not contain the Gag and PR mutations of JS7 DNA. The *env* gene is truncated to remove 115 amino acids of its cytoplasmic domain; a modification that supports higher surface expression of Env which increases immunogenicity (2).

MVA was first produced in Germany in 1975 as a smallpox vaccine for individuals considered to be poor risks for the standard vaccinia inoculation (3). MVA originated from the dermovaccinia strain chorioallantois vaccinia Ankara (CVA) that was retained for many years at the Ankara Vaccination Station via donkey-calf-donkey passages. In 1953, Mayr and colleagues purified CVA and passaged it twice through cattle. In 1954/55, this purified product was used in the Federal Republic of Germany as a smallpox vaccine. In 1958, attenuation experiments with CVA were completed by terminal dilution and passaging in chick embryo fibroblasts (CEF). After 360 passages, the virus was cloned by three successive plaque purifications and maintained in CEF to 570 passages. After 570 passages, the virus was plaque purified on cells from a recognized leucosis-free flock of chickens.

In the process of serial passages in CEF, 9% of the genomic DNA was lost from the original CVA strain and the virulence of the virus for mammalian cells was greatly reduced (4, 5). In particular, the resulting strain undergoes an abortive infection in human cells. After 516 passages, the virus was designated "modified vaccinia virus Ankara (MVA)" and was given to the German State Institution, Bayerische Landesimpfanstalt, where human clinical trials were conducted with doses as high as 2×10^6 pfu (6). MVA virus from the 572nd passage in primary CEF which had been harvested on February 22, 1974, prior to documented occurrence of bovine spongiform encephalopathy, was sent directly from Professor Anton Mayr (Institute of Microbiology, University of Munich, Germany) to Dr. Bernard Moss at the National Institute of Allergy and Infectious Diseases (NIAID) in August 2001. The reconstituted virus was plaque purified three times by terminal dilutions in chicken embryonic fibroblast cells (CEF) from 10-day-old specific pathogen free (SPF) fertile chicken eggs, and certified reagents; including gamma irradiated fetal calf serum (from sources free of bovine spongiform encephalopathy) and trypsin. This MVA virus was used to prepare the current recombinant MVA62B construct.

2.2 Preclinical Therapeutic Studies

Preclinical studies were conducted with JS7 DNA and MVA62B as well as with SHIV and SIV prototypes for these vaccines. These studies addressed:

- Immunogenicity and protection against SHIV or SIV challenge infections in rhesus macaques (7-16)
- The effect of pre-existing immunity to vaccinia virus on immunizations and vaccine induced protection in rhesus macaques (17)
- The ability of the vaccine to serve as a therapy for infected rhesus macaques (Amara et al, study report dated November 11, 2009 – see Section 8, Pharmacology and toxicology information)
- Toxicology and biodistribution concerns using studies completed with New Zealand white rabbits.

A summary of the therapeutic preclinical studies is provided in the Investigator's Brochure. Here we briefly present the results of the preclinical therapeutic studies that provide the rationale for this protocol.

Preliminary therapeutic vaccination studies were completed using simian immunodeficiency virus (SIV)-infected rhesus macaques and SIV-prototypes of the GeoVax JS7 DNA and MVA62B vaccines encoding SIV239 sequences instead of HIV-1 sequences. For infection the closely related SIV239 and SIV251 viruses were used; SIV239 and SIV251 infections are the most broadly accepted model for HIV-1 infections. They are highly virulent CCR5 tropic viruses that cause progression to AIDS in most infected animals within one year of infection. Similar to HIV-1, SIV infections cause a progressive loss of viral control due to the destruction of CD4+ T cells, CD8+ T cell exhaustion, and the selection of viral mutations that result in CD8+ T cell and neutralizing antibody escape (18-21). The rapid onset of SIV-induced AIDS in rhesus macaques contrasts with most HIV-1 infections where AIDS does not develop until 8 to 10 years of infection. However this accelerated pathogenesis increases the utility of the SIV-rhesus macaque model for testing HIV-1 vaccines.

Four independent therapeutic studies were conducted; three in which infected rhesus were drug treated and then vaccinated in the presence of drug treatment (groups TH-12, TH-18, and TH-32), and one in which infected rhesus were drug-treated but not vaccinated (group TH-C) (Table 1). The vaccination studies designated TH-12, TH-18, and TH-32 were designed to test the impact of time between SIV infection and the initiation of antiretroviral drugs (ART) with respect to vaccine immunogenicity and protective potential of therapeutic vaccinations.

- The TH-12 study was designed to test the effectiveness of therapeutic vaccinations in rhesus macaques placed on an ART regimen relatively soon after infection (12 weeks).
- The TH-18 study was designed to test the effectiveness of therapeutic vaccinations in animals placed on ART at an intermediate time after infection (18 weeks).
- The TH-32 study was designed to test the effectiveness of therapeutic vaccinations in animals placed on ART late after infection at a time when the group was developing AIDS (32 weeks).
- The control group (TH-C) was placed on drugs at 10 weeks post infection.

All of the vaccination regimens incorporated two (TH-12 and TH-18) or three (TH-32) doses of the DNA vaccine and one dose of the MVA vaccine with vaccines delivered to rhesus macaques at the time when plasma virus was well suppressed by 30 to 40 weeks of ART. Treatment interruption (stopping of drugs) was initiated 6 weeks after the administration of the MVA component. Throughout the trial (infection, treatment, vaccination, and treatment interruption phases) levels of viral RNA, CD4+ T cell counts, the magnitude of anti-viral T cells and titers of antibodies specific for Env were measured. Therapeutic success was measured as the fold

reduction in viral RNA; comparison of the levels measured at the time of initiation of ART to that at 12 and 24 weeks post treatment interruption.

In addition to time between infection and ART initiation, the therapeutic studies explored a number of other conditions, none of which were found to affect efficacy (Table 1). Specifically, the studies explored the use of CD40 ligand (CD40L) (expressed in cis in the DNA prime) and imiquimod (applied at the site of inoculation as a cream) as adjuvants for the DNA prime. CD40L can provide costimulation to responding CD8+ T cells and B cells through the CD40 receptor expressed on dendritic cells and B cells (22, 23). Imiquimod stimulates dendritic cells through Toll like receptor 7(24). The trials used different timings of inoculations and both IM and intradermal (ID) routes of vaccination for the DNA prime (the ID route was used in trials that tested the imiquimod cream as a DNA adjuvant) (see Table 1). The pilot studies were conducted by Drs. Rama Rao Amara and Francois Villenger at the Yerkes National Primate Research Center of Emory University.

Table 1. Summary of therapeutic trials conducted with prototype SIV239 DNA and MVA vaccines

Study	Infection	Time After Infection to Initiation of ART (drugs used in treatment)	Number Vaccinated (experimental conditions)
TH-12	SIV239, IV.	12 weeks<br><br>(PMPA and FTC, then PMPA, FTC, AZT, Kaletra)	2 non-adjuvanted<br><br>Both DNA and MVA IM<br><br>DNA 1.5 mg at weeks 0 and 8<br>MVA $1x10^8$pfu at week 16
TH-18	SIV251, IV	18 weeks<br><br>(PMPA, FTC, AZT, Kaletra)	2 non-adjuvanted, 5 CD40L plus imiquimod adjuvanted<br><br>DNA ID, MVA IM<br><br>DNA 1.5 mg at weeks 0 and 4<br>MVA $1x10^8$ pfu at week 9
TH-32	SIV251, IR (intrarectal)	32 weeks<br><br>(PMPA, FTC, AZT, Kaletra)	2 non-adjuvanted, 2 CD40L plus imiquimod adjuvanted<br><br>DNA ID, MVA IM<br><br>DNA 1.2 mg at weeks 0, 4, and 8<br>MVA $1x10^8$ pfu at week 13

TH-C	SIV239, IV	10 weeks (PMPA)	5 non-vaccinated

The results of these pilot studies suggest that the most important factor for therapeutic success was the duration of infection prior to an animal being placed on ART (Figure 1). The two animals that were started with ART at 12 weeks post infection had a median reduction in post-treatment-interruption viral RNA of 1000-fold whereas those that started ART at 18 weeks had a median reduction of 100-fold; and those that started ART at 32 weeks had a median reduction of 10-fold. At 24 weeks post treatment interruption, the median reductions in viral RNA were overall similar to those at 12 weeks post treatment interruption (Figure 1). While the group sizes are small and it is therefore difficult to assess statistical significance, the benefit of the vaccination component of these studies is readily demonstrated by the fact that the unvaccinated controls had essentially no reductions in viral RNA post treatment interruption.

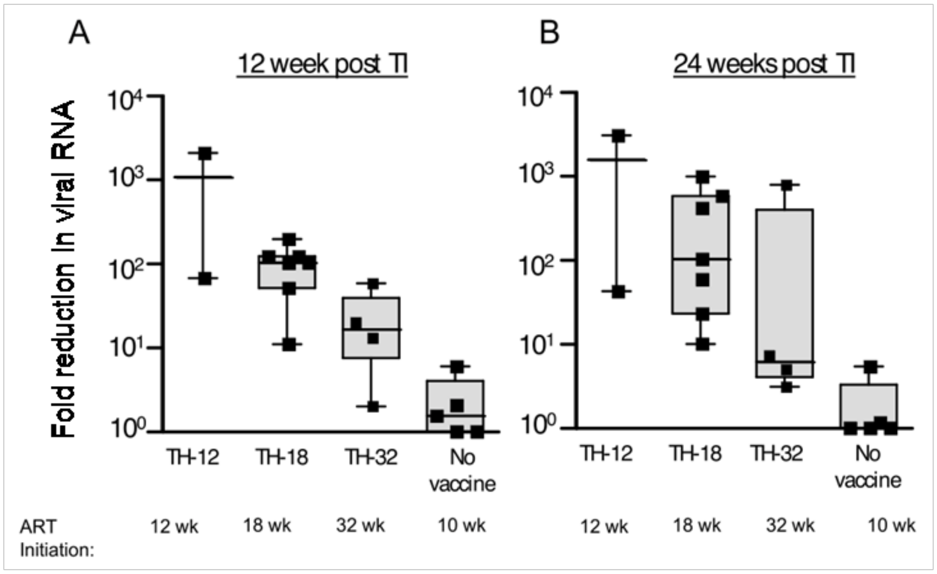

Figure 1. Logarithmic differences in levels of viral RNA at the initiation of ART and at 12 or 24 weeks post-treatment interruption for animals placed on ART at different times post infection. Fold reduction was determined by dividing the titer of viral RNA at the time of ART initiation by the titer of viral RNA at 12 (panel A) or 24 (panel B) weeks post treatment interruption (TI).

However, the spread in the fold- reductions in viral RNA was greater at 24 than 12 weeks post treatment interruption, due primarily to 40% of the vaccinated animals, the best controller in TH-12, three animals in TH-18, and one animal in TH-32, improving control between 12 and 24 weeks post treatment interruption. Between 12 and 24 weeks post treatment interruption, some animals were characterized with reduced control, a phenomenon that was most marked in the TH-32 group, which had been placed on drugs late after infection and which had achieved the most marginal control at 12 weeks post treatment interruption.

Given the move towards annual HIV-1 testing, early initiation of therapy (within 6 months of documented seroconversion) and vaccination is a feasible scenario for HIV-1 treatment that merits careful clinical as well as further preclinical evaluation.

2.3 Previous Clinical Studies with JS7 DNA and MVA62B Vaccines

The JS7 and MVA62B vaccines are being evaluated by clinical testing for preventative applications through the NIH-sponsored HIV Vaccine Trials Network (HVTN). A 120 participant Phase 1 trial (HVTN 065) has been completed (Table 2) and a 299 participant (224 vaccinees, 75 placebo recipients) Phase 2a trial (HVTN 205) in healthy, HIV-1–uninfected volunteers aged 18 to 50 years is in long-term followup (Table 3). The HVTN 065 trial included a dose escalation study (Part A, groups T1 and T2) and a dose regimen study (Part B, groups T3 and T4). Of the different regimens in HVTN 065, the high dose regimen used in Part A (group T2) and the MMM regimen in Part B (group T4) are the regimens that have been taken forward into the Phase 2a HVTN 205 study. The high dose regimen used in Part A for both JS7 DNA and MVA62B is based on the delivery of 3 mg of JS7 DNA at weeks 0 and 8 and $1x10^8$ $TCID_{50}$ of MVA62B at weeks 16 and 24. This regimen is also the one to be used for this therapeutic Phase 1 study. In the MMM regimen in Part B, subjects are vaccinated with $1x10^8$ $TCID_{50}$ of MVA62B at weeks 0, 8, and 24. All inoculations are IM. Below we report safety and immunogenicity results for HVTN 065 and the data are published (25).

Table 2. HVTN 065 Schema

					Vaccination schedule in weeks (days)			
			Dose		Prime		Boost	
Part	Group	N	JS7 DNA	MVA 62B	0 (0)	8 (56)	16 (112)	24 (168)
A	T1	10	0.3 mg	$1×10^7$ $TCID_{50}$	DNA	DNA	MVA	MVA
	P1	2	—	—	placebo	placebo	placebo	placebo
	T2	30	3 mg	$1×10^8$ $TCID_{50}$	DNA	DNA	MVA	MVA
	P2	6	—	—	placebo	placebo	placebo	placebo
B	T3	30	3 mg	$1×10^8$ $TCID_{50}$	DNA	MVA	—	MVA
	P3	6	—	—	placebo	placebo	—	placebo
	T4	30	—	$1×10^8$ $TCID_{50}$	MVA	MVA	—	MVA
	P4	6	—	—	placebo	placebo	—	placebo
	Total	100+ 20						

Table 3. Trial Schema for HVTN 205

		Dose		Vaccination Schedule in Weeks (Months)			
**Group**	**Subject Number**	**DNA (mg)**	**MVA ($TCID_{50}$)**	**0 (0)**	**8(2)**	**16 (4)**	**24 (6)**
**Part A**	**180**						
Group 1	120	3	1×10^8	DNA	DNA	MVA	MVA
Group 2	60	0	0	Placebo	Placebo	Placebo	Placebo
**Part B**	**119**						
Group 3	30	3	1×10^8	DNA	DNA	MVA	MVA
Group 4	74	0	1×10^8	MVA	MVA	Placebo	MVA
Group 5	15	0	0	Placebo	Placebo	Placebo	Placebo
**Totals:** 224 treated subjects + 75 placebo subjects = 299							

2.3.1 Safety results in HVTN 065

Local and systemic reactions related to vaccination with the JS7 DNA and MVA62B vaccines in the different treatment groups of the HVTN 065 study were mild to moderate, and overall, the safety profile was acceptable and no patterns of toxicity emerged. Immunogenicity data were generated for the different regimens. These data showed that the DDMM regimen induced the highest T cell response rates, whereas the MMM regimen elicited the highest antibody response rates. Safety and immunogenicity data are provided in the subsequent sections.

Frequency of local and systemic reactogenicity signs and symptoms following vaccination
Reactogenicity

Local reactogenicity and systemic events are summarized in Table 4. Pain, tenderness, erythema, and induration at the injection site were considered as local reactogenicity if they occurred within 72 hours of the vaccination. Systemic events included fever, malaise/ fatigue, myalgia, headache, nausea, vomiting, chills, arthralgia with onset within 72 hours following vaccination.

As summarized in Table 4, subjects in placebo and T1 reported no or mild local reactogenicity. Up to one-third of subjects in groups T2, T3, and T4 reported moderate local reactogenicity. None of the participants reported any severe local reactogenicity reactions.

With respect to maximum systemic symptoms, moderate reactions were noted in all groups, including placebo, but no more than about one-quarter of subjects in any group experienced moderate systemic reactogenicity. There were no severe systemic reactions in any group. Most subjects did not experience temperature elevation, and those who did (≤10%) experienced mild fever. There was no difference in systemic reactogenicity between the placebo and vaccine recipients.

Severe or Life Threatening Adverse Events

There were a total of eight Grade 3 or 4 adverse events in this study, as graded according to The Division of AIDS Table for Grading the Severity of Adult and Pediatric Adverse Events (DAIDS AE Grading Table), Version 1.0, December, 2004. In Part A of the study (treatment groups T1 and T2), CPK elevation was noted twice; one a Grade 4 (T1) and the other Grade 3 (T2) (Table 5). The Grade 4 event was considered probably not related to study drug, and the Grade 3 event was not considered to be related to administration of the vaccines. In Part B of the study (treatment groups T3 and T4), four Grade 3 or Grade 4 adverse events were observed. In treatment group T3, a Grade 4 cardiac arrhythmia and a Grade 3 uterine leiomyoma were observed and neither event was considered to be related to administration of the vaccines. In treatment group T4, one subject experienced a Grade 3 diarrhea and another Grade 3 Salmonellosis; neither was considered to be related to the administration of the vaccines. Two participants in the placebo group also experienced Grade 3 reactions. These included a Grade 3 QTc prolongation and a Grade 3 procedural pain; neither of which were considered to be related to the vaccines. In summary, the recorded Grade 3 or 4 adverse events were all classified as not related to vaccine administration except for the Grade 4 CPK elevation, which was considered probably not related.

Table 4. Maximum severity of reactogenicity symptoms by treatment group in Parts A and B, across all vaccinations[1]

	P1-P4 (N=20) N (%)	T1 (N=10) N (%)	T2 (N=30) N (%)	T3 (N=30) N (%)	T4 (N=30) N (%)	Total (N=120) N (%)
**Symptoms and Severity**						
**Pain**						
None	**10 (50)**	**7 (70)**	**12 (40)**	**5 (16.7)**	**5 (16.7)**	**39 (32.5)**
Mild	**10 (50)**	**3 (30)**	**11 (36.7)**	**23 (76.7)**	**15 (50)**	**62 (51.7)**
Moderate	**0**	**0**	**7 (23.3)**	**2 (6.7)**	**10 (33.3)**	**19 (15.8)**
Severe	**0**	**0**	**0**	**0**	**0**	**0**
**Tenderness**						
None	**7 (35)**	**6 (60)**	**9 (30)**	**3 (10)**	**1 (3.3)**	**26 (21.7)**
Mild	**13 (65)**	**4 (40)**	**17 (56.7)**	**21 (70)**	**19 (63.3)**	**74 (61.7)**
Moderate	**0**	**0**	**4 (13.3)**	**6 (20)**	**10 (33.3)**	**20 (16.7)**
Severe	**0**	**0**	**0**	**0**	**0**	**0**
**Erythema or Induration**						
None	**19 (95)**	**10 (100)**	**25 (83.3)**	**23 (76.7)**	**26 (86.7)**	**103 (85.8)**
>0 to 25 cm^2	**1 (5)**	**0**	**5 (16.7)**	**6 (20)**	**3 (10)**	**15 (12.5)**
>25 to 81 cm^2	**0**	**0**	**0**	**1 (3.3)**	**1 (3.7)**	**2 (1.7)**
>81 cm^2	**0**	**0**	**0**	**0**	**0**	**0**
**Maximum Sys-temic Symptoms**						
None	**9 (45)**	**6 (60)**	**13 (43.3)**	**10 (33.3)**	**11 (36.7)**	**49 (40.8)**
Mild	**6 (30)**	**2 (20)**	**10 (33.3)**	**12 (40)**	**11 (36.7)**	**41 (34.2)**
Moderate	**5 (25)**	**2 (20)**	**7 (23.3)**	**8 (26.7)**	**8 (26.7)**	**30 (25)**
Severe	**0**	**0**	**0**	**0**	**0**	**0**
**Temperature**						
None (34.0 – 37.6 ° C)	**19 (95)**	**9 (90)**	**27 (90)**	**28 (93.3)**	**28 (93.3)**	**111 (92.5)**
37.7 - 38.6 ° C Grade 1	**1 (5)**	**1 (10)**	**3 (10)**	**2 (6.7)**	**2 (6.7)**	**9 (7.5)**
38.7 – 39.3° C Grade 2	**0**	**0**	**0**	**0**	**0**	**0**

1 Symptoms are considered as local reactogenicity if the onset date was within 72 hours following vaccination.

Table 5. Grade 3 (severe) or Grade 4 (life-threatening) adverse events (AEs) in placebo, Part A, and Part B treatment groups (N=120)

Group	Adverse Event	Relationship to Vaccine
Placebo (P1-P4)	Grade 3 QT_c prolongation	Not related
	Grade 3 Procedural pain	Not related
Part A		
Treatment group 1	Grade 4 CPK elevation	Probably not related
Treatment group 2	Grade 3 CPK elevation	Not related
Part B		
Treatment group 3	Grade 4 cardiac arrhythmia	Not related
	Grade 3 uterine leiomyoma	Not related
Treatment group 4	Grade 3 diarrhea	Not related
	Grade 3 Salmonellosis	Not related
Total: 8 events	Six Grade 3 and two Grade 4 adverse events	

Possibly, Probably or Definitely Related Adverse Events

Twenty-one adverse events that were deemed possibly, probably or definitely related to the administration of the vaccines occurred on study in 20 subjects. Of the 17 adverse events that occurred in the treatment groups, lymphadenopathy (4 cases) and axillary pain (4 cases) were the most common. Only two events were considered definitely related to vaccine administration:

- one case of axillary pain in T4, and
- one case of lymphadenopathy in T3.

Additional cases of axillary pain (3 episodes) and lymphadenopathy (3 cases) were classified as either possibly (2 episodes of axillary pain in T2 and 1 case each of lymphadenopathy in T2 and T3) or probably (1 case each of axillary pain (T3) and lymphadenopathy (T4)) related to vaccine administration. Three adverse events that were considered probably related to study drug included:

- one episode of injection site pruritis in a subject in T4,
- a case of decreased neutrophil count (moderate or Grade 2) in a subject in T2 14 days after the first vaccination, and
- one case of dyspnea in a T2 subject.

Events classified as possibly related to the administration of the vaccines included:

- sinus congestion (2 episodes, one in T3 and one in T4),
- prolonged QT interval (2 cases in T1),
- decreased hemoglobin (1 episode in T4), and
- oropharyngeal pain (1 case in T4).

Treatment group T1 (low dose vaccines, consisting of 0.3 mg DNA and 10^7 $TCID_{50}$ MVA62B) was characterized by the fewest number of adverse events. Adverse events in the other groups were fairly equally dispersed (placebo: 4 events; T2: 5 events; T3: 4 events; T4: 6 events), although adverse events in patients receiving vaccines which were considered definitely related to treatment were observed only in groups T3 (one DNA vaccine, followed by 2 MVA vaccines) and T4 (3 MVA vaccines). Four of these events occurred on placebo (prolonged QT interval, injection site pruritus, decreased neutrophil count, dizziness) which were all possibly related except injection site pruritis, which was considered definitely related.

Discontinuation status: The numbers of subjects per group that completed each vaccination and the reasons for early discontinuation of vaccination are listed in Table 6.

Table 6. Parts A and B: Vaccination completion and reasons for early discontinuation of vaccination

Group	Vaccination schedule in weeks (days) and subjects completing each vaccination				Reasons for early discontinuation of vaccinations occurring in each group
	0 (0)	8 (56)	16 (112)	24 (168)	
P1-P4<br>N=20	20	20	8 (100%)<br>Gps 1 & 2 only	19 (95%)	Pregnancy
T1<br>N=10	10	10	9 (90%)	9 (90%)	Unable to contact subject
T2<br>N=30	30	30	27 (90%)	26 (87%)	Unable to contact subject (3);<br>Participant refused (1)
T3<br>N=30	30	29 (97%)	0<br>Gps 1 & 2 only	26 (87%)	Pregnancy (1); Adverse event (1); Unable to contact (1); Other (1)
T4<br>N=30	30	26 (87%)	0<br>Gps 1 & 2 only	24 (80%)	Adverse event (1); Unable to contact (2); Refused (2); Other medical (1)
Total<br>N=120	120	115 (96%)	44 (92%)<br>Gps 1 & 2 only	104 (87%)	

HVTN 065 Safety Summary

In summary, local and systemic reactions related to vaccination with the JS7 DNA and MVA62B vaccines in Parts A and B of the HVTN 065 study were mild to moderate. Axillary pain and lymphadenopathy were the most common adverse events considered to be related to the vaccines, with four cases of each occurring during the study. However, no pattern of systemic adverse events emerged during the study, and no SAEs related to the study vaccines were observed (one Grade 4 CPK elevation was probably not related). The vaccines were well-tolerated, with an acceptable safety profile in this trial.

2.3.2 Immunogenicity results for HVTN 065

Immunogenicity studies for HVTN 065 were conducted at the HVTN central laboratories and at GeoVax Labs. Response rates are reported in Figures 2 and 3.

2.3.2.1 HVTN 065 Immunogenicity Summary of T Cell Responses

The low and high dose DDMM regimens induced the highest number of T cell responders, higher than the DMM or MMM regimens (Figures 2A, 2B). Responses were not detected following the DNA priming immunizations. However, following the MVA62B booster immunizations, subjects on the full dose DDMM regimen achieved a maximal CD4+ response rate of 77%, compared to response rates of 87% for the low dose DDMM and 43% for the MMM regimen. MVA also boosted maximal response rates for CD8+ T cells, with full dose and low dose DDMM regimens exhibiting response rates of 42% and 33%, respectively, compared to a 17% response rate in the MMM arm. Subjects who received DDMM achieved maximal CD8+ T cell response rates after the final vaccination, compared to the MMM arm, in which maximal T cell response rates occurred after the second of the three MVA62B vaccinations. A single DNA priming dose was not as effective as two DNA priming doses for eliciting maximal T cell response rates. Response rates to Gag were more frequent than those to Env.

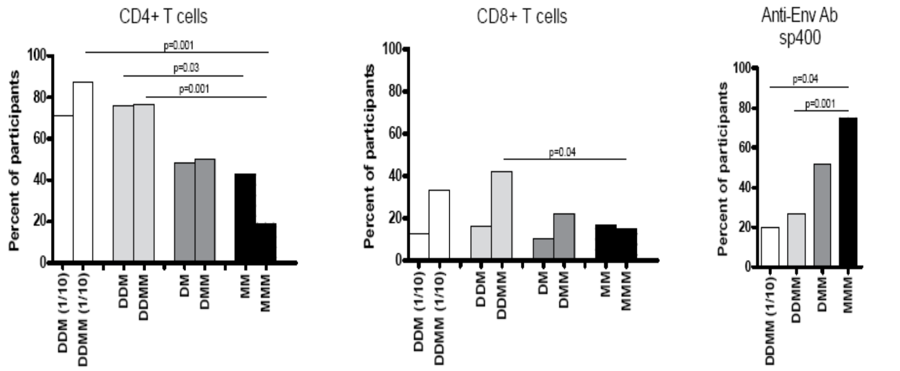

Figure 2. Immune response rates determined in HVTN end point assays. Response rates for CD4+T cells (A), CD8+T cells (B) and Anti-Env Ab (C). Responses for CD4+and CD8+T cells are for responses to Gag, Env, or Pol measured as IFN-γ or IL-2 producing cells scored using intracellular cytokine staining (ICS) following stimulation with "potential T cell epitope" (PTE) peptide pools. Response rate for anti-Env Ab were measured using an ELISA for the sp400 peptide, a peptide representing the immunodominant region of gp41. Lymphocytes and sera for determining response rates were harvested at 2 weeks following immunizations. All assays were performed in HVTN laboratories on frozen samples. Letters at the bottom of schematics indicate group, and the immunization status of groups.

2.3.2.2 HVTN 065 Immunogenicity Summary of Antibody Responses

The MMM regimen induced the highest antibody titers and rates of antibody responses to the HIV-1 Env protein (Figures 2C., 3A., and 3B.). Whereas 27% of subjects vaccinated with DDMM had detectable levels of anti-Env (gp41) antibody at 2 weeks following the last immunization, 75% of MMM treated individuals had detectable antibody (Figure 2C.). Antibody responses were determined using a validated ELISA assay and a TZM-bl cell line-based neutralization assay (Figure 3).

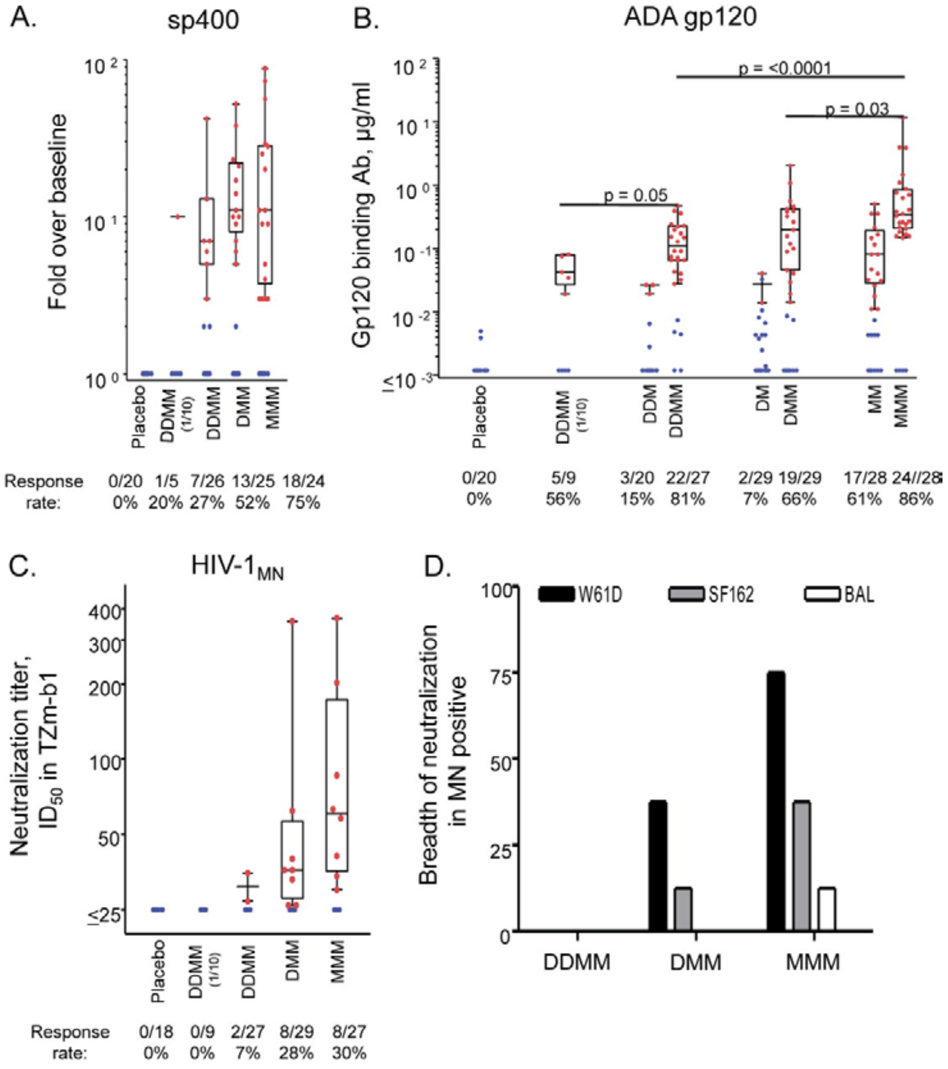

Figure 3. Magnitudes and response rates of Env binding and neutralizing antibodies. *A*, Binding Ab for SP400, a peptide representing the immunodominant region of gp41. *B*, Binding Ab for ADA gp120. *C*, Neutralizing Ab for HIV-1_{MN}. Designations below schematics indicate groups and response rates. The box plots show median and 25th and 75th percentiles for positive data (indicated by red points); blue points indicate negative data. Data for determining *P* values include only positive data. *D*, Percent of positive MN neutralization responses also neutralizing other tier 1 isolates. Seventeen of the samples demonstrating neutralization against HIV-1_{MN} were evaluated further. The tier 1 isolates are shown including HIV-1_{SF162}, HIV-1_{W61D} (T cell laboratory-adapted strain), and HIV-1_{BAL}.

2.3.3 Introduction to HVTN 205

HVTN 205 is a Phase 2a multicenter, randomized, controlled, double-blind trial conducted at 11 clinical sites in the US and two sites in Peru. Two hundred ninety-nine healthy, HIV-1 uninfected volunteers, aged 18 to 50 years, were enrolled (150 DDMM vaccine; 74 MMM vaccine; and 75 placebo recipients) (Table 3). The primary objective was to extend the assessment of the safety and tolerability of a heterologous prime boost regimen of JS7 DNA and

MVA62B vaccines (DDMM regimen) and of a homologous prime boost regimen of MVA62B alone (MMM regimen). A secondary objective was to extend the assessment of the immunogenicity of the two regimens in healthy HIV-uninfected, vaccinia-naïve individuals; specifically, the CD4+ and CD8+ T cell response rates and magnitude and functional profiles of responding CD4+ and CD8+ T cells and to evaluate the frequency and titer of humoral immune responses to Env and Gag.

In HVTN 205, participants received either two doses of JS7 DNA (3 mg) at weeks 0 and 8 followed by two doses of MVA62B ($1x10^8$ $TCID_{50}$) at weeks 16 and 24, three doses of MVA62B at weeks 0, 8, and 24, or placebo. Placebo for both DNA and MVA vaccines was sodium chloride for Injection USP, 0.9%. All vaccines were administered by IM injection with needle and syringe into the deltoid muscle. The estimated total study duration of subjects on HVTN 205 is 65 months, which includes enrollment, follow-up, and annual health contacts. HVTN 205 is currently in long-term followup. The trial schema for HVTN 205 is presented in Table 3.

2.3.4 HVTN 205 Safety

The safety profiles for the DDMM and MMM regimens have expected patterns based on those observed in HVTN 065.

2.3.4.1 Reactogenicity

Local and systemic reactogenicity are summarized in Figure 4. Pain and/or tenderness, erythema and/or induration at the injection site are considered local reactogenicity if they occurred within 72 hours of the inoculation. Systemic events included fever, malaise/fatigue, myalgia, headache, nausea, vomiting, chills, and arthralgia with onset within 72 hours following vaccination. Participants self-reported the maximum severity of events.

In all three groups (DDMM, MMM, and placebo), the majority of subjects experienced no or mild local and systemic reactogenicity.

Of the 150 total subjects treated with DDMM, almost 40% experienced moderate local reactogenicity in the form of pain and/or tenderness. Moderate local symptoms were noted by 40% of participants after vaccinations 3 and 4, compared to 2% of participants after vaccinations 1 and 2, indicating that the MVA62B vaccinations were somewhat more reactogenic than the JS7 DNA vaccinations in this group. There was one local severe event of pain and tenderness that occurred after the first MVA62B vaccination. Three subjects experienced moderate (Grade 2) erythema and/or induration, defined as >81 cm^2 or >9 cm any diameter.

About one-fourth of subjects (27%) on the DDMM arm experienced moderate systemic symptoms, and two subjects had severe malaise and/or fatigue (one occurred after the second JS7 DNA vaccination; the other occurred after the first MVA62B injection).

Of the 74 participants in the MMM group, the incidence (about 34%) of moderate local reactogenicity (pain and/or tenderness) was similar to that occurring after the two MVA62B vaccinations in the DDMM group (31%). There was one severe event of tenderness and one event of moderate erythema.

Approximately 15% of subjects in the MMM group had moderate systemic symptoms. One subject had severe chills and fatigue/malaise after the second MVA62B vaccination. One participant had a severe event of fever following the third MVA62B vaccination.

In the placebo group (n=75), substantially less moderate pain and/or tenderness (about 3%) occurred compared to the DDMM and MMM groups. No severe pain and/or tenderness events occurred. No moderate or severe erythema and/or induration reactogenicity events occurred. There were fewer moderate maximum systemic symptoms (11%) in the placebo group than the two treatment groups. No severe systemic reactogenicity events occurred in the placebo group.

2.3.4.2 Adverse Events

2.3.4.2.1 Grade 3, Serious, or Life-Threatening Adverse Events

There were no serious (SAE) adverse events reported on this study that were related to vaccine. Nineteen participants (12 DDMM, 3 MMM, 4 placebo,) experienced 31 severe or potentially life-threatening adverse events. Only one event was considered related to vaccination. One young female subject experienced a severe (Grade 3) allergic reaction after her second MVA62B vaccin**a**tion (Part B of the study – MMM regimen) that was considered definitely related to the MVA62B inoculations. The patient felt fine and vital signs were normal prior to the injection. The allergic reaction began within 15 minutes of injection and was characterized first by a small area of redness at the injection site that expanded and progressed up the subject's neck. The participant complained of dizziness, chills/feeling cold, and nausea. The subject never experienced shortness of breath, wheezing, or angioedema. The subject was treated with oral and IM Benadryl and epinephrine (Epipen). The participant was observed in the research clinic. Symptoms improved with treatment and the redness began to resolve within 2 hours after vaccination. The subject did not receive further vaccinations, but continued with follow up visits. This reaction was not considered an SAE and thus was not reported in a safety report to FDA.

2.3.4.2.2 Mild and Moderate Adverse Events Related to Vaccination

A total of 24 participants (10 DDMM, 9 MMM, and 5 placebo) experienced mild (Grade 1) or moderate (Grade 2) AEs that were considered probably or definitely related to vaccination on the three treatment arms. Seventeen probably or definitely related events occurred in 10 participants on the DDMM arm, the arm with the most adverse events. Of these events, 2 were moderate and 15 were mild. The moderate events were vaccination site pain and axillary pain. The mild events

were injection site pain (3); injection site hematoma (3); lymphadenopathy (2); nasal congestion (2); injection/vaccination site induration (1); axillary pain (1); injection site paresthesia (1); vaccination complication (lightheadedness, 1); and decreased lymphocytes (1).

Thirteen probably or definitely related mild or moderate adverse events occurred in 9 participants on the MMM arm. Of these events, one was moderate (injection site pain). The 12 mild events were injection/vaccination site pain (3); lymphadenopathy (3); injection site hematoma (1); injection site pruritis (1); injection/ vaccination site induration (1); muscle swelling (1); injection site swelling (1); and vaccination complication (warmth at injection site, 1).

The placebo arm had the fewest adverse events, with 6 mild or moderate events considered probably or definitely related to vaccination. One person had a moderate AE of rash. The mild events were injection site pain (2 separate events in 1 participant); injection site pruritis (1); dizziness (1); and injection site hematoma (1).

In summary, there was one severe allergic reaction that was definitely related and that resolved on the same day. Injection/vaccination site pain, lymphadenopathy, and injection site hematoma were the most common AEs considered probably or definitely related to vaccination. Lymphadenopathy was not reported among placebo recipients. Alanine aminotransferase increase was another frequent AE, reported in 7 participants (2.7% DDMM, 2.7% MMM, and 1.3% of placebo subjects), and considered possibly related to vaccination. Subjects receiving placebo inoculations experienced at least 50% fewer adverse events overall compared to those in the treatment groups. However, the JS7 and MVA62B vaccines were very well-tolerated from a safety perspective in the 224 subjects (150 DDMM and 74 MMM subjects) who received them.

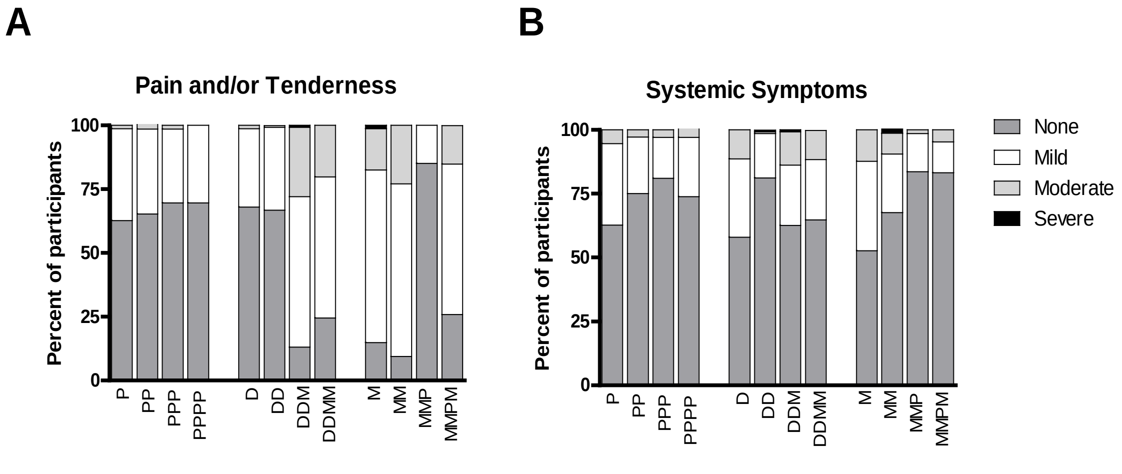

Figure 4. Local Pain and/or Tenderness (A) and Maximum Systemic Reactogenicity (B) in HVTN 205 (Unblinded Data)

2.3.5 Immunogenicity of Study Products in HVTN 205

2.3.5.1 Response Rates for Ab and T Cell Responses

2.3.5.1.1 Introduction to HVTN 205 Antibody and T Cell Responses

Both the DDMM and MMM regimens in HVTN 205 elicited T cell and Ab responses (Figure 5). In agreement with the results in HVTN 065, the DDMM regimen elicited the highest T cell responses whereas the MMM regimen elicited the highest Ab responses (Figure 5). As in HVTN 065, T cell responses to Gag were seen at a greater frequency than to Env and were very infrequent for Pol (Figure 6). The higher T cell response rate to Gag versus Env was significant for CD4+ responses elicited by both DDMM and MMM regimens but not for the CD8+ T cell responses. Ab responses were higher for gp41 than gp120 for both regimens. The MMM group had higher response rates than the DDMM group for gp120-specific Ab as well as neutralizing Ab for tier 1 and tier 2 viruses.

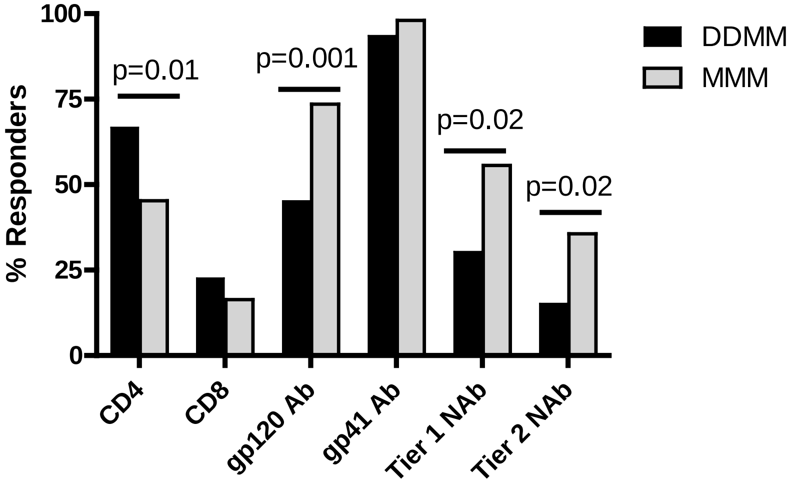

Figure 5. Response rates for Vaccine elicited T cells and Ab in HVTN 205. All assays were conducted by the HVTN central laboratory. T cells were scored by ICS. Ab was scored in ELISA assays for the indicated substrates. Tier 1 neutralizing Ab was determined using the TZMbl assay and Tier 2 neutralizing Ab using the A3R5 assay. CD4, CD4+ T cells, CD8, CD8+ T cells, NAb, neutralizing Ab

2.3.5.1.2 T Cell Responses

Subjects on the DDMM regimen achieved a 66.4% response rate for CD4+ T cells and a 21.8% response rate for CD8+ T cells, as compared with response rates in the MMM group of 43.1% for CD4+ T cells and 14.9% for CD8+ T cells (Figure 6). Also, as in HVTN 065, median magnitudes for CD4+ responses were 0.1-0.2% of total CD4+ T cells (Figure 7) and 0.2-0.4% of total CD8+ T cells (Figure 8).

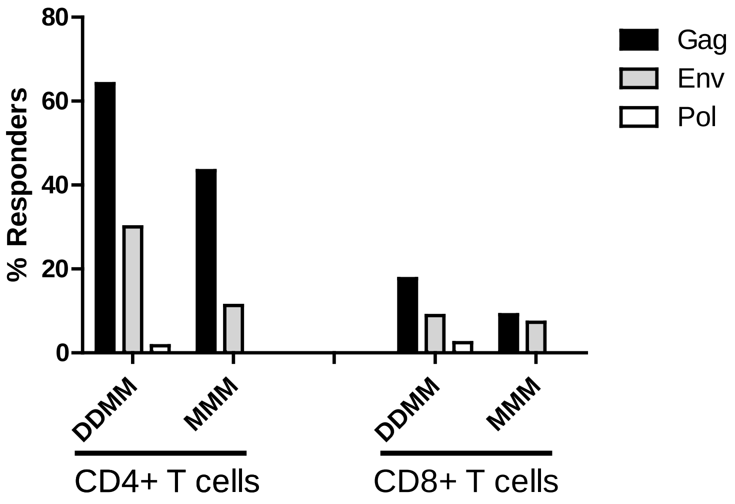

Figure 6. Specificity of Elicited T cell responses. Response rates were determined at the HVTN Laboratory at the Fred Hutchinson Cancer Research Center using ICS.

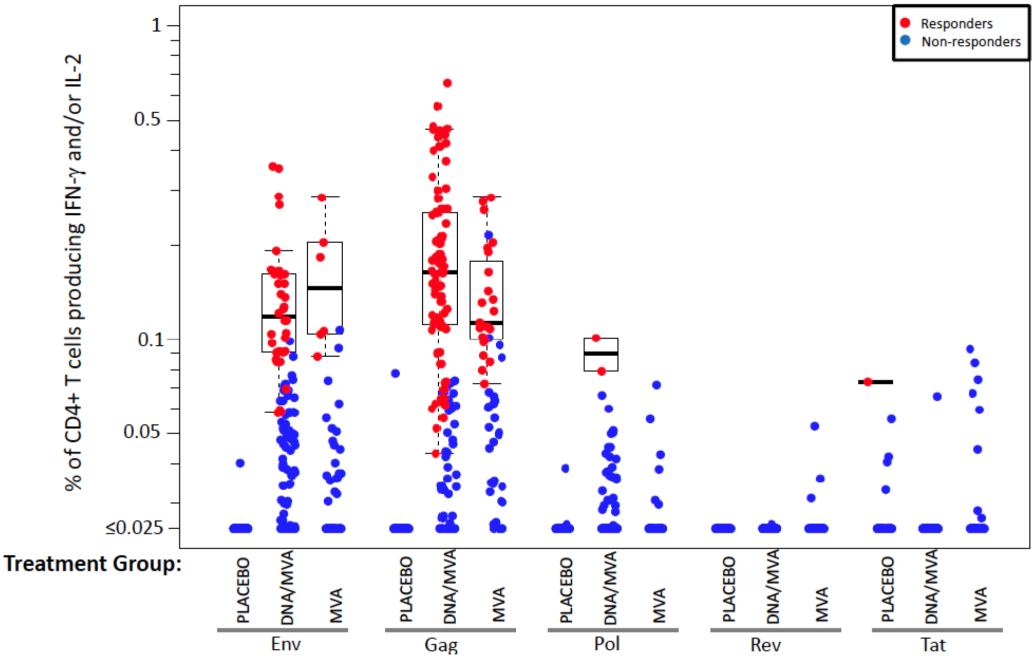

Figure 7. Magnitudes of CD4+ T cell responses. Boxplots represent the median, 25Th and 75th percentiles for the magnitudes of positive responses. Red dots, responders; Blue dots, non-responders.

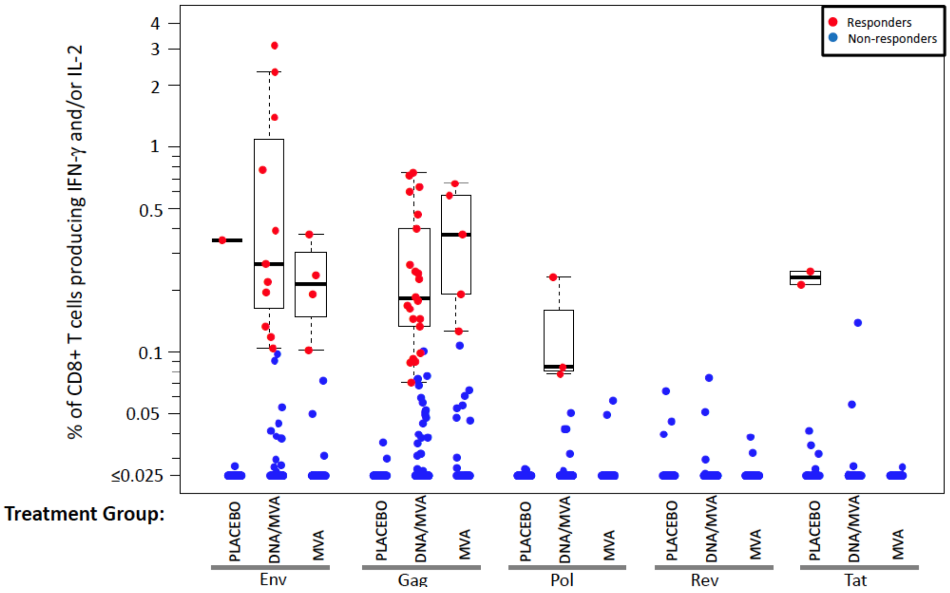

Figure 8. Magnitudes of CD8+ T cell responses. Boxplots represent the median, 25[Th] and 75th percentiles for the magnitudes of positive responses. Red dots, responders; Blue dots, non-responders.

2.3.5.1.3 Antibody Responses

The titers of Ab responses, measured as Mean Fluorescence Intensities (MFI) at the HVTN Duke Laboratory, are summarized in Figure 9. In HVTN 205, Ab responses were higher for gp41 than gp120 for both regimens with 95.3% of the MMM and 92.5% of the DDMM inoculated participants responding with Ab for gp41. Seventy percent of the MMM group had binding Ab for gp120 compared with 47% of the DDMM group. 64.4% percent of the MMM group had neutralizing Ab for tier 1 and 37% had neutralizing Ab for tier 2 isolates, compared to 30% and 15% in the DDMM group, respectively. The higher response rates in the MMM vaccinated group were also associated with higher titer IgG responses. The highest titers were for gp41, followed by gp140 and then gp120.

Assays testing for the presence of monomeric IgA responses in serum were also conducted (Figure 10A). IgA response rates were much lower than IgG response rates, with the MFI for IgG to IgA having a median of 19 for the DDMM group, and 40 for the MMM group (Figure 10B). The presence of Env-specific monomeric IgA is an important consideration for vaccine trials because monomeric Env-specific IgA appeared to compete with protective ADCC activities in RV144, the first HIV vaccine trial to achieve some success.

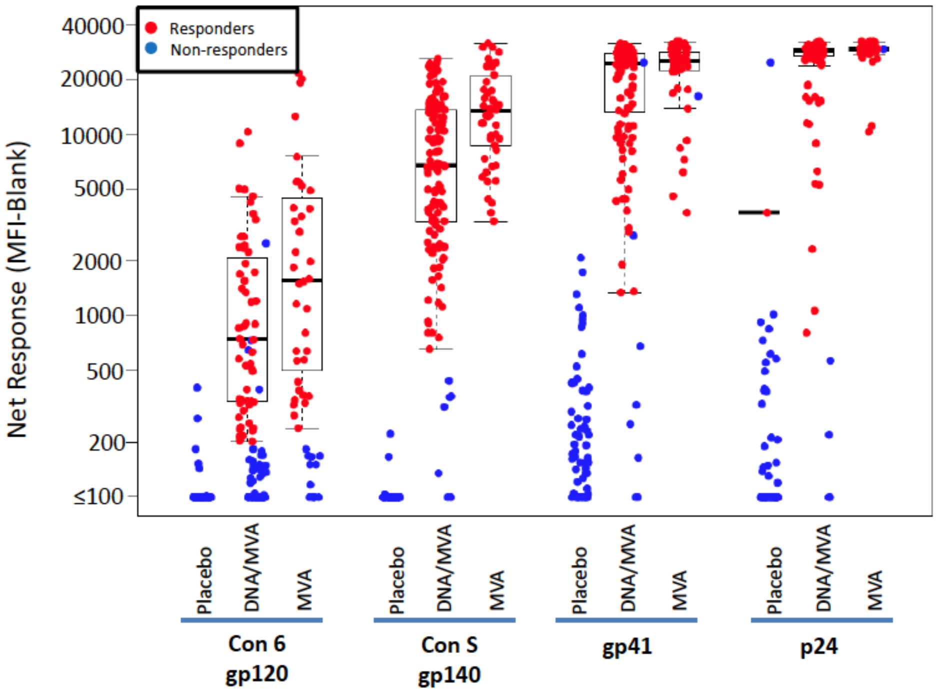

Figure 9. Ab responses to Env and Gag. Assays were conducted at the HVTN central laboratory at Duke University. The antigens used for the different assays are indicated for the X axis.

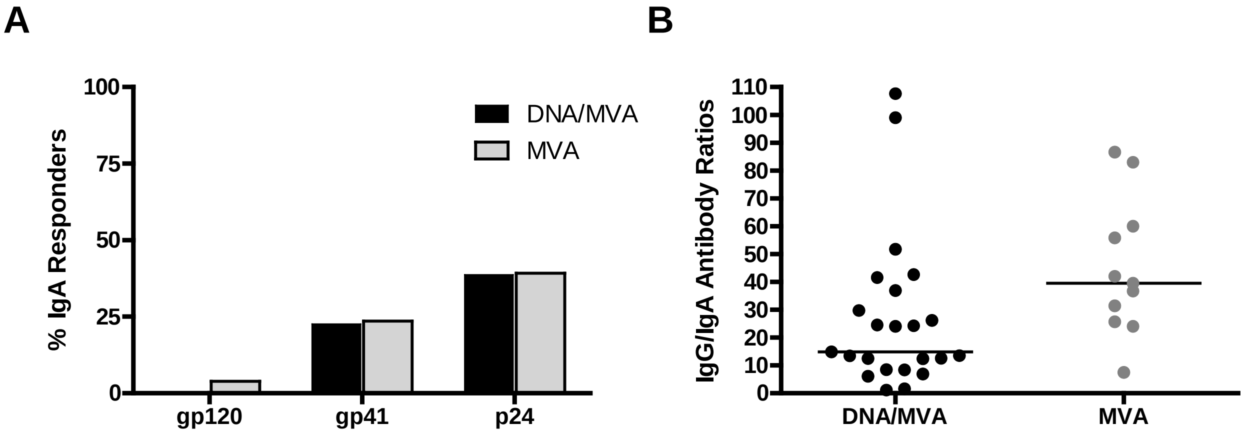

Figure 10. Vaccine-elicited IgA responses in serum. A. % IgA responders. B. Ratio of IgG to IgA response. The ratio of responses was tested for gp41 Env. Tests were conducted at the HVTN Duke Central Laboratory.

Neutralizing Ab was tested for both tier 1 (easy to neutralize) and tier 2 (more difficult to neutralize) isolates of HIV-1 (Figure 11). Consistent with the higher response rates for neutralizing Ab in the MMM group (Figure 5), the MMM group also had higher breadth of neutralizing Ab responses (Figure 11) and higher titers of neutralizing Ab (Figure 12).

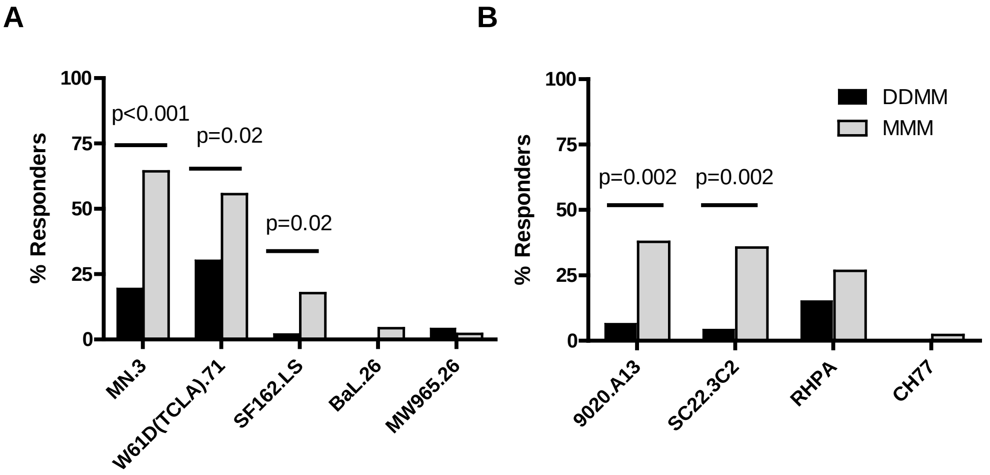

Figure 11. Breadth of neutralizing Ab responses elicited in DDMM and MMM vaccine groups. A. Breadth for tier 1 viruses measured in the TZMbl assay. B. Breadth for Tier 2 isolates, measured on A3R5 cells. All assays were conducted at the HVTN Duke Laboratory.

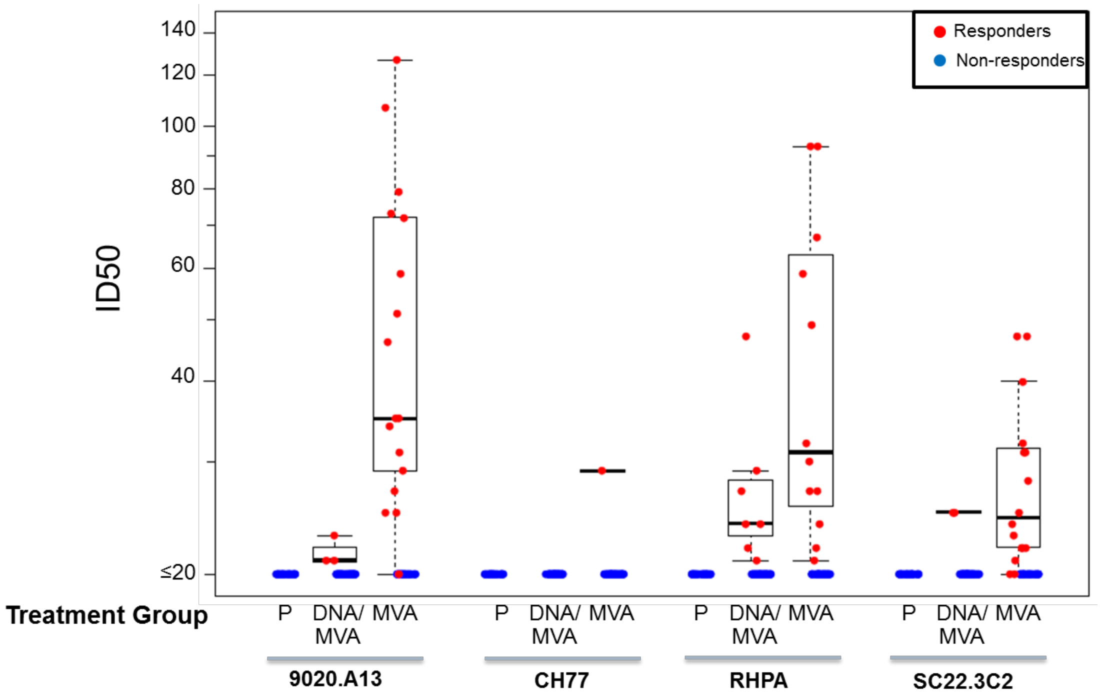

Figure 12. Titer of Ab responses to Tier 2 isolates. The trial groups and test isolates are indicated on the X axis. Neutralization assays were conducted in A3R5 cells at the Duke HVTN lab. Red dots, responders; blue dots, non-responders.

2.3.5.2 Summary of Prior Preclinical and Immune Response Data

The development of a therapeutic HIV-1 vaccine is the subject of this clinical protocol. Preclinical pilot studies completed using SIV-infected macaques treated with antiretroviral drugs and then vaccinated were used to demonstrate control of re-emergent virus post treatment interruption. The control of viral replication was observed to be correlated inversely with the

time infected prior to initiation of ART; suggesting that therapeutic vaccination will be most successful in infected individuals who have initiated ART relatively soon after infection.

In the HVTN 065 Phase 1 clinical trial, the administration of two full doses of the JS7 DNA vaccine followed by two doses of the MVA62B vaccine was completed with an excellent safety profile. No evidence of MVA replication was noted in any subject. Analyses for vaccine-induced T cells and antibodies documented that 77% of the individuals produced CD4+ T cell responses and 88% produced antibody responses to Env. CD8+ T cells were elicited in 42% of the participants receiving the full dose of vaccine. Both CD4+ and CD8+ T cell responses were balanced between Gag and Env. Both types of T cell responses were characterized with favorable multicytokine production profiles.

The DDMM and MMM vaccine groups in HVTN 205 showed similar patterns of immune responses as observed in HVTN 065. The DDMM regimen elicited the highest T cell responses, whereas the MMM regimen elicited the highest Ab responses. CD4+ T cell responses were observed in 66.4% of the DDMM group and 43.1% of the MMM group. CD8+ T cell response rates in these two groups were 21.8% and 14.9% respectively. In both groups, response rates for Env-specific Ab were highest for gp41, second highest for gp140, and lowest for gp120 Env. Ninety-three percent of the DDMM participants and 95.3% of the MMM participants responded with Ab for gp41, whereas 47% of the DDMM and 70% of the MMM participants responded with Ab for gp120. Neutralizing Ab responses rates and the titers of neutralizing Ab were higher for the MMM than the DDMM group with 64.4% of the MMM group having neutralizing responses for tier 1 viruses and 37% having responses for tier 2 isolates. This compared with a 30% response rate for neutralizing Ab for tier 1 viruses and a 15% response rate for neutralizing Ab for tier 2 isolates in the DDMM group. IgA responses in serum were low with the ratio of IgG to IgA responses being higher for the MMM (P=0.03) than the DDMM group.

Overall the patterns of responses were similar to those observed in preclinical studies of the DDMM and MMM regimens. Interestingly in the preclinical studies, the different patterns of immune responses did not correlate with different extents of protection against repeat rectal challenges with SIVE660. In challenge studies with the DDMM and the GM-CSF-adjuvanted DgDgMM regimens, prevention of infection has correlated with the avidity of the Env-specific Ab response for the Env of the E660 challenge virus. In studies using the MMM regimen, avidity has not correlated with prevention of infection. GeoVax's hypothesis is that the full length gp160 Env expressed by the DNA in the DDMM and DgDgMM regimens primes for a more protective Ab response than the gp150 Env expressed by both the prime and boost in the MMM regimen and that the Ab elicited by the DNA-primed regimens is more effective at providing non-neutralizing Ab mediated protection than the Ab primed and boosted by MVA.

Thus, there are numerous advantages to the vaccine approach to be tested in this therapeutic Phase 1 clinical trial. Both the DNA and MVA constructs express the three major structural HIV-1 genes (*gag, pol, and env*) which encode viral proteins that are well known targets of the immune system. These vaccine products have proven safe and immunogenic in humans in Phase 1 and Phase 2 clinical studies in uninfected participants conducted by the HVTN. HVTN 065 is completed and HVTN 205 is in long-term followup.

2.4 Rationale for the Current Trial Design

This protocol is designed to assess the safety and immunogenicity of a prime-boost vaccine regimen of JS7 DNA and MVA62B in HIV-infected persons who initiated a successful ART regimen within 18 months of a negative HIV-1 antibody test. The study includes a period of ART interruption during which intensive safety, virologic and immunologic monitoring will be performed. The monitoring of CD4+ cell count, HIV-1 RNA viral load and clinical condition will guide the decision to resume ART prior to the end of the 12 week analytic treatment interruption period. At the conclusion of the analytic treatment interruption phase, ART will be resumed. HIV-1 drug resistance testing will be completed during the analytic treatment interruption phase, at the time of rebound viremia, and subsequently if indicated following treatment reinstitution. Successful completion of this Phase 1 clinical trial will provide information about:

- the safety of vaccination in HIV-1 infected persons,
- the immunogenicity of the vaccine products, and
- the ability of this vaccine regimen to induce immune responses that impact viral load following ART discontinuation.

Should the results be encouraging, the data from this trial will be used to plan and power a placebo-controlled trial to further assess the antiviral activity of this therapeutic vaccine approach.

2.4.1 Rationale for Study Population Selection

At the time of primary infection with HIV, the virus induces specific antibody and cellular immune responses. Studies of "elite HIV-1 controllers" (defined as those who are infected with HIV-1 but maintain HIV-1 RNA viral loads below 50 copies/mL without antiretroviral therapy) indicate the importance of high levels of HIV-1 specific cellular immunity, including polyfunctional HIV-specific CD4+ T cells and a vigorous HIV-specific CD8+ T cell response (34). Unfortunately, in the overwhelming number of HIV-1 infected individuals, immune responses are unsuccessful in eliminating or controlling infection in the longer term. The goal of therapeutic vaccination is to induce effective levels of cellular immune responses to supplement ART.

In the setting of acute HIV-1 infection, HIV-specific immune responses may contribute to at least partial control of viremia (35, 36). Treatment in the setting of acute infection may sustain or strengthen HIV-specific immune responses (7, 37, 38). However, when therapy is withdrawn, viremia rebounds indicating a lack of control of viral replication (39, 40). As infection persists uncontrolled, HIV-1 specific immune responses are lost and continuing viral escape from the remaining immune responses occurs in the majority of patients. After this point, immune reconstitution through ART does not restore HIV-1 specific immune responses and viral rebound occurs whenever therapy is withdrawn (41).

Throughout HIV-1 infection, the virus undergoes immune escape from both CD8+ and CD4+ T cell responses. Recent sequence data show that CD8+ T cell responses rapidly select escape mutants that first appear concurrent with falling virus loads in acute infections (42, 43). Early T cell responses to the virus wane as escape occurs. New T cell responses to epitopes that escape more slowly or are invariant then will often be induced. With time, an infecting HIV can escape essentially the entire CD8+ repertoire of its host. Initiation of ART within 18 months of a documented negative HIV-1 antibody test is an inclusion criterion for entry into this therapeutic protocol. The purpose of this inclusion criterion is to provide a means of delivering vaccines before the virus has accumulated mutations that render it resistant to killing by CD8+ T cells.

Therefore, the setting of acute or recent HIV-1 infection followed by suppressive ART may be an ideal situation in which to intervene with therapeutic vaccination. If vaccination is successful in strengthening HIV-specific immune responses, the potential for eliminating or diminishing viral rebound might be realized. Ultimately, if therapeutic vaccination can contribute to viral control in the absence of ART, persons with HIV-1 could be spared the cumulative toxicity and cost of lifelong ART, the risk of selection of drug resistant virus, the exhaustion of therapeutic options and the ultimate progression of HIV-1 disease.

2.4.2 Rationale for Design of Vaccination Phase

The selected dose and vaccine regimen for this protocol are based on the safety and immunogenicity findings obtained as the result of the successful completion of the HVTN 065 Phase 1 trial conducted in healthy uninfected adults (see section 2.3). In HVTN 065 both the one-tenth (0.3 mg JS7 DNA and $1x10^7$ $TCID_{50}$ MVA62B) and full-dose (3 mg JS7 DNA and 1x 10^8 $TCID_{50}$ MVA62B) vaccine regimens were well tolerated and without any significant safety concerns (Table 2). The frequency of vaccine-induced CD4+ T-cell and CD8+ T-cell responses were also observed to be similar using these two dose levels (Figure 2). However, Env-specific antibody responses were increased in frequency in participants receiving the full dose of the DNA vaccine (Figure 2). The second MVA booster immunization appears to be required to induce higher frequencies of Gag-specific CD8+ T cells in the prophylactic setting (Figure 2) and to enhance the anti-Env antibody responses (Figure 3). Thus, the regimen containing two doses of DNA followed by two doses of the MVA vaccine will be used in this Phase 1 trial. Finally, IM administration was selected since this route of vaccine administration is well

accepted and was shown to deliver both the DNA and MVA vaccines in a safe and immunogenic manner in both preclinical and clinical studies.

To increase the safety of the vaccination phase, enrollment will be staggered, so that two subjects will receive both DNA vaccines and one MVA vaccine first, with a gap of at least 2 weeks before the first dose of MVA is given in the remaining subjects.

2.4.3 Rationale for Design of the Analytic Treatment Interruption Phase Including Discontinuation of Efavirenz

Effective immune responses are unlikely to be induced by vaccine administration in the presence of high-level HIV-1 replication. This requires a study design wherein HIV-1 replication is suppressed using ART and vaccine is administered while HIV-1 replication is largely undetectable. Although ART interruption is not recommended as part of routine care, the anti-viral activity of a therapeutic vaccine can be best evaluated in the setting that allows for HIV-1 replication. Thus, a short term (12 week) interruption of ART is planned to enable the assessment of the safety and immunogenicity of this approach, herein referred to as “analytic treatment interruption”. Because an effective therapeutic HIV-1 vaccine would have tremendous benefit to people living with HIV-1, this protocol balances the need for a short period of analytic treatment interruption with multiple safeguards to protect the health of trial participants. The analytic treatment interruption phase of our trial has been carefully designed to maximize patient safety.

A period of 12 weeks for ART interruption was selected as a time period that is sufficiently limited to avoid increased likelihood of progression to AIDS or death as well as increased occurrence of non-AIDS defining clinical events such as cardiovascular, renal, or hepatic diseases and malignancies. Events such as these only occurred with much longer analytic treatment interruptions in the SMART study, a large study testing the effects of analytic treatment interruption (44). The analytic treatment interruption period of only 12 weeks is also shorter than the 16-week analytic treatment interruption in the recent therapeutic vaccine trial AACTG AA5197 (see clinical trials.gov, NCT00080106). In AA5197, 107 of 110 participants completed the 16-week analytic treatment interruption. There were only two adverse events judged to be possibly or probably related to treatment (headache and thrombocytopenia). Thus, the data from AA5197 support a 12-week period of analytic treatment interruption as sufficient to assess effects of therapeutic vaccination on rebound virus without serious safety concerns (45).

To further protect trial participants, more stringent thresholds were selected for early reinstitution of ART. During the period of analytic treatment interruption, (Section 7.4.3), ART will be re-initiated if HIV-1 RNA exceeds 300,000 copies/mL on two consecutive visits, or if CD4+ counts drop below 350 cells/µL on two consecutive measurements at least two weeks apart. Both of these criteria are more stringent than in AA5197 where treatment was reinstituted only if the

viral load exceeded 300,000 copies/mL on three consecutive visits and if the CD4+ count dropped below 300 cells/µL on two consecutive visits. Also, in the SMART study, where there were adverse consequences for prolonged treatment interruption, therapy was reinstituted at a CD4+ count of 250 cells/µL. In SMART, the amount of time spent with a CD4+ cell count below 250 cells/µL was associated with increased risk of clinical events (44, 46). Here we propose to reinitiate drugs at CD4+ counts of 350 cells/µL, consistent with the current standard of care for initiation of therapy (46).

Although not advised, if a patient declines to reinstitute therapy at the end of 12 weeks of treatment interruption due to prolonged viral suppression, and the patient's primary care physician and the Principal Investigator agree, the sponsor may allow the patient to continue in the study in an extension of the treatment interruption phase. Under these circumstances, the patient will continue the same schedule throughout treatment interruption and may enter the reinstitution of therapy phase when the decision is made to restart treatment. The length of continuation of follow up in the treatment interruption phase will be at the sponsor's discretion.

To decrease the chance for selection of drug resistant mutants during analytic treatment interruption, great care will be taken to avoid "functional monotherapy" due to one drug in the regimen having a significantly longer half-life than the others. Of the commonly used agents for initial therapy, efavirenz is the most problematic in this regard. Persons carrying the cytochrome P450 2B6 G516T mutation have prolonged and variable efavirenz clearance that may result in the maintenance of therapeutic plasma levels for over 3 weeks following efavirenz discontinuation. Due to the low genetic barrier to resistance of efavirenz, the risk of selecting resistant HIV-1 is high during efavirenz monotherapy (47). Because efavirenz is one of the leading agents for initial therapy of HIV-1 in the US, the protocol includes additional safety precautions for efavirenz discontinuation.

Specifically, at the end of the vaccination phase, patients on an efavirenz-containing regimen will substitute a boosted protease inhibitor or integrase inhibitor (raltegravir) for efavirenz and other drugs will continue for a minimum of two weeks. Efavirenz serum levels will be monitored regularly to determine the timing of analytic treatment interruption in an effort to assure that there is no remaining efavirenz drug pressure when ART is finally discontinued.

2.4.4 Rationale for Design of the Treatment Reinstitution Phase

Following the analytic treatment interruption phase, ART will be reinstituted and all patients will be followed for an additional 24 weeks. The purpose of this phase is to ensure that participants can safely and effectively resume therapy. For all patients, the virus that re-emerges during the analytic treatment interruption phase will be genotyped to determine if a drug resistance variant has been selected. As long as genotypic resistance data does not contraindicate the use of the pre-vaccination ART regimen, patients will restart their pre-vaccination ART regimen at the time of drug reinstitution. If the genotypic resistance data does contraindicate use of the pre-

vaccination ART regimen, an appropriate regimen will be instituted in consultation with the study investigator and the patient’s primary care provider.

3. STUDY DESIGN

This trial will assess the safety and immunogenicity of a DNA/MVA vaccine in individuals who began highly suppressive ART treatment within 18 months of a negative HIV-1 antibody test and have stably controlled viral replication to a level of less than 50 copies/mL for at least 6 months.

3.1 Vaccination Phase

The study will include an immunization regimen conducted in the presence of continued antiretroviral therapy. The immunization regimen includes two doses of the JS7 DNA vaccine at week 1 and week 9 to prime the immune responses followed by two doses of the MVA62B vaccine at weeks 17 and 25 to boost immune responses. This phase of the study will be used to evaluate the safety of the vaccine regimen and to measure vaccine immunogenicity in the target population. After the first two patients receive their first MVA62B vaccines, 2 weeks must elapse before subsequent patients may receive their first MVA62B vaccine. This staggered approach will allow monitoring of the first MVA62B recipients before exposing additional patients to MVA62B.

3.2 Analytic Treatment Interruption Phase

The analytic treatment interruption phase of the study will be used to evaluate both safety and immunogenicity and provide a basis to assess the association between vaccine-induced immune responses and control of viral rebound in the absence of ART. To assure the safety of discontinuing an efavirenz-containing regimen, patients will switch efavirenz to a boosted protease inhibitor (BPI) or raltegravir, an integrase inhibitor, while continuing the other drugs in the regimen. Efavirenz serum levels will be monitored until the entire ART regimen can be safely discontinued (see Section 7.4.2). Viral load and CD4+ thresholds are in place to trigger early reinstitution of therapy if needed to protect patient safety. ART will be re-initiated if HIV-1 RNA exceeds 300,000 copies/mL on two consecutive visits, or if CD4+ counts drop below 350 cells/µL on two consecutive measurements at least 2 weeks apart.

3.3 Treatment Reinstitution Phase

The 24-week treatment re-initiation phase will be used to evaluate safety and durability of immune responses during the period of resumption of highly suppressive ART, including assessment of viral resistance and immune response parameters should virus not be rapidly re-suppressed. If rebound viremia occurs following viral suppression, or if viral suppression does not occur, resistance testing will guide the patient and primary care provider in the choice of subsequent therapies (see Section 2.4.4). Figure 13 depicts the three study phases and their timing.

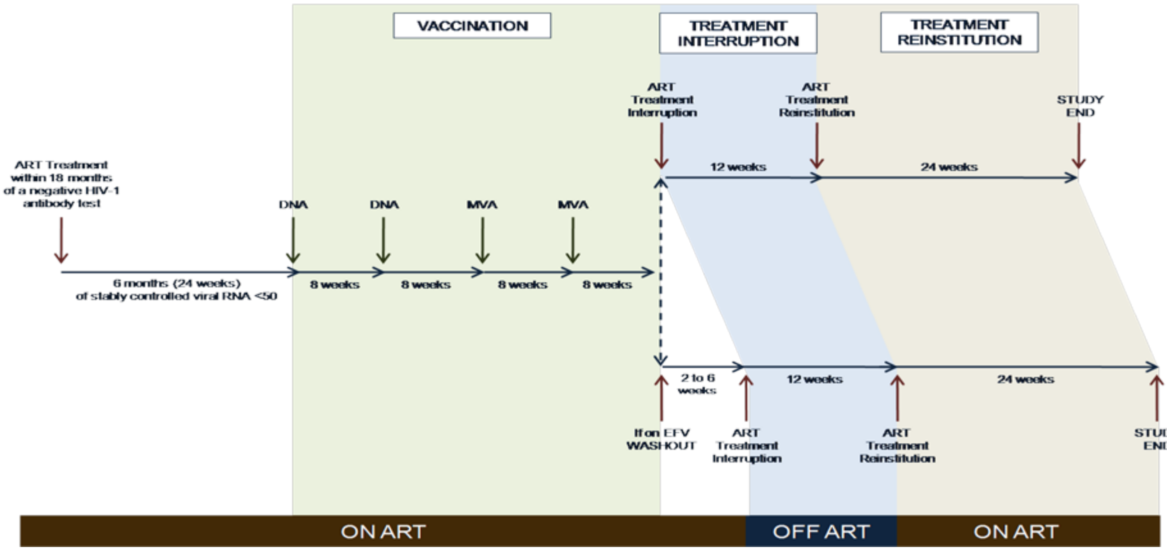

Figure 13. Schematic for therapeutic trial showing study phases

4. STUDY OBJECTIVES

4.1 Primary and Secondary Objectives

Primary Objective

- To evaluate the safety of the therapeutic use of JS7 DNA and MVA62B vaccines during vaccination, analytic treatment interruption and treatment reinstitution

Secondary Objectives

- To evaluate immunogenicity of JS7 DNA and MVA62B vaccines during the vaccination phase of the trial
- To evaluate HIV-1 RNA levels and CD4+ T cell counts during analytic treatment interruption
- To evaluate HIV-specific immune responses during the analytic treatment interruption phase of the trial

4.2 Exploratory Objectives

- To explore possible correlations between peak post vaccination immune responses and the control of re-emergent virus during analytic treatment interruption
- To explore possible correlations between control of re-emergent virus during analytic treatment interruption with levels of markers for inflammation during the period of analytic treatment interruption
- To explore levels of HIV-1 RNA prior to the initiation of ART and levels in the analytic treatment interruption phase of the trial

5. STUDY POPULATION

5.1 Inclusion Criteria

1. Age 18 to 50 years
2. Meets one or more of the following criteria:
   a. Time between last documented negative HIV antibody and initiation of antiretroviral therapy ≤ 18 months.; OR
   b. Time from negative detuned HIV-1 assay to initiation of ART is ≤ 13 months; OR
   c. Time between evolution of Western blot from indeterminate to positive (as defined by CDC/ASTPHLD)(48) in the presence of a positive HIV antibody and initiation of ART is ≤ 18 months.
3. No changes to ART treatment within 4 weeks of study entry
4. Documentation of plasma HIV-1 RNA and CD4+ counts prior to beginning ART
5. On stable suppressive ART, defined as HIV-1 RNA < 50 copies/mL (PCR) or < 75 copies/mL (bDNA) for a minimum of 6 months prior to starting vaccination:
   - At least two measures must have occurred within the 6 months preceding vaccination (One may be from the chart and one at screening)
6. No history of virologic failure, defined as:
   - HIV-1 RNA > 50 copies/mL (PCR) or > 75 copies/mL (bDNA) after achieving suppression below those levels
   - Failure to reach HIV-1 RNA < 50 copies/mL (PCR) or < 75 copies/mL (bDNA) on initial antiretroviral regimen. (Substitutions due to intolerance are allowed.)
7. CD4+ > 500 cells/µL
8. Nadir CD4+ > 350 cells/µL unless measured in the setting of acute infection
9. Laboratory values:
   - Hemoglobin ≥ 10g/dL (male) or 9g/dL (women)
   - ANC > 1000 cells/µL
   - ALT, AST ≤ 2.5 ULN
   - Total bilirubin < 1.5 x ULN (≤ 5 x ULN on atazanavir)
   - Fasting glucose ≤ 125 mg/dL
   - Serum creatinine < 1.5 x ULN
   - Creatinine clearance ≥ 60 mL/min (Cockcroft – Gault)
   - Serum creatine phosphokinase (CPK) ≤ 1.5 x ULN
   - UA negative for hemoglobin and glucose and no greater than 1+ protein
   - Any abnormalities must be assessed by the investigator as not clinically significant.
10. ECG without evidence of current or past MI, or ischemic heart disease
11. Willing to provide signed informed consent
12. Female subjects must have a negative serum β-HCG pregnancy test at screening
13. Female subjects of reproductive potential (not having reached menopause or not having undergone hysterectomy, bilateral oophorectomy, or tubal ligation) who engage in sexual

activity that could lead to pregnancy must agree to avoid pregnancy and agree to consistently use contraception for at least 21 days prior to first vaccination until the last protocol visit. Contraception is defined as using at least one of the following methods:

- Condoms (male or female)
- Diaphragm or cervical cap
- Intrauterine device (IUD)
- Hormonal contraceptive with one of the following: condoms, diaphragm or cervical cap

14. Male subjects participating in the study must agree to not attempt to impregnate a female, or participate in sperm donation programs
15. Males engaging in sexual activity that could lead to pregnancy must use a condom from the date of receipt of the first study vaccine until the last protocol visit
16. Male and female subjects participating in the study must agree to use condoms for protection against HIV-1 transmission throughout the study
17. Participants must be willing to comply with all study requirements and expected to be available for the duration of the study
18. Participants must be willing to temporarily discontinue antiretroviral therapy for up to 12 weeks post-vaccinations

5.2 Exclusion Criteria

1. Known infection with HIV-1 subtype other than clade B
2. Receipt of chemotherapy for active malignancy in the past 12 months
3. Prior vaccinations with any HIV-1 vaccine
4. Prior vaccination against smallpox within the last 15 years
5. History of or known cardiac disease including:
    - myocardial infarction
    - angina pectoris
    - congestive heart failure
    - cardiomyopathy
    - pericarditis
    - stroke or transient ischemic attack
    - symptoms suggestive of cardiac disease in the opinion of the investigator
6. History of myositis
7. Diagnosis of HIV-associated nephropathy
8. Evidence of active hepatitis B or C infection, including positive HBsAg or positive hepatitis C antibody
9. Framingham Global Risk Assessment Score consistent with high short-term (10 year) cardiac risk (Appendix E)

10. Receipt of immunomodulatory agents, systemic corticosteroids (including nonprescription street steroids), gamma globulin, or investigational agents (other than H1N1 influenza vaccine) within 6 months of screening
11. Any immunization within 1 month of screening and within 2 weeks of any inoculation in this study.
12. Creatine supplements within 14 days of baseline and unwillingness to discontinue use throughout the trial
13. Changes in ART regimen prior to entry due to virologic failure (not including toxicity)
14. Pregnancy or breastfeeding
15. Any clinically significant diseases (other than HIV-1 infection) or clinically significant findings during the screening medical history or physical examination that, in the investigator's opinion, would compromise participant safety
16. Active alcohol or substance abuse, defined as use that is likely to interfere with the ability of the subject to adhere to medications, return for study visits or lead to adverse events
17. Allergy to chicken egg derived products
18. Contraindication to intramuscular injection such as anticoagulant therapy or thrombocytopenia
19. Unwilling to forego vigorous exercise 3 days prior to each vaccination

6. STUDY PRODUCT PREPARATION AND ADMINISTRATION

6.1 Study Product Formulation

6.1.1 pGA2/JS7 DNA

The JS7 DNA vaccine to be used in this study is Lot FIN-0591, which is supplied as an opalescent to clear, colorless solution in single-dose vials containing a volume sufficient for the delivery of 1 mL containing approximately 3 mg DNA. The DNA is formulated in a buffer consisting of PBS, EDTA and ethanol. The vials containing study product must be stored frozen at −70 °C (−94 °F) or colder.

6.1.2 MVA/HIV62B

The MVA62B vaccine lots to be used in this study are Lots 1309.07 and 1309.08. The product is supplied as an opalescent to off-white suspension, that may contain fine particles, in single-dose vials containing a volume sufficient for the delivery of 1 mL containing approximately 1×10^{8} $TCID_{50.}$ The vaccine is formulated at a titer of $1\times10^{8.0\pm0.5}$ $TCID_{50}$ in a buffer consisting of PBS and 7.5% sucrose. The vials containing study product must be stored frozen at −70 °C (−94 °F) or colder.

6.2 Preparation, Administration, and Dispensing of Study Products

6.2.1 Preparation of pGA2/JS7 DNA

One vial of pGA2/JS7 DNA (labeled as 3 mg/mL), will be needed to prepare each dose. Prior to injection, the pharmacist will remove the 3 mg DNA/mL vial of vaccine from the freezer and allow it to thaw in a 2 - 8 °C refrigerator for 2 - 18 hours. During this 2 - 18 hour period, if the vaccine is not completely thawed and a subject is ready to be vaccinated, the pharmacist may hold the vial in his/her hand to complete the thawing process. The thawed vaccine can be held at 2- 8 °C for up to 5 days (120 hours) after removal from the freezer. DO NOT STORE AT ROOM TEMPERATURE.

Immediately prior to withdrawing a 1 mL dose into a 3 or 5 mL syringe, the vial should be removed from the refrigerator and inverted gently several times. DO NOT SHAKE. Using aseptic technique, the pharmacist should withdraw the entire contents of the vial, up to 1 mL, into a 3 or 5 mL syringe. The syringe containing the product must not be held at room temperature. Once drawn into the syringe, the vaccine should be kept cold (2 – 8 °C) until it is ready to be injected into the participant. The contents of the syringe should be administered within 24 hours after preparation. The syringe should be labeled as ***JS7 vaccine 3 mg DNA.***

Any unused portion of opened vials and expired filled syringes will be disposed of as a biohazard.

6.2.2 Preparation of MVA/HIV62B $1{\times}10^8$ $TCID_{50}$

One vial of MVA62B (labeled as MVA62B $1{\times}10^{8\pm0.5}$ $TCID_{50}$/mL) will be needed to prepare each dose. Prior to injection, the pharmacist will remove the vial from the freezer and allow it to thaw in a 2 - 8 °C refrigerator for 2 - 18 hours. During this 2 - 18 hour period, if the vaccine is not completely thawed and a subject is ready to be vaccinated, the pharmacist may hold the vial in his/her hand to complete the thawing process. The thawed MVA62B vaccine can be held at 2 - 8° C for up to 5 days (120 hours) after removal from the freezer. DO NOT STORE AT ROOM TEMPERATURE.

Immediately prior to withdrawing a 1 mL dose into a 3 or 5 mL syringe, the vial should be removed from the refrigerator and inverted gently several times. DO NOT SHAKE. Using aseptic technique, the pharmacist will withdraw the entire contents of the vial, up to 1 mL, into a 3 or 5 mL syringe. Once drawn up into the syringe, the vaccine should be kept cold (2 - 8 °C) until use. The contents of the syringe should be administered within 24 hours after preparation. The syringe should be labeled as ***MVA/HIV62B vaccine*** $\boldsymbol{1x10^8}$ $\boldsymbol{TCID_{50}}$***.***

Any unused portion of entered vials and expired filled syringes should be disposed of as a biohazard.

6.3 Administration

Prior to administration, the study product in the syringe should be removed from the refrigerator. It may be warmed to room temperature or warmer by holding in one's hand for several minutes at the participant's side immediately prior to administration.

The pGA2/JS7 DNA and the MVA/HIV62B vaccinations are to be given IM into the deltoid muscle after preparation of the site with alcohol.

When preparing a dose in a syringe and administering the dose, attention should be given to conserving the full dose of product.

- JS7 DNA vaccine 3 mg will be administered as the contents of one vial (up to1 mL) IM in either deltoid at weeks 1 and 9.
- MVA62B vaccine containing approximately $1x10^8$ $TCID_{50}$ will be administered as the contents of one vial (up to1 mL) IM in either deltoid at weeks 17 and 25.

6.4 Dispensing of Boosted Protease Inhibitor (BPI) Regimen for Patients on Efavirenz

Patients on an efavirenz-containing regimen will be required to switch from efavirenz to a ritonavir-boosted protease inhibitor or integrase inhibitor (raltegravir) at week 31 of the vaccination phase. The preferred BPI regimen is ritonavir 100 mg daily with atazanavir 300 mg daily. If necessary, other BPI options will be considered after discussion with the Medical Monitor. Raltegravir is given at a dose of 400 mg bid. The sponsor will provide the BPI regimen or raltegravir and drugs will be dispensed by the site pharmacy. Patients who are on two nucleosides will continue these drugs without change. The sponsor will not provide these drugs. Patients who are on Atripla™ will switch to BPI or raltegravir and Truvada™. Truvada™ will be obtained in the same way the patient usually obtains his/her medications.

7. CLINICAL ASSESSMENTS

7.1 The Schedule of Clinical Procedures (Appendix A)

7.2 Informed Consent

Informed consent will be obtained by site staff in accordance with Good Clinical Practices (GCP) and site Standard Operating Procedures (SOP). Informed consent will be obtained before any study procedures are initiated, and appropriate documentation of such will be included in the study source documents.

7.3 Patient Screening

7.3.1 Screening Visit

The screening visit will occur within 21 days of the baseline visit. During the screening visit, the purpose and procedures of the study will be fully explained to the participants. Those wishing to enroll in the study will sign a written informed consent form prior to any study-related clinical procedures and evaluations.

The following procedures will be performed at the screening visit:

- Review and sign the informed consent form
- Obtain medical history, vaccination history, demographic data
- Medication history
- Assessment of inclusion and exclusion criteria (with Framingham Assessment)
- Review adherence to ART
- Perform a complete physical exam (including measurement of height and weight)
- Perform a resting 12-Lead ECG
- Obtain vital signs (pulse, blood pressure, temperature) and concomitant meds
- Obtain fasting laboratory samples at screening for the following:
    - Hematology (complete blood count including platelets) and chemistry (serum electrolytes, serum creatinine, creatinine clearance, total bilirubin, BUN, glucose, AST, ALT, CPK)
    - Fasting lipid profile
    - HBsAg, HCV antibody
    - Urine for microscopic analysis
    - Serum pregnancy test (β-HCG) on females of childbearing potential
    - CD4+ cell count
    - Lymphocyte immunophenotyping for CD3/4, CD3/8, CD19, CD8/CD38/DR, CD4/CD38/DR
    - Plasma HIV-1 RNA

7.4 Enrollment and Study Visits

After subject eligibility is verified, the enrollment process will occur. A unique patient identification number will be assigned to the patient. Study vaccines will be dispensed to the study clinician as per standard pharmacy procedures.

7.4.1 Vaccination Phase

Study Day 1: (Week 1) Baseline Visit, Immunization 1

The Baseline visit must occur no fewer than 7 days or more than 21 days from the screening visit. Participants should remain at the site for at least 1 hour following the initial immunization.

The following procedures are to be performed at the Baseline visit:

- Review of the inclusion and exclusion criteria
- Medication adherence history
- Perform a symptom-driven physical exam (including vital signs and weight)
- 12 Lead ECG
- Review of signs and symptoms and concomitant medications
- Reactogenicity assessment
- Obtain laboratory samples for the following:
    - Chemistry and hematology
    - Urinalysis (dipstick)
    - Urine pregnancy test (β-HCG) for females of childbearing potential
    - CD4+ cell count
    - Lymphocyte immunophenotyping
    - PBMC (stored specimen)
    - Serum for antibody analysis (stored specimen)
    - Plasma HIV-1 RNA
    - Plasma for genotyopic resistance (stored specimen)
    - IL-6, d-Dimer, and hsCRP (stored specimens)
- Study immunization 1 administration (JS7 DNA vaccine)
- Record adverse events

Study Day 4

- Telephone call for safety and reactogenicity assessment

Study Day 8 (Week 2)

- Medication adherence history
- Perform symptom-driven physical exam (including weight measurement)
- Obtain vital signs and review concomitant medications
- Record adverse events
- Reactogenicity assessment
- Obtain laboratory samples for the following:
    - Plasma HIV-1 RNA
    - Plasma for genotypic resistance (stored specimen)

Study Days 15 and 43 (Weeks 3 and 7)

- Medication adherence history
- Perform a symptom-driven physical exam (including weight measurement)
- Obtain vital signs and concomitant medications
- Record adverse events

- Obtain laboratory samples for the following:
    - Chemistry and hematology
    - Urinalysis (dipstick)
    - CD4+ cell count
    - Plasma HIV-1 RNA
    - Plasma for genotypic resistance (stored specimen)

Study Day 57: (Week 9) Immunization 2

- Medication adherence history
- Perform a symptom-driven physical exam (including weight measurement)
- Obtain vital signs and review concomitant medications
- Record adverse events
- Reactogenicity assessment
- Obtain laboratory samples for the following:
    - Chemistry and hematology
    - Urinalysis (dipstick)
    - Urine pregnancy test (β-HCG) on females of childbearing potential
    - CD4+ cell count
    - Lymphocyte immunophenotyping
    - Plasma HIV-1 RNA
    - Plasma for genotypic resistance (stored specimen)
- Study immunization 2 administration (JS7 DNA vaccine)

Study Day 60

- Telephone call for safety and reactogenicity assessment

Study Day 64 (Week 10)

- Medication adherence history
- Perform a symptom-driven physical exam (including weight measurement)
- Obtain vital signs and review concomitant medications
- Record adverse events
- Reactogenicity assessment
- Obtain laboratory samples for the following:
    - Plasma HIV-1 RNA
    - Plasma for genotypic resistance (stored specimen)

Study Day 71 (Week 11)

- Medication adherence history
- Perform a symptom-driven physical exam (including weight measurement)

- Obtain vital signs and review concomitant medications
- Record adverse events
- Obtain laboratory samples for the following:
  - Chemistry and hematology
  - Urinalysis (dipstick)
  - CD4+ cell count
  - Lymphocyte immunophentoyping
  - PBMC (stored specimen)
  - Serum for antibody analysis (stored specimen)
  - Plasma HIV-1 RNA
  - Plasma for genotypic resistance (stored specimen)

Study Day 99 (Week 15)

- Medication adherence history
- Perform a symptom-driven physical exam (including weight measurement)
- Obtain vital signs and review concomitant medications
- Record adverse events
- Obtain laboratory samples for the following:
  - Chemistry and hematology
  - Urinalysis (dipstick)
  - CD4+ cell count
  - Plasma HIV-1 RNA
  - Plasma for genotypic resistance (stored specimen)

Study Day 113: (Week 17) Immunization 3

- Medication adherence history
- Perform a symptom-driven physical exam (including weight measurement)
- Obtain vital signs and review concomitant medications
- Record adverse events
- Reactogenicity assessment
- Obtain laboratory samples for the following:
  - Chemistry and hematology
  - Urinalysis (dipstick)
  - Urine pregnancy test (β-HCG) on females of childbearing potential
  - CD4+ cell count
  - Lymphocyte immunophenotyping
  - Plasma HIV-1 RNA
  - Plasma for genotypic resistance (stored specimen)
- Study immunization 3 administration (MVA62B vaccine)

Study Day 116

- Telephone call for safety and reactogenicity assessment

Study Day 120 (Week 18)

- Medication adherence history
- Perform a symptom-driven physical exam (including weight measurement)
- Obtain vital signs and review concomitant medications
- Record adverse events
- Reactogenicity assessment
- Obtain laboratory samples for the following:
    - Lymphocyte immunophenotyping
    - PBMC (stored specimen)
    - Plasma HIV-1 RNA
    - Plasma for genotypic resistance (stored specimen)

Study Day 127 (Week 19)

- Medication adherence history
- Perform a symptom-driven physical exam (including weight measurement)
- Obtain vital signs and review concomitant medications
- Record adverse events
- Obtain laboratory samples for the following:
    - Chemistry and hematology
    - Urinalysis (dipstick)
    - CD4+ cell count
    - Serum for antibody analysis (stored specimen)
    - Plasma HIV-1 RNA
    - Plasma for genotypic resistance (stored specimen)

Study Day 155 (Week 23)

- Medication adherence history
- Perform a symptom-driven physical exam (including weight measurement)
- Obtain vital signs and review concomitant medications
- Record adverse events
- Obtain laboratory samples for the following:
    - Chemistry and hematology
    - Urinalysis (dipstick)
    - CD4+ cell count
    - Plasma HIV-1 RNA

- Plasma for genotypic resistance (stored specimen)

Study Day 169: (Week 25) Immunization 4

- Medication adherence history
- Perform a symptom-driven physical exam (including weight measurement)
- Obtain vital signs and review concomitant medications
- Record adverse events
- Reactogenicity assessment
- Obtain laboratory samples for the following:
  - Chemistry and hematology
  - Urinalysis (dipstick)
  - Urine pregnancy test (β-HCG) on females of childbearing potential
  - CD4+ cell count
  - Lymphocyte immunophenotyping
  - Plasma HIV-1 RNA
  - Plasma for genotypic resistance (stored specimen)
- Study immunization 4 administration (MVA62B vaccine)

Study Day 172

- Telephone call for safety and reactogenicity assessment

Study Day 176 (Week 26)

- Medication adherence history
- Perform a symptom-driven physical exam (including weight measurement)
- Obtain vital signs and review concomitant medications
- Record adverse events
- Reactogenicity assessment
- Obtain laboratory samples for the following:
  - Lymphocyte immunophenotyping
  - PBMC (stored specimen)
  - Plasma HIV-1 RNA
  - Plasma for genotypic resistance (stored specimen)

Study Day 183 (Week 27)

- Medication adherence history
- Perform a symptom-driven physical exam (including weight measurement)
- Obtain vital signs and review concomitant medications
- Record adverse events
- Obtain laboratory samples for the following:

    - Chemistry and hematology
    - Urinalysis (dipstick)
    - CD4+ cell count
    - Serum for antibody analysis (stored specimen)
    - Plasma HIV-1 RNA
    - Plasma for genotypic resistance (stored specimen)

Study Day 211 (Week 31)

- Medication adherence history
- Perform a symptom-driven physical exam (including weight measurement)
- Obtain vital signs and review concomitant medications
- Record adverse events
- Obtain laboratory samples for the following:
    - Chemistry and hematology
    - Fasting lipid profile
    - Urinalysis (dipstick)
    - CD4+ cell count
    - Plasma HIV-1 RNA
    - Plasma for genotypic resistance (stored specimen)
- Patients on efavirenz will switch to boosted protease inhibitor (BPI) regimen or raltegravir on this day.

Vaccination Phase Early Termination Visit (VET)

For patients who terminate the vaccination phase prematurely, a Vaccination Phase Early Termination Visit (VET) will be performed.

- Medication adherence history
- Perform a symptom-driven physical exam (including weight measurement)
- 12-Lead ECG
- Obtain vital signs and review concomitant medications
- Record adverse events
- Obtain laboratory samples for the following:
    - Chemistry and hematology
    - Urinalysis (dipstick)
    - Urine pregnancy test (β-HCG) on females of childbearing potential
    - CD4+ cell count
    - Lymphocyte immunophenotyping
    - PBMC for responding T-cells for HIV-1 insert, and MVA vector (stored specimen)
    - Serum for antibody analysis (stored specimen)

- Plasma HIV-1 RNA
- Plasma for genotypic resistance (stored specimen)
- IL-6, d-Dimer, hsCRP (stored specimens)

7.4.2 Discontinuation of Antiretroviral Therapy for Those on Efavirenz: Schedule of Study Visits

A BPI regimen or raltegravir (integrase inhibitor) will be substituted for efavirenz at week 31. This is the beginning of the efavirenz washout period. Efavirenz washout visits (W1, W2, and W3) will occur every 2 weeks until the efavirenz serum concentration is below the limit of quantification. ART discontinuation will occur on the next visit after the visit at which the serum efavirenz concentration was below the limit of quantification. Because of pharmacogenetic differences among patients, it is anticipated that patients will reach this threshold between 2 and 8 weeks (visits 1-3) after efavirenz is discontinued. However, if additional visits are required, they will follow the procedure schedule for washout visits 1, 2, and 3. Therefore, discontinuation of the ART regimen may occur at any time after week 33. The following assessments will be performed during efavirenz washout visits.

Washout visits 1 and 2 (weeks 33 and 35)

- Medication adherence history
- Obtain vital signs and review concomitant medications
- Record adverse events
- Obtain laboratory sample for the following
    - CD4+ cell count
    - Plasma HIV-1 RNA
    - Efavirenz serum concentration

Washout visit 3 (week 37)

- Medication adherence history
- Obtain vital signs and review concomitant medications
- Record adverse events
- Obtain laboratory sample for the following:
    - Chemistry and hematology
    - Urinalysis (dipstick)
    - CD4+ cell count
    - Plasma HIV-1 RNA
    - Efavirenz serum concentration

7.4.3 Analytic Treatment Interruption Phase: Schedule of Study Visits

Patients not on efavirenz will discontinue ART 2 weeks after study week 31, also called Analytic Treatment Interruption Visit 1 (TI-1). For patients on efavirenz, TI-1 will begin after the last efavirenz washout visit. The visit procedures for the efavirenz washout period are

detailed in Section 7.4.2. Once patients have discontinued treatment, patients will follow the same analytic treatment interruption visit schedule whether they were on efavirenz or not.

Analytic Treatment Interruption Visit 1 (TI-1); Study Day TI-1; TI Week 1

On the last day of antiretroviral therapy the following procedures will be performed:

- Medication adherence history
- Perform a complete physical exam
- ECG, 12 lead
- Obtain vital signs and review concomitant medications
- Record adverse events
- Obtain laboratory samples for the following:
    - Chemistry and hematology
    - Urinalysis (dipstick)
    - Urine pregnancy test (β-HCG) on females of childbearing potential
    - CD4+ cell count
    - Plasma HIV-1 RNA
    - Lymphocyte immunophenotyping
    - PBMC (stored specimen)
    - Serum for neutralizing antibodies (stored specimen)
    - Plasma for genotypic resistance (stored specimen)
    - IL-6, d-Dimer, hsCRP (stored specimens)
- Discontinuation of ART

Study Day TI-15 (TI Week 3)

- Perform a symptom-driven physical exam (including weight measurement)
- Obtain vital signs and review concomitant medications
- Record adverse events
- Obtain laboratory samples for the following:
    - Chemistry and hematology
    - Urinalysis (dipstick)
    - Urine pregnancy test (β-HCG) on females of childbearing potential
    - CD4+ cell count
    - Plasma HIV-1 RNA
    - Lymphocyte immunophenotyping
    - Plasma for genotypic resistance (stored specimen)
    - IL-6, d-Dimer, hsCRP (stored specimens)

Study Day TI-29 (TI Week 5)

- Perform a symptom-driven physical exam (including weight measurement)
- Obtain vital signs and review concomitant medications

- Record adverse events
- Obtain laboratory samples for the following:
    - Chemistry and hematology
    - Urinalysis (dipstick)
    - Urine pregnancy test (β-HCG) on females of childbearing potential
    - CD4+ cell count
    - Plasma HIV-1 RNA
    - Lymphocyte immunophenotyping
    - PBMC (stored specimen); collected only if virus is quantifiable at week 3
    - Serum for neutralizing antibodies if virus is quantifiable at week 3 (stored specimen)
    - Plasma for genotypic resistance (stored specimen)
    - IL-6, d-Dimer, hsCRP (stored specimens)

Study Day TI-43 (TI Week 7)

- Perform a symptom-driven physical exam (including weight measurement)
- Obtain vital signs and review concomitant medications
- Record adverse events
- Obtain laboratory samples for the following:
    - Chemistry and hematology
    - Urinalysis (dipstick)
    - Urine pregnancy test (β-HCG) on females of childbearing potential
    - CD4+ cell count
    - Plasma HIV-1 RNA
    - Lymphocyte immunophenotyping
    - PBMC (stored specimen); collected only if virus is quantifiable at week 5
    - Serum for neutralizing antibodies if virus is quantifiable at week 5 (stored specimen)
    - Plasma for genotypic resistance (stored specimen)
    - IL-6, d-Dimer, hsCRP (stored specimens)

Study Day TI-57 (TI Week 9)

- Perform a symptom-driven physical exam (including weight measurement)
- Obtain vital signs and review concomitant medications
- Record adverse events
- Obtain laboratory samples for the following:
    - Chemistry and hematology
    - Urinalysis (dipstick)
    - Urine pregnancy test (β-HCG) on females of childbearing potential

  - CD4+ cell count
  - Plasma HIV-1 RNA
  - Lymphocyte immunophenotyping
  - PBMC (stored specimen); collected only if virus is quantifiable at week 7
  - Serum for neutralizing antibodies if virus is quantifiable at week 7 (stored specimen)
  - Plasma for genotypic resistance (stored specimen)
  - IL-6, d-Dimer, hsCRP (stored specimens)

Study Day TI-71 (TI Week 11)

- Perform a symptom-driven physical exam (including weight measurement)
- Obtain vital signs and review concomitant medications
- Record adverse events
- Obtain laboratory samples for the following:
  - Chemistry and hematology
  - Urinalysis (dipstick)
  - Urine pregnancy test (β-HCG) on females of childbearing potential
  - CD4+ cell count
  - Lymphocyte immunophenotyping
  - PBMC (stored specimen); collected only if virus is quantifiable at week 9
  - Serum for neutralizing antibodies if virus is quantifiable at week 9 (stored specimen)
  - Plasma HIV-1 RNA
  - Plasma for genotypic resistance (stored specimen)
  - IL-6, d-Dimer, hsCRP (stored specimens)

Analytic Treatment Interruption Phase Early Termination (TI-ET)

- Perform a complete physical exam (including weight measurement)
- 12-Lead ECG
- Obtain vital signs and review concomitant medications
- Record adverse events
- Obtain laboratory samples for the following:
  - Chemistry and hematology
  - Urinalysis (dipstick)
  - Urine pregnancy test (β-HCG) on females of childbearing potential
  - CD4+ cell count
  - Plasma HIV-1 RNA
  - Lymphocyte immunophenotyping
  - PBMC (stored specimen)

        - Serum for neutralizing antibodies (stored specimen)
        - Plasma for genotypic resistance (stored specimen)
        - IL-6, d-Dimer, hsCRP (stored specimens)
- Reinstitution of ART

Extended Analytic Treatment Interruption Phase

All patients are counseled to restart treatment after the 12 week scheduled period of interruption. If a patient has a sustained low level of viremia, and declines to restart treatment, the sponsor may elect to allow the patient to continue in the study in an extended treatment interruption period, if the patient's primary care physician and the Principal Investigator agree. Once the approval is granted, the patient will be followed every 4 weeks after the T-I 6 visit until the viral load and/or CD4+ thresholds are reached, or sooner if deemed appropriate for the patients' safety. Followup visits after the T-I 6 visit will consist of the same assessments and labs as the T-I 6 visit and will be labeled T-I7, T-I 8, T-I 9 etc. until the patient starts back on ART. When and if the decision is made to reinstitute ART, the patient will come in for the Treatment Reinstitution 1 visit and then follow the schedule of events for the remainder of their visits.

7.4.4 Treatment Reinstitution: Schedule of Study Visits

Reinstitution of treatment occurs at Treatment Reinstitution Visit 1 (Visit R-1, Study Week R-1). For patients who complete the full 12 weeks of analytic treatment interruption, this visit occurs 2 weeks after TI-Week 11. For patients who complete extended analytic treatment interruption (>12 weeks), this visit occurs 2 weeks after the last and final TI visit. For patients who require treatment reinstitution earlier, the Analytic Treatment Interruption Early Termination visit may substitute for Visit R-1. These patients will resume follow-up with Study Week R-5.

Study Week R-1 (Visit R-1)

- Perform a complete physical exam (including weight measurement)
- 12-Lead ECG
- Obtain vital signs and review concomitant medications
- Record adverse events
- Obtain laboratory samples for the following:
    - Chemistry and hematology
    - Urinalysis (dipstick)
    - Urine pregnancy test (HCG) on females of childbearing potential
    - CD4+ cell count
    - Plasma HIV-1 RNA
    - Lymphocyte immunophenotyping
    - PBMC (stored specimen)

- Serum for neutralizing antibodies (stored specimen)
    - Plasma for genotypic resistance (stored specimen)
    - IL-6, d-Dimer, hsCRP (stored specimens)
- Reinstitution of ART

Study Weeks R-5 (Visit R-2) and R-9 (Visit R-3)

- Medication adherence history
- Perform a symptom-driven physical exam (including weight measurement)
- Obtain vital signs and review concomitant medications
- Record adverse events
- Obtain laboratory samples for the following:
    - Chemistry and hematology
    - Urinalysis (dipstick)
    - Urine pregnancy test (β-HCG) on females of childbearing potential
    - CD4+ cell count
    - Plasma HIV-1 RNA
    - Lymphocyte immunophenotyping
    - Plasma for genotypic resistance (stored specimen)
    - IL-6, d-Dimer, hsCRP (stored specimens)

Study Week R-13 (Visit R-4)

- Medication adherence history
- Perform a symptom-driven physical exam (including weight measurement)
- Obtain vital signs and review concomitant medications
- Record adverse events
- Obtain laboratory samples for the following:
    - Chemistry and hematology
    - Urinalysis (dipstick)
    - Urine pregnancy test (β-HCG) on females of childbearing potential
    - CD4+ cell count
    - Plasma HIV-1 RNA
    - Lymphocyte immunophenotyping
    - PBMC (stored specimen)
    - Serum for neutralizing antibodies (stored specimen)
    - Plasma for genotypic resistance (stored specimen)
    - IL-6, d-Dimer, hsCRP (stored specimens)

Study Weeks R-17 (Visit R-5) and R-21 (Visit R-6)

- Medication adherence history

- Perform a symptom-driven physical exam (including weight measurement)
- Obtain vital signs and review concomitant medications
- Record adverse events
- Obtain laboratory samples for the following:
    - Chemistry and hematology
    - Urinalysis (dipstick)
    - Urine pregnancy test (β-HCG) on females of childbearing potential
    - CD4+ cell count
    - Plasma HIV-1 RNA
    - Lymphocyte immunophenotyping
    - Plasma for genotypic resistance (stored specimen)
    - IL-6, d-Dimer, hsCRP (stored specimens)

Early Termination Visit (ET)/Study Termination Visit (Week 25)

- Medication adherence history
- Perform a complete physical exam (including weight measurement)
- 12-Lead ECG
- Obtain vital signs and review concomitant medications
- Record adverse events
- Obtain laboratory samples for the following:
    - Chemistry and hematology
    - Urinalysis (dipstick)
    - Urine pregnancy test (β-HCG) on females of childbearing potential
    - CD4+ cell count
    - Plasma HIV-1 RNA
    - Lymphocyte immunophenotyping
    - PBMC (stored specimen)
    - Serum for neutralizing antibodies (stored specimen)
    - Plasma for genotypic resistance (stored specimen)
    - IL-6, d-Dimer, hsCRP (stored specimens)

7.4.5 Procedure for Antiretroviral Therapy Discontinuation for Patients Not on Efavirenz

Patients not on efavirenz will discontinue ART at week 33. This visit will serve as Analytic Treatment Interruption Visit 1. Following drug discontinuation, patients will continue the schedule for the Analytic Treatment Interruption Phase.

7.4.6 Procedure for Switching to Boosted Protease Inhibitor (BPI) or Raltegravir for Patients on Efavirenz

At week 31, patients who are on an efavirenz-containing regimen will switch efavirenz to a BPI regimen or raltegravir and continue the remainder of their ART regimen. Patients taking Atripla™ will change to Truvada™ and a boosted protease inhibitor or raltegravir. The preferred boosted protease inhibitor regimen will be ritonavir (Norvir™) 100 mg daily and atazanavir (Reyataz ™) 300 mg daily. Other BPI regimens will be considered on an individual basis in consultation with the Medical Monitor. Raltegravir (Isentress™) 400 mg bid is a first-in-class oral integrase inhibitor that can be substituted for the BPI regimen. The BPI or raltegravir regimen will be provided by GeoVax. Other drug components will be obtained in the way the patient usually obtains his/her medications.

7.5 Cardiac Safety Monitoring

7.5.1 Framingham Global Risk Assessment

Patients at high risk for cardiac disease will be excluded from the trial due to the increased risk of cardiac events associated with treatment interruption. The Framingham Global Risk Assessment will be used as a screening tool in this study. Patients must have a "low risk" score on this tool to be eligible for this study (Appendix E).

7.5.2 Cardiac Symptoms Assessment

At each study visit, patients will be assessed for the presence of new cardiac symptoms and change in intensity or frequency of pre-existing symptoms. Specifically, patients will be asked about the presence or absence of fever, chest tightness or pain, palpitations, edema, and dyspnea.

7.5.3 ECG Testing

Standard 12-lead ECG evaluation will occur at screening, baseline, termination of ART or early termination of the vaccination phase, reinstitution of ART and study termination.

7.5.4 Evaluation of Elevated Creatine Phosphokinase (CPK)

CPK testing will occur at each visit as part of the chemistry profile (defined separately under Laboratory Evaluations), the schedule of which is shown in Appendix B of the protocol.

Elevated CPK occurs commonly in the setting of HIV-1 infection, with a frequency of approximately 15%, and is usually associated with skeletal muscle, not cardiac muscle (49). The etiology may be HIV-1 itself or mitochondrial toxicity from antiretroviral drugs, particularly nucleoside reverse transcriptase inhibitors (50). Exercise can cause CPK elevation, which is typically transient and benign (51). Intramuscular injections can also cause low-grade transient CPK elevations.

Patients who experience CPK elevation of grade 1 or above while on study will undergo the following evaluation:

- Reflex measurement of fractionated isoenzymes to assess proportions attributable to skeletal muscle (MM), cardiac muscle (MB), and brain (BB)
- Reflex serum troponin I and T
- History to elicit possible causes, including a history of recent exercise or trauma, muscle pain, cardiac symptoms (see Section 7.5.2), or use of concomitant medications
- Advise patient to discontinue all exercise and to hydrate
- Repeat CPK for confirmation and complete metabolic profile (CMP) for monitoring of renal function and ALT/AST.

Toxicity Grading

- CPK > 3.0 – 9.9 x ULN (Grades 1 and 2):
    - Safety evaluation as described above
    - ECG
    - Suspension of immunization until CPK < grade 1, if non-cardiac
- CPK ≥ 10.0 – 19.9 x ULN (Grade 3):
    - Safety evaluation as described above
    - ECG
    - Echocardiogram if clinically indicated
    - Suspension of immunization until < grade 1, if non-cardiac
- CPK > 20.0 x ULN (Grade 4):
    - Safety evaluation as described above
    - ECG
    - Echocardiogram if not clearly associated with exercise
    - Suspension of immunization until < grade 1, if non-cardiac

In the case of CPK of any level with significant MB component or positive troponin, the patient will discontinue further immunizations and be followed for safety for the planned duration of patient participation. Such patients will not enter the analytic treatment interruption phase, or if occurring during analytic treatment interruption, will restart therapy at the discretion of the investigator and may continue in the trial for safety follow-up.

In the case of grade 3 or 4 CPK without MB elevation that does not return below grade 1 level within 4 weeks, the patient will discontinue further immunizations and be followed for safety for the planned duration of patient participation. Such patients will not enter the analytic treatment interruption phase, or if occurring during analytic treatment interruption, will restart therapy at the discretion of the investigator and may continue in the trial for safety follow-up.

7.5.5 Evaluation of Suspected Myopericarditis

Patients who report symptoms thought to be of cardiac origin, or who exhibit edema or abnormalities in the cardiac exam, will undergo an ECG, physical examination, assessment of serum troponin I and T levels, and CPK isoenzymes, and if indicated, an echocardiogram. These findings will be communicated to the primary care provider and appropriate additional cardiac workup will follow in the primary care setting. Documentation of this evaluation will be obtained for the study records.

7.6 Evaluation of Suspected MVA Replication

MVA is a highly attenuated recombinant strain of vaccinia virus that is replication defective in human cells (52, 53). MVA is reported to be safe in normal and immunosuppressed animals and in humans (54), including HIV-1 infected humans. Clinical studies in HIV-infected individuals of the IMVAMUNE® MVA (Bavarian Nordic) product for protection from smallpox have progressed through Phase 2 (ClinicalTrials.gov: NCT00390078, NCT00189956). The IMVAMUNE product was derived from the same poxvirus source material as the GeoVax MVA, and it was also safely tested as a vaccine vector in HIV-infected volunteers (55). No laboratory or hospital worker-acquired infections resulting from exposure to MVA have been published or reported.

Despite this, MVA replication is a theoretical safety concern in immunosuppressed subjects. Symptoms of MVA replication will be closely monitored. Skin lesions at the vaccination site will be evaluated for causation. Bacterial cultures will be collected, when possible, from the lesions. Other symptoms could include lymphadenopathy and fever. As an added safety precaution, subjects will receive the MVA vaccines in a staggered manner. After the first two patients receive their first MVA doses, additional subjects must wait at least 2 weeks before receiving their first MVA vaccine. In the event of suspected MVA replication, vaccinia immune globulin (VIG), which contains antibodies to vaccinia virus, will be administered by IM injection. If MVA replication is diagnosed, the patient will discontinue vaccination and will not participate in analytic treatment interruption, but will continue to be followed for safety until resolved.

For Georgia residents, VIG is available from the Georgia Department of Human Resources, Georgia Division of Public Health, to treat subjects enrolled in this study if they experience symptoms consistent with MVA replication. The sponsor should be notified immediately. In case of emergency, physicians should call the daytime telephone number of the Georgia Division of Public Health at (404) 657-2700 or the 24 hour/day every day CDC Emergency Operations Center at 1-866-210-6469. For all other subjects who experience symptoms consistent with MVA replication, including those who reside in Alabama, physicians should call the CDC Emergency Operations Center at 1-866-210-6469.

7.7 Change in Vaccination Schedule

Vaccination will be delayed for up to 2 weeks between vaccinations 1 and 2, vaccinations 2 and 3, and vaccinations 3 and 4 in any patient under the following circumstances:

- Any treatment emergent adverse event is present that is deemed by the investigator to place the patient at increased risk by continuing the vaccination schedule
- An unresolved grade 3 or 4 adverse event is present
- HIV-1 RNA > 50 copies/mL at the most recent time point prior to the immunization visit
- CD4+ cell count < 350 cells/µL at the most recent time point prior to the immunization visit

Participants who miss a scheduled vaccination visit may be rescheduled within 2 weeks after the initially scheduled visit for a minimum of 6 weeks between vaccinations to allow for an acceptable immune response. Adverse events will be monitored as determined by the investigator. HIV-1 RNA and/or CD4+ cell counts will be monitored at weekly or biweekly intervals as determined by the investigator.

7.8 Visit Window and Missed Visits

Acceptable visit windows are as follows:

Vaccination Phase Visits 4-21: Plus or minus 3 days
Analytic Treatment Interruption Phase Visits 1-6 or extended: Plus or minus 3 days
Reinstitution of Therapy Phase: Visits R-1-6: Plus or minus 3 days

A visit that is not performed within the window period must be explained within the subjects source document and recorded on the "Comments Page" of the case report form for GV-TH-01 related to the visit that is missed. If the missed visit is one requiring safety assessments or safety labs, the site should attempt to bring the participant in as soon as possible. If the missed visit was for a scheduled vaccination, the rescheduled visit should occur no more than 2 weeks after the scheduled vaccination (see Section 7.7, Change in Vaccination Schedule).

7.9 Vaccination Discontinuation

A participant who misses any vaccinations and does not reschedule the visit within 2 weeks after the initially scheduled visit will not be eligible to enter the analytic treatment interruption phase. However, these participants will be followed until the end of the vaccination phase of the study. Vaccinations will be discontinued in any participant who experiences an ongoing intercurrent illness that would, in the opinion of the investigator, potentially confer increased risk from vaccination or in any participant who is unable to comply with the vaccination schedule. Such patients will be followed until the end of the vaccination phase of the study but will not enter the analytic treatment interruption phase. Patients who meet the definition of virologic or immunologic failure will not receive further vaccinations or enter the analytic

treatment interruption phase but will be followed until the end of the vaccination phase of the study. Participants who choose to discontinue vaccinations at any time will be asked to remain in the study until the end of the vaccination phase for safety and immunogenicity follow-up. Patients who become pregnant during the immunization phase will cease vaccinations but will remain in the study through the end of the vaccination phase. Within 24 hours of site staff awareness of the pregnancy, a Pregnancy Report Form should be completed and faxed to **(301) 628-1931 (**Product Safety Unit at Social & Scientific Systems, Inc.). The outcome of the pregnancy should be reported when information is available.

7.10 Concomitant Medications

Immunomodulatory agents, including systemic steroids for more than 5 days, and other vaccinations are not allowed during the vaccination phase of the trial. Such therapy may only be given during the analytic treatment interruption or treatment reinstitution phases with approval of the medical monitor. The need for systemic chemotherapy will require study discontinuation. If, in the opinion of the Investigator, administration of systemic steroids is in the patient's best interest, the Medical Monitor (see page 4 for contact information) should be consulted to assess whether the patient can continue on trial.

7.11 Assessment of Reactogenicity

7.11.1 Assessment of Systemic and Local Symptoms

For all participants, reactogenicity assessments are performed after each vaccination. All reactogenicity symptoms are followed until resolution and graded according to the NIH Division of AIDS Table for Grading the Severity of Adult and Pediatric Adverse Events, Version 1.0, December 2004.

Reactogenicity assessment schedule

Day	Time	Responsibility
Day of vaccination (Day 1 of cycle)	Baseline: pre-vaccination	Site staff
Day of vaccination (Day 1 of cycle)	Early: within 60 minutes post vaccination	Site staff
Three days post vaccination (Day 4 of cycle)	Telephone call: 72-84 hours post vaccination	Site staff

7.12 Pregnancy

If a participant becomes pregnant during the course of the study, no further injections of study products will be administered. Within 24 hours of site staff awareness of the pregnancy, a Pregnancy Report Form should be completed and faxed to **(301) 628-1931 (**Product Safety Unit at Social & Scientific Systems, Inc.). Remaining visits and study procedures through the vaccination phase should be completed unless medically contraindicated. If the participant

terminates the study prior to the pregnancy outcome, the site will maintain contact with the participant in order to ascertain the pregnancy outcome. Pregnancy termination and outcome information should also be provided to the Product Safety Unit at Social & Scientific Systems within 24 hours of site staff awareness.

7.13 Definitions of Virologic Failure

Vaccination Phase: All patients will enter the study with HIV-1 RNA < 50 copies/mL. Virologic failure will be defined as HIV-1 RNA > 200 copies/mL and confirmed by a second measurement at least two weeks apart. No further vaccinations will be given once this definition has been reached.

Treatment Reinstitution Phase: Virologic failure will be defined as HIV-1 RNA >200 copies/mL at or beyond 12 weeks following re-institution of antiretroviral therapy, and confirmed upon two measures at least two weeks apart. Likewise, HIV-1 RNA rebound to >200 copies/mL following initial suppression will be considered virologic failure if confirmed upon two measures at least 2 weeks apart. These patients will be encouraged to change their regimen in consultation with their primary care provider if adherence is not in question. Genotypic resistance data will be provided to assist in this decision.

7.14 Definitions of Immunologic Failure

Vaccination Phase: Patients whose CD4+ cell count falls below 350 cells/µL on two consecutive measurements at least 2 weeks apart will be considered to have immunologic failure and no further vaccinations will be given.

7.15 Criteria for Reinstitution of Treatment During Analytic Treatment Interruption Phase

Treatment will be reinstituted when the CD4+ count falls below 350 cells/µL for two consecutive measurements at least 2 weeks apart or when re-emergent virus (HIV-1 RNA) reaches levels of >300,000 copies per mL of plasma for two consecutive measurements at least 2 weeks apart.

8. LABORATORY

8.1 Safety Laboratory Assessments

Serum electrolytes, BUN, CPK, serum creatinine, creatinine clearance, glucose, AST, ALT, total bilirubin, microscopic urinalysis, lipid profile, complete blood count, and platelet count will be performed by local clinical trial site laboratories.

8.2 Virology Assays

HIV-1 RNA quantification for all subjects will be performed by local clinical trial site laboratories using the Roche Amplicor® method for quantitative real-time polymerase chain reaction (RT-PCR) with a lower limit of quantification of 50 copies/mL.

Genotypic resistance testing will be performed by the respective local laboratories for each of the trial sites.

Plasma for HIV-1 RNA and genotypic resistance testing will be prepared according to standard methodology.

8.3 Immunology Assays

8.3.1 Cellular Immune Responses

Determination of CD4+ T cell absolute number and percentage

CD4+ lymphocyte counts will be determined for safety monitoring and will be performed by local clinical trial site laboratories using 4-color multiparametric flow cytometry.

Lymphocyte immunophenotyping

Lymphocyte types will be defined using antibodies specific for specific cell-surface markers (CD3/4, CD3/8, CD19, CD8/CD38/DR, CD4/CD38/DR) and flow cytometry; assays will be performed at GeoVax, Inc. for ARCA samples, the University of Alabama, and ARA according to research protocols. All analyses of flow data will be performed at GeoVax.

Intracellular cytokine staining (ICS)

ICS will be used to test for and characterize responding CD4+ and CD8+ T cells. PBMC will be stimulated with synthetic HIV-1 peptides (15mers overlapping by 11) that span the Gag, Pr, RT, and Env proteins encoded by the vaccine construct and then flow cytometry will be used to enumerate HIV-specific CD4+ and CD8+ T-cell responses using IFN-γ and IL-2 ICS. These data will be obtained pre-immunization, at 2 weeks following the 2nd DNA immunization, at 1 week following the 1st and 2nd MVA immunizations, at the termination of ART, at 2 weeks after the detection of re-emergent virus during analytic treatment interruption and at 3 and 6 months after ART re-initiation. Data will be reported as percentages of CD4+ or CD8+ T cells recognizing a specific peptide pool. TNFα production by PBMC and CD8+ suppressor activity may also be assayed. These studies will be completed at the GeoVax, Inc. Laboratory according to research protocols.

Epitope Mapping

Mapping of specific epitopes, in peptide form, recognized by vaccine-induced Gag-specific CD8+ T cell responses and post re-emergent viral Gag-specific CD8+ T cell responses will be conducted to evaluate the match of CD8+ epitopes in the peak vaccine-elicited response to the

CD8+ epitopes stimulated by the re-emergent virus at 2 weeks after the detection of re-emergent virus during analytic treatment interruption. Mapping will be conducted using the same Gag peptides used in ICS assays.

8.3.2 Humoral

Antibodies specific for Env will be measured at the GeoVax, Inc. Laboratory by ELISA. These data will be obtained pre-immunization; at 2 weeks following the 2nd DNA immunization; at 2 weeks following the 1st and 2nd MVA immunizations; at the termination of ART; at 4, 8, and 12 weeks after ART termination; and at 3 months and 6 months after ART re-institution. Data will be reported as estimated ng/mL of binding antibody/mL of serum. Avidity will be tested using duplicate ELISA, one receiving a 1.5 M NaSCN wash and one a PBS wash. For these assays the avidity index is the % of the antibody retained at an OD of 0.5 following the NaSCN wash (12, 56).

Neutralizing antibody for incident clade B isolates

Assays will be performed at the discretion of GeoVax in collaboration with Dr. David Montefiori at Duke University.

Antibody-Dependent Cell Mediated Virus Inhibition (ADCVI) assays

Assays will be performed at the discretion of GeoVax in collaboration with Dr. Donald Forthal of the University of California, Irvine (57).

8.4 Human Leukocyte Antigen (HLA) Tissue Typing

HLA Class I tissue typing will be performed to evaluate vaccine-elicited immune responses. The HLA tissue typing will be performed by standard methods using frozen PBMCs from stored samples.

8.5 Inflammatory Marker Assays

IL-6, d-Dimer and hsCRP assays will be performed following the completion of the study for all subjects. Samples will be collected, frozen, stored at -70 °C, batched, and shipped to GeoVax for storage until tested.

8.6 Pharmacology Assays

Assays for efavirenz serum concentrations will be performed by reference laboratories for all sites. Serum samples will be stored at -70°C for assays.

8.7 Future Use of Stored Specimens

The protocol informed consent form is written so that the participant either explicitly allows or does not allow sample storage for future research when he or she signs the form. Participants who initially agree to future use of their samples may rescind their approval once they enter the study; such participants will still remain in this study. If a participant decides against allowing

future research using his or her samples, the subject must notify ARCA, UAB or ARA in writing. After study data analysis is complete, all specimens with that participant's identification number will be destroyed.

8.8 Total Blood Volume

Immunization phase: Participants on ART that does not include efavirenz: approximately 1030 mL; participants on ART that includes efavirenz: approximately 1100 mL
Analytic treatment interruption phase: approximately 485 mL
Extended analytic treatment interruption phase: >485 mL
Treatment reinstitution phase: approximately 485 mL

8.9 Biohazard Containment

As the transmission of HIV-1 and other blood-borne pathogens can occur through contact with contaminated needles, blood, and blood products, appropriate precautions will be employed by all personnel in the drawing of blood and shipping and handling of all specimens for this study (as currently recommended by the CDC and the NIH or other locally appropriate agencies).

All dangerous goods materials, including Biological Substances, Category A or Category B, must be transported according to instructions detailed in the International Air Transport Association (IATA) Dangerous Goods Regulations.

9. STATISTICAL ANALYSIS

Detailed methodology for summary and statistical analyses of the data collected in this study will be documented in a Statistical Analysis Plan, which will be maintained by the sponsor. This document may modify the plans outlined in the protocol; however, any major modifications of the primary endpoint definition and/or its analysis will also be reflected in a protocol amendment.

9.1 Analysis of Primary, Secondary, and Exploratory Endpoints

9.1.1 Primary Endpoint: Safety

Descriptive analysis only will be performed due to the small sample size in this exploratory study.

All Phases:

- Frequency and severity of clinical adverse events
- Frequency and severity of laboratory abnormalities
- Frequency and severity of local and systemic reactogenicity signs and symptoms following vaccinations

Vaccination Phase:

- Re-emergence of HIV-1 RNA to levels >200 copies per mL of plasma, confirmed by two consecutive values at least 2 weeks apart
- Drop in CD4+ cell count to below 350 cells/µL, confirmed by two consecutive values at least 2 weeks apart
- Genotypic resistance patterns of re-emergent virus

Analytic Treatment Interruption Phase:

- Number and percentage of participants meeting HIV-1 RNA criteria for re-institution of treatment prior to 12 weeks
- Number and percentage of participants meeting CD4+ count criteria for re-institution of treatment prior to 12 weeks
- Number and percentage of participants with emergence of genotypic resistance in re-emergent virus

Treatment Reinstitution Phase:

- Number and percentage of participants meeting criteria for virologic failure
- Number and percentage of participants with emergence of genotypic resistance in patients meeting criteria for virologic failure

9.1.2 Secondary Endpoints

Descriptive analysis only will be performed due to the small sample size in this exploratory study.

Vaccine-elicited immune responses:

- Magnitude of interferon gamma and/or IL-2 producing CD4+ and CD8+ T cells at 1 week post the 2nd MVA vaccination
- Number and identity of Gag-specific CD8+ T cell responses at 1 week post the 2nd MVA vaccination
- Titer and avidity index of binding antibody for Env at 2 weeks post the 2nd MVA vaccination

Anti-HIV-1 immune responses during the analytic treatment interruption phase:

- Magnitude of interferon gamma and/or IL-2 producing CD4+ T cells and CD8+T cells at 2 weeks post detection of re-emergent virus
- Number and identity of Gag-specific CD8+ T cell responses at 2 weeks post the detection of re-emergent virus
- Titers of binding antibody for Env at 2 weeks post the detection of re-emergent virus

Viral control during the analytic treatment interruption phase analyzed separately for those who started ART at <8 weeks as opposed to >8 weeks post their acute infection :

- Logarithmic change in HIV-1 RNA level from pre-institution of ART to termination of analytic treatment interruption
- Time to HIV-1 RNA rebound to >200 copies/mL

9.1.3 Exploratory Endpoints

Correlations between vaccine responses and the control of re-emergent virus conducted separately for those who started ART at <8 weeks as opposed to >8 weeks post their acute infection:

- Correlation between the magnitudes of interferon gamma and/or IL-2 producing CD4+ and CD8+ T cell responses at 1 week after the second MVA vaccination and the logarithmic changes in the level of historical HIV-1 RNA pre-institution of ART compared to that at the termination of analytic treatment interruption
- Correlation between the number and identity of Gag-specific CD8+ T cells at 1 week after the 2nd MVA inoculation that were matched to those stimulated at 2 weeks after the re-emergence of virus during analytic treatment interruption and the logarithmic changes in the level of historical HIV-1 RNA pre-institution of ART compared to that at the termination of analytic treatment interruption
- Correlation between the titer or avidity of the Env-specific antibody response at 2 weeks after the 2nd MVA vaccination and the logarithmic changes in the level of historical HIV-1 RNA pre-institution of ART compared to that at the termination of analytic treatment interruption.
- Correlation between the magnitude of vaccine-elicited interferon gamma and/or IL-2 producing CD4+ and CD8+ T cells at 1 week after the 2nd MVA inoculation and time to HIV-1 RNA rebound to >200 copies/mL during analytic treatment interruption
- Correlation between the number and identity of Gag-specific CD8+ T cells at 1 week after the 2nd MVA inoculation that were matched to those stimulated at 2 weeks after the re-emergence of virus during analytic treatment interruption and time to HIV-1 RNA rebound to >200 copies/mL during analytic treatment interruption
- Correlation between the titer or avidity of anti-Env antibody at 2 weeks after the 2nd MVA vaccination and time to HIV-1 RNA rebound to >200 copies/mL during analytic treatment interruption
- Correlation between the avidity of anti-Env antibody at 2 weeks after the 2nd MVA vaccination and time to HIV-1 RNA rebound to >200 copies/mL during analytic treatment interruption

Correlations between vaccine-induced responses and the levels of inflammation during analytic treatment interruption conducted separately for those who started ART at <8 weeks as opposed to >8 weeks post their acute infection:

- Correlation between the magnitude of interferon gamma and/or IL-2 producing vaccine-elicited CD4+ and CD8+ responses at 1 week after the 2nd MVA vaccination and the areas under the curve for IL-6, d-Dimer and hsCRP during analytic treatment interruption
- Correlation between the number and identity of Gag-specific CD8+ T cells at 1 week after the 2nd MVA vaccination that were matched to those stimulated at 2 weeks after the re-emergence of virus during analytic treatment interruption and the areas under the curve for IL-6, d-Dimer and hsCRP during analytic treatment interruption
- Correlation between the titer and avidity index of Env-specific antibody at 2 weeks after the 2nd MVA vaccination and the areas under the curve for IL-6, d-Dimer and hsCRP during analytic treatment interruption

9.2 Analysis Data Sets

9.2.1 Intent-to-Treat Analysis

All participants who receive the baseline vaccination will be included in the intent-to-treat (ITT) analysis.

9.2.2 Per-Protocol Analysis

Participants who receive all four vaccinations will be included in the per-protocol (PP) analysis.

9.2.3 Statistical Methods

For the primary and secondary endpoints, descriptive analysis only will be performed due to the small sample size in this exploratory study. For the exploratory endpoints, the Spearman rank correlation will be used for non-parametric data and the Pearson correlation for parametric data. No adjustments will be made for multiple analyses.

9.3 Sample Size and Power Calculations

This is an exploratory study to derive preliminary information about safety and immunogenicity in a small cohort prior to expanding to a randomized, proof of concept trial. Approximately 10 evaluable participants, defined as those who receive all immunizations and undergo analytic treatment interruption as scheduled or who meet a criterion for failure before doing so, will be enrolled. Participants that discontinue participation in the trial prior to completion of vaccinations and ART interruption will be replaced. While information will be gathered to explore the antiviral activity of the vaccine regimen, the sample size is not powered to definitively estimate activity or efficacy.

10. SAFETY MONITORING AND SAFETY REVIEW

10.1 Adverse Events and Serious Adverse Events

10.1.1 Definitions

An adverse event (AE) is any unfavorable and unintended sign, symptom, or illness temporally associated with the use of an investigational study product/procedure(s), whether or not related to the investigational study product/procedure(s). An AE also may be a worsening of a pre-existing symptom or illness.

A serious adverse event (SAE) is any medical occurrence that:

- Results in death
- Is life threatening at the time of the event as it occurred (the patient was at an immediate risk of death at the time of the SAE)
- Requires inpatient hospitalization or prolongation of an existing hospitalization
- Results in persistent or significant disability/incapacity defined as a substantial disruption of a person's ability to conduct normal life functions
- Is a congenital anomaly or birth defect
- Is an important medical event that, when based upon appropriate medical judgment, may jeopardize the patient and may require medical or surgical intervention to prevent one of the outcomes listed above in the definition for a serious adverse event. (Examples of such events include allergic bronchospasm requiring intensive treatment at an emergency room or at home, blood dyscrasias, convulsions that do not result in inpatient hospitalization, or the development of drug dependency or drug abuse)

10.2 Classification and Reporting of Adverse Events

All adverse events are graded for intensity according to the *Division of AIDS Table for Grading the Severity of Adult and Pediatric Adverse Events* (DAIDS AE Grading Table), Version 1.0, December 2004, available at http://rcc.tech-res-intl.com). The principal investigator determines the clinical significance of an adverse event. Worsening in intensity of a previous adverse event will be considered a new adverse event. For clinical events not covered by the DAIDS grading system, the following classification will be used:

- GRADE 1: Mild symptoms causing no or minimal interference with usual social and functional activities
- GRADE 2: Moderate symptoms causing greater than minimal interference with usual social and functional activities
- GRADE 3: Severe symptoms causing inability to perform usual social and functional activities
- GRADE 4: Potentially life threatening symptoms causing inability to perform basic self-care functions OR medical or operative intervention indicated to prevent permanent impairment, persistent disability, or death except:
  - Unintentional weight loss of less than 10% in body weight from baseline is not required to be reported as an AE
  - PR interval $\leq$ 0.219 sec will not be reported as an AE and

- Asymptomatic increase in QTc interval < 0.06 sec above baseline, with a QTc ≤ 0.45 sec, will not be reported as an AE

The definition of Grade 1 mild prolonged PR interval (Adult > 16 years) that will be used is 0.22 – 0.25 sec.

The criteria for prolonged QTc interval that will be used are the following:

- Grade 1, mild: asymptomatic, QTc interval 0.45–0.47 sec
- Grade 2, moderate: asymptomatic, QTc interval 0.48–0.49 sec
- Grade 3 severe: asymptomatic, QTc interval ≥ 0.50 sec OR increase in interval ≥ 0.06 sec above baseline
- Grade 4, potentially life-threatening: life-threatening consequences (e.g., Torsade de Pointes or other associated serious ventricular dysrhythmia)

Adverse events will be classified according to their relationship to a study vaccine. The investigator will assess all adverse events and determine relationship to study vaccine using the following categories:

- **Unrelated**: no temporal relationship to a vaccine administration or the adverse event is clearly and incontrovertibly due only to progress of the underlying disease, or to extraneous causes.
- **Unlikely Related**: indicates that there is little or no chance that a study vaccine caused the reported adverse event; the event is most likely due to another competing cause, including concomitant illnesses, progression or expression of the disease state, or a reaction to a concomitant medication appearing to explain the reported adverse event.
- **Possibly Related**: indicates that the association of the adverse event with the study vaccine is unknown; however, the adverse event is not reasonably attributed to any other condition.
- **Probably Related**: indicates that a reasonable temporal association exists between the adverse event and study vaccine, and based upon the Investigator's clinical experience, there is no other obvious competing cause. The event resolves following time of receipt of the study vaccine
- **Definitely Related:** There is a likely causal relationship between the study vaccine and the AE, when the event resolves after vaccination and recurs with repeated administration of the vaccine.

All adverse events are reported on the appropriate case report form using standard medical terminology. Adverse events will be coded using MedDRA. When possible, a diagnosis for the underlying illness should be reported rather than individual symptoms. Adverse events that are possibly, probably, or definitely related to study drug should be followed until resolution.

Serious adverse events must be reported within 24 hours of site staff awareness of the event. The study contact for reporting serious adverse events is:

Product Safety Unit
Social & Scientific Systems, Inc.
SAE Reporting Fax Number: (301) 628-1931

AND

Harriet Robinson, PhD
GeoVax, Inc.
(678) 384-7220 office phone
(678) 384-7281 fax

Investigators may also call (919) 599-3785 to report a SAE. However, a completed SAE Report Form must be provided to the Product Safety Unit within no more than 24 hours of knowledge of the SAE. It is preferred that investigators fax the SAE Report Form.

Serious adverse events are also recorded on the appropriate case report form.

The Investigator will report all serious adverse events to the appropriate Institutional Review Board (IRB) in a timely manner.

10.3 Emergency Contact

In case of an emergency, the Investigator should contact the following:

Jean T. Barbey, MD
Medical Director/Monitor
Social & Scientific Systems, Inc.
8757 Georgia Avenue, Room #9030
Silver Spring, MD 20910
Office Phone : 301-628-3316
Mobile Phone: 202-251-6523
E-mail: jtbarbey@s-3.com

10.4 Pregnancy

Pregnancy, in and of itself, is not regarded as an adverse event, unless there is suspicion that study medication may have interfered with the effectiveness of a contraceptive medication or method. Within 24 hours of site staff awareness of a pregnancy, a Pregnancy Report Form should be completed and faxed to **(301) 628-1931 (**Product Safety Unit at Social & Scientific Systems, Inc.). No further vaccinations should be administered. A serum pregnancy test will be performed for confirmation. The patient may continue to be followed on study until the end

of the vaccination phase for safety monitoring. If the pregnancy occurs during analytic treatment interruption, the primary care provider and the patient will decide on the timing of reinstitution of therapy. The patient may continue to be followed on study for safety monitoring until the end of the treatment reinstitution phase. If the pregnancy occurs during treatment reinstitution, the primary care provider and the patient will decide whether to continue the current treatment regimen or change to a different regimen appropriate for pregnancy. If the patient prematurely discontinues, an early termination study visit will occur. Any pregnancy diagnosed during the study, or that occurs within 28 days after stopping study medication, must be reported immediately to the Product Safety Unit at Social & Scientific Systems, Inc. Pregnancy termination and outcome information should also be provided to the Product Safety Unit at Social & Scientific Systems within 24 hours of site staff awareness. An elective induced abortion is not an adverse event unless a complication ensues, but should be reported to the Product Safety Unit immediately.

All pregnancies must be reported to the Antiretroviral Pregnancy Registry. Information and forms can be requested at:

Phone: (800) 258-4263
Fax: (800) 800-1052
Email: registries@Kendle.com

10.5 Sponsor Reporting of Serious Adverse Events

The Sponsor is responsible for reporting 7- and 15-day SAE to the FDA in a manner consistent with federal guidelines and for following up all adverse events as appropriate. The Sponsor will provide the Investigator with a safety report from all Suspected Unexpected Serious Adverse Reactions (SUSAR) reported from any trial worldwide containing these vaccines. The Investigator will submit all SUSARs to the local IRB in a timely manner.

10.6 Monitoring of Adverse Events and Serious Adverse Events

The site and Sponsor (including its designees) will hold biweekly conference calls (when warranted) following enrollment of the first subject and throughout the time that any subject is on study. The Investigator or designee will report to the Sponsor on any adverse events of Grade 3 or above and any SAE that have occurred, including follow-up on previous SAE. AE and SAE will be monitored at the site during periodic monitoring visits.

11. DATA MANAGEMENT

11.1 Data Collection

All study data will be collected on the appropriate case report forms (CRF). Data entered on CRFs must be verifiable in source documents, and source documents must be available for monitoring. The investigator must sign final CRF to acknowledge accuracy and validity. The

Sponsor or its designee will be responsible for maintaining all study data in a study database and for providing reports for statistical analysis and sponsor review.

11.2 Data Monitoring

All original source documents and CRF must be available for periodic monitoring by the sponsor or designee. Queries will be generated by the sponsor or its designee and must be resolved by the site in a timely manner. The site must make adequate time and space available for study monitoring visits.

11.3 Confidentiality

Patient names will not be supplied to the Sponsor. Only the patient number and patient initials will be recorded in the CRF. If the patient's name appears on any other document (e.g., laboratory report), it must be obliterated on the copy of the document to be supplied to the Sponsor. The patients will be informed that representatives of the Sponsor, IRB, or regulatory authorities may inspect their medical records to verify information collected, and that all personal information made available for inspection will be handled in strictest confidence and in accordance with local data protection laws.

12. INVESTIGATOR AND SPONSOR RESPONSIBILITIES

12.1 Investigator Responsibilities

12.1.1 Protocol Adherence

The investigator will conduct the study in accord with the most current written and approved protocol document. Deviations from the protocol will be documented and submitted to the site IRB according to local requirements. Persistent deviation from the protocol or willful neglect thereof will be grounds for termination of the site's participation in the study.

12.1.2 Delegation of Authority

Staff implementing the protocol at the site will be trained to do so and delegation of the Investigator's authority will be documented on the Delegation Log and signed by the investigator. Copies of staff resumes will be kept on file at the site and available for monitoring.

12.1.3 Regulatory Approval

The Investigator will assure that the study will be supervised by an Institutional Review Board in good standing and holding a current Federal-wide Assurance Number. The Investigator will prospectively submit the protocol, informed consent form, investigator's brochure and other documents provided by the Sponsor as well new protocol amendments, informed consent revisions, investigator's brochures, advertisements, patient educational material, serious and/or unexpected adverse event reports, and other materials as directed by the Sponsor, to the IRB

for review. All IRB approvals will be kept on file at the site and a copy forwarded to the Sponsor. The Investigator will be responsible for periodic updates to the IRB regarding the conduct of the study, according to local requirements but annually at a minimum.

The only circumstance in which an amendment may be initiated prior to IRB approval is where the change is necessary to eliminate apparent immediate hazards to the subjects. In that event, the investigator must notify the IRB and the Sponsor in writing immediately after the implementation.

12.1.4 Informed Consent

The Investigator is responsible for obtaining written informed consent from each participant being screened for the study using a sponsor and IRB-approved informed consent form that is in compliance with FDA and local regulatory and legal requirements. The informed consent form will be explained to the participant and adequate time given to address questions. The informed consent must be obtained prior to any study procedures. The informed consent form will be signed and dated by the participant and by the person obtaining consent. Any changes to the informed consent document must be approved in writing by the sponsor and the IRB before use.

12.1.4.1 Pre-Screening Informed Consent

The Investigator may obtain a Pre-Screening Informed Consent from patients meeting preliminary eligibility criteria and who are interested in this study pending eligibility for inoculation. The purpose of this pre-screening informed consent is to enable sites to gather patient data ensuring eligibility for the trial for the period up to the baseline visit. This will also help to engage the patient and foster a relationship between the patient and the research site staff in preparation for trial enrollment.

12.1.5 Drug Accountability

The Investigator is responsible for ensuring accurate accountability for all investigational product referenced in this protocol, whether used or unused. Records will include receipt of vaccine shipment, dispensing records, and unused or destroyed study products. Vaccine supply and accountability will be periodically reviewed by the study monitor.

12.1.6 Record Retention

To enable evaluations and/or audits from regulatory authorities or the Sponsor, the investigator agrees to keep records, including the identity of all participating subjects (sufficient information to link records, e.g., CRF and hospital records), all original signed informed consent forms, copies of all CRF, serious adverse event forms, source documents, and detailed records of treatment disposition, and adequate documentation of relevant correspondence (e.g., letters, meeting minutes, telephone calls reports). The records should be retained by the

investigator according to ICH, local regulations, or as specified in the Clinical Study Agreement, whichever is longer.

12.2 Sponsor Responsibilities

12.2.1 Protocol Modifications

The Sponsor will be responsible for making all protocol modifications, except those deemed by the Investigator to be required to prevent immediate harm to a patient. The Sponsor will supply the site with protocol amendments for IRB submission.

12.2.2 Study Report and Publications

The Sponsor will be responsible for generating the study report required for submission to regulatory agencies, in accordance with FDA-approved guidances and guidelines and the ICH Guideline for Structure and Content of Clinical Study Reports. The Sponsor will be responsible for initial public disclosure of study results, in collaboration with the Investigator. The Investigator will submit any proposed publications or presentations of study results to the Sponsor in advance of submission. Further guidance on publications will be as outlined in the Clinical Study Agreement.

12.2.3 Conduct of Study in Accord with FDA Requirements

The Sponsor will be responsible for conducting the study in accord with all requirements stipulated by the US Food and Drug Administration (FDA). If, for any reason, the FDA requires the study to temporarily suspend or permanently terminate, the Sponsor will inform the Investigator immediately.

12.2.4 Retention and Destruction of Stored Samples

The Sponsor will be responsible for retention of stored samples and their appropriate destruction.

12.3 Shared Responsibilities of the Investigator and the Sponsor

12.3.1 Ethical Study Conduct

The study will be conducted in accordance with the Declaration of Helsinki (1996) on Ethical Principles for Medical Research Involving Human Subjects, and US regulations on Good Clinical Practice, FDA regulations as outlined in 21 CFR 312.30, 312.32, and Subpart D, and applicable local regulatory requirements and laws.

12.3.2 Confidentiality

Both the Investigator and the Sponsor will protect patient confidentiality to the highest level allowed by law. The Investigator will assure appropriate protection of records containing identifying information. The site will adhere to HIPAA regulations and local legal requirements. No names will be placed on documents transmitted to the sponsor. The sponsor

will not collect any documents containing patient names, addresses, or contact information unless those items are blacked out on the sponsor copy.

The Investigator will keep confidential the study protocol and all study documents including CRFs provided by the Sponsor, except as needed to properly conduct the study. Provision to site staff, IRB, and sub-investigators is allowed. Information contained in these documents may not be disclosed to any outside party without the prior written permission of the Sponsor.

The Sponsor will not disclose any site confidential information that may have been shared during the conduct of the trial, including site contracts, personnel information, banking information, or other proprietary documents.

12.3.3 Premature Study Discontinuation

Both the Sponsor and the Investigator have the right to terminate the study at any time. In the case of premature study discontinuation, both the Sponsor and the Investigator will take all necessary measures to protect the interests of the study subjects. The sponsor will be responsible for notifying the FDA of study termination, and the Investigator will be responsible for notifying the IRB.

13. REFERENCES

1. **Smith, J. M., R. R. Amara, D. Campbell, Y. Xu, M. Patel, S. Sharma, S. T. Butera, D. L. Ellenberger, H. Yi, L. Chennareddi, J. G. Herndon, L. S. Wyatt, D. Montefiori, B. Moss, H. M. McClure, and H. L. Robinson. 2004. DNA/MVA vaccine for HIV type 1: effects of codon-optimization and the expression of aggregates or virus-like particles on the immunogenicity of the DNA prime. *AIDS Res Hum Retroviruses* 20:1335-1347.**
2. **Wyatt, L. S., I. M. Belyakov, P. L. Earl, J. A. Berzofsky, and B. Moss. 2008. Enhanced cell surface expression, immunogenicity and genetic stability resulting from a spontaneous truncation of HIV Env expressed by a recombinant MVA. *Virology* 372:260-272.**
3. **Mayr, A., Hochstein-Mintzel, and H. Stickl. 1975. Abstammung, Eigenschaften und Verwendung des attenuierten Vaccinia-stammes MVA. *Infection* 3:6-14.**
4. **Blanchard, T. J., A. Alcami, P. Andrea, and G. L. Smith. 1998. Modified vaccinia virus Ankara undergoes limited replication in human cells and lacks several immunomodulatory proteins: implications for use as a human vaccine. *J Gen Virol* 79:1159-1167.**
5. **Antoine, G., F. Scheiflinger, F. Dorner, and F. G. Falkner. 1998. The complete genomic sequence of the modified vaccinia Ankara strain: comparison with other orthopoxviruses. *Virology* 244:365-396.**
6. **Stickl, H., V. Hochstein-Mintzel, A. Mayr, H. Huber, H. Schafer, and A. Holzner. 1974. MVA vaccination against smallpox: clinical trials of an attenuated live vaccinia virus strain (MVA). *Dtsch. Med. Wochenschr* 99:2386-2392.**
7. **Amara, R. R., F. Villinger, J. D. Altman, S. L. Lydy, S. P. O'Neil, S. I. Staprans, D. C. Montefiori, Y. Xu, J. G. Herndon, L. S. Wyatt, M. A. Candido, N. L. Kozyr, P. L. Earl, J. M. Smith, H. L. Ma, B. D. Grimm, M. L. Hulsey, J. Miller, H. M. McClure, J. M. McNicholl, B. Moss, and H. L. Robinson. 2001. Control of a mucosal challenge and prevention of AIDS by a multiprotein DNA/MVA vaccine. *Science* 292:69-74.**
8. **Amara, R. R., F. Villinger, S. Staprans, J. D. Altman, D. Montefiori, N. L. Kozyr, Y. Xu, L. Wyatt, P. L. Earl, J. G. Herndon, H. M. McClure, B. Moss, and H. L. Robinson. 2002. Different patterns of immune responses but similar control of a mucosal immunodeficiency virus challenge by MVA and DNA/MVA vaccines. *J Virol* 76:7625-7631.**
9. **Sadagopal, S., R. R. Amara, D. C. Montefiori, L. S. Wyatt, S. I. Staprans, N. L. Kozyr, H. M. McClure, B. Moss, and H. L. Robinson. 2005. Signature for Long-term vaccine-mediated control of a SHIV-98.6P challenge: Stable low breadth and low frequency T cell response capable of co-producing IFN-gamma and IL-2. *J Virol* 79:3243-3253.**
10. **Sadogopal, S. A., R.R., Kannanganat, S., Sharma, S., Chennareddi, L., and Robinson, H.L. 2008. Expansion and Exhaustion of T-cell Responses during Mutational Escape from Long-Term Viral Control in Two DNA/Modified Vaccinia virus Ankara-Vaccinated and Simian-Human Immunodeficiency Virus SHIV-89.6P-Challenged Macaques *J Virol* 82:4149-4153.**
11. **Lai, L., D. Vodros, P. A. Kozlowski, D. C. Montefiori, R. L. Wilson, V. L. Akerstrom, L. Chennareddi, T. Yu, S. Kannanganat, L. Ofielu, F. Villinger, L. S.**

Wyatt, B. Moss, R. R. Amara, and H. L. Robinson. 2007. GM-CSF DNA: an adjuvant for higher avidity IgG, rectal IgA, and increased protection against the acute phase of a SHIV-89.6P challenge by a DNA/MVA immunodeficiency virus vaccine. *Virology* 369:153-167.

12. **Zhao, J., L. Lai, R. R. Amara, D. C. Montefiori, F. Villinger, L. Chennareddi, L. S. Wyatt, B. Moss, and H. L. Robinson. 2009. Preclinical studies of human immunodeficiency virus/AIDS vaccines: inverse correlation between avidity of anti-Env antibodies and peak postchallenge viremia. *J Virol* 83:4102-4111.**
13. **Ellenberger, D., R. A. Otten, B. Li, M. Aidoo, I. V. Rodriguez, C. A. Sariol, M. Martinez, M. Monsour, L. Wyatt, M. G. Hudgens, E. Kraiselburd, B. Moss, H. Robinson, T. Folks, and S. Butera. 2006. HIV-1 DNA/MVA vaccination reduces the per exposure probability of infection during repeated mucosal SHIV challenges. *Virology* 352:216-225.**
14. **Amara, R. R., J. M. Smith, S. I. Staprans, D. C. Montefiori, F. Villinger, J. D. Altman, S. P. O'Neil, N. L. Kozyr, Y. Xu, L. S. Wyatt, P. L. Earl, J. G. Herndon, J. M. McNicholl, H. M. McClure, B. Moss, and H. L. Robinson. 2002. Critical role for Env as well as Gag-Pol in control of a simian-human immunodeficiency virus 89.6P challenge by a DNA prime/recombinant modified vaccinia virus Ankara vaccine. *J Virol* 76:6138-6146.**
15. **Tang, Y., F. Villinger, S. Staprans, R. R. Amara, J. M. Smith, J. G. Herndon, and H. L. Robinson. 2002. Slowly declining reservoir of mostly unintegrated viral DNA in SHIV-89.6P-challenged DNA/rMVA-vaccinated macaques. *J Virol* 76:10147-10154.**
16. **Buge, S. L., H. L. Ma, R. R. Amara, L. S. Wyatt, P. L. Earl, F. Villinger, D. C. Montefiori, S. I. Staprans, Y. Xu, E. Carter, S. P. O'Neil, J. G. Herndon, E. Hill, B. Moss, H. L. Robinson, and J. M. McNicholl. 2003. Gp120-alum boosting of a Gag-Pol-Env DNA/MVA AIDS vaccine: poorer control of a pathogenic viral challenge. *AIDS Res Hum Retroviruses* 19:891-900.**
17. **Kannanganat, S., P. Nigam, V. Velu, P. L. Earl, L. Lai, L. Chennareddi, B. Lawson, R. L. Wilson, D. C. Montefiori, P. A. Kozlowski, B. Moss, H. L. Robinson, and R. R. Amara. 2010. Preexisting Vaccinia Virus Immunity Decreases SIV-Specific Cellular Immunity but Does Not Diminish Humoral Immunity and Efficacy of a DNA/MVA Vaccine. *J Immunol* 185:7262-7273.**
18. **Shin, H., and E. J. Wherry. 2007. CD8 T cell dysfunction during chronic viral infection. *Curr Opin Immunol* 19:408-415.**
19. **O'Connor, D., T. Friedrich, A. Hughes, T. M. Allen, and D. Watkins. 2001. Understanding cytotoxic T-lymphocyte escape during simian immunodeficiency virus infection. *Immunol Rev* 183:115-126.**
20. **Burns, D. P., C. Collignon, and R. C. Desrosiers. 1993. Simian immunodeficiency virus mutants resistant to serum neutralization arise during persistent infection of rhesus monkeys. *J Virol* 67:4104-4113.**
21. **Veazey, R. S., M. DeMaria, L. V. Chalifoux, D. E. Shvetz, D. R. Pauley, H. L. Knight, M. Rosenzweig, R. P. Johnson, R. C. Desrosiers, and A. A. Lackner. 1998. Gastrointestinal tract as a major site of CD4+ T cell depletion and viral replication in SIV infection. *Science* 280:427-431.**
22. **Soong, L., J. C. Xu, I. S. Grewal, P. Kima, J. Sun, B. J. Longley, Jr., N. H. Ruddle, D. McMahon-Pratt, and R. A. Flavell. 1996. Disruption of CD40-CD40 ligand**

interactions results in an enhanced susceptibility to Leishmania amazonensis infection. *Immunity* 4:263-273.

23. **Campbell, K. A., P. J. Ovendale, M. K. Kennedy, W. C. Fanslow, S. G. Reed, and C. R. Maliszewski. 1996. CD40 ligand is required for protective cell-mediated immunity to Leishmania major. *Immunity* 4:283-289.**
24. **Gibson, S. J., J. M. Lindh, T. R. Riter, R. M. Gleason, L. M. Rogers, A. E. Fuller, J. L. Oesterich, K. B. Gorden, X. Qiu, S. W. McKane, R. J. Noelle, R. L. Miller, R. M. Kedl, P. Fitzgerald-Bocarsly, M. A. Tomai, and J. P. Vasilakos. 2002. Plasmacytoid dendritic cells produce cytokines and mature in response to the TLR7 agonists, imiquimod and resiquimod. *Cell Immunol* 218:74-86.**
25. **Goepfert, P. A., M. L. Elizaga, A. Sato, L. Qin, M. Cardinali, C. M. Hay, J. Hural, S. C. DeRosa, O. D. DeFawe, G. D. Tomaras, D. C. Montefiori, Y. Xu, L. Lai, S. A. Kalams, L. R. Baden, S. E. Frey, W. A. Blattner, L. S. Wyatt, B. Moss, R. H. L., and N. H. V. T. Network. 2011. Phase 1 safety and immunogenicity testing of DNA and recombinant modified vaccinia Ankara vaccines expressing HIV-1 virus-like particles *J Infect Dis* 203:610-619.**
26. **Seder, R. A., P. A. Darrah, and M. Roederer. 2008. T-cell quality in memory and protection: implications for vaccine design. *Nat Rev Immunol* 8:247-258.**
27. **Kannanganat, S., C. Ibegbu, L. Chennareddi, H. L. Robinson, and R. R. Amara. 2007. Multiple-cytokine-producing antiviral CD4 T cells are functionally superior to single-cytokine-producing cells. *J Virol* 81:8468-8476.**
28. **Betts, M. R., M. C. Nason, S. M. West, S. C. De Rosa, S. A. Migueles, J. Abraham, M. M. Lederman, J. M. Benito, P. A. Goepfert, M. Connors, M. Roederer, and R. A. Koup. 2006. HIV nonprogressors preferentially maintain highly functional HIV-specific CD8+ T cells. *Blood* 107:4781-4789.**
29. **Huber, M., and A. Trkola. 2007. Humoral immunity to HIV-1: neutralization and beyond. *J Intern Med* 262:5-25.**
30. **Kiepiela, P., K. Ngumbela, C. Thobakgale, D. Ramduth, I. Honeyborne, E. Moodley, S. Reddy, C. de Pierres, Z. Mncube, N. Mkhwanazi, K. Bishop, M. van der Stok, K. Nair, N. Khan, H. Crawford, R. Payne, A. Leslie, J. Prado, A. Prendergast, J. Frater, N. McCarthy, C. Brander, G. H. Learn, D. Nickle, C. Rousseau, H. Coovadia, J. I. Mullins, D. Heckerman, B. D. Walker, and P. Goulder. 2007. CD8+ T-cell responses to different HIV proteins have discordant associations with viral load. *Nat Med* 13:46-53.**
31. **Novitsky, V., P. Gilbert, T. Peter, M. F. McLane, S. Gaolekwe, N. Rybak, I. Thior, T. Ndung'u, R. Marlink, T. H. Lee, and M. Essex. 2003. Association between virus-specific T-cell responses and plasma viral load in human immunodeficiency virus type 1 subtype C infection. *J Virol* 77:882-890.**
32. **Riviere, Y., M. B. McChesney, F. Porrot, F. Tanneau-Salvadori, P. Sansonetti, O. Lopez, G. Pialoux, V. Feuillie, M. Mollereau, S. Chamaret, and et al. 1995. Gag-specific cytotoxic responses to HIV type 1 are associated with a decreased risk of progression to AIDS-related complex or AIDS. *AIDS Res Hum Retroviruses* 11:903-907.**
33. **Rolland, M., D. Heckerman, W. Deng, C. M. Rousseau, H. Coovadia, K. Bishop, P. J. Goulder, B. D. Walker, C. Brander, and J. I. Mullins. 2008. Broad and Gag-**

biased HIV-1 epitope repertoires are associated with lower viral loads. *PLoS One* 3:e1424.

34. Deeks, S. G., and B. D. Walker. 2007. Human immunodeficiency virus controllers: mechanisms of durable virus control in the absence of antiretroviral therapy. *Immunity* 27:406-416.

35. Koup, R. A., J. T. Safrit, Y. Cao, C. A. Andrews, G. McLeod, W. Borkowsky, C. Farthing, and D. D. Ho. 1994. Temporal association of cellular immune responses with the initial control of viremia in primary human immunodeficiency virus type 1 syndrome. *J Virol* 68:4650-4655.

36. Yu, X. G., M. M. Addo, E. S. Rosenberg, W. R. Rodriguez, P. K. Lee, C. A. Fitzpatrick, M. N. Johnston, D. Strick, P. J. Goulder, B. D. Walker, and M. Altfeld. 2002. Consistent patterns in the development and immunodominance of human immunodeficiency virus type 1 (HIV-1)-specific CD8+ T-cell responses following acute HIV-1 infection. *J Virol* 76:8690-8701.

37. Markowitz, M., M. Vesanen, K. Tenner-Racz, Y. Cao, J. M. Binley, A. Talal, A. Hurley, X. Jin, M. R. Chaudhry, M. Yaman, S. Frankel, M. Heath-Chiozzi, J. M. Leonard, J. P. Moore, P. Racz, D. F. Nixon, and D. D. Ho. 1999. The effect of commencing combination antiretroviral therapy soon after human immunodeficiency virus type 1 infection on viral replication and antiviral immune responses. *J Infect Dis* 179:527-537.

38. Oxenius, A., D. A. Price, P. J. Easterbrook, C. A. O'Callaghan, A. D. Kelleher, J. A. Whelan, G. Sontag, A. K. Sewell, and R. E. Phillips. 2000. Early highly active antiretroviral therapy for acute HIV-1 infection preserves immune function of CD8+ and CD4+ T lymphocytes. *Proc Natl Acad Sci USA* 97:3382-3387.

39. Ortiz, G. M., D. F. Nixon, A. Trkola, J. Binley, X. Jin, S. Bonhoeffer, P. J. Kuebler, S. M. Donahoe, M. A. Demoitie, W. M. Kakimoto, T. Ketas, B. Clas, J. J. Heymann, L. Zhang, Y. Cao, A. Hurley, J. P. Moore, D. D. Ho, and M. Markowitz. 1999. HIV-1-specific immune responses in subjects who temporarily contain virus replication after discontinuation of highly active antiretroviral therapy. *J Clin Invest* 104:R13-18.

40. Kaufmann, D. E., M. Lichterfeld, M. Altfeld, M. M. Addo, M. N. Johnston, P. K. Lee, B. S. Wagner, E. T. Kalife, D. Strick, E. S. Rosenberg, and B. D. Walker. 2004. Limited durability of viral control following treated acute HIV infection. *PLoS Med* 1:e36.

41. Oxenius, A., D. A. Price, H. F. Gunthard, S. J. Dawson, C. Fagard, L. Perrin, M. Fischer, R. Weber, M. Plana, F. Garcia, B. Hirschel, A. McLean, and R. E. Phillips. 2002. Stimulation of HIV-specific cellular immunity by structured treatment interruption fails to enhance viral control in chronic HIV infection. *Proc Natl Acad Sci USA* 99:13747-13752.

42. Goonetilleke, N., M. K. Liu, J. F. Salazar-Gonzalez, G. Ferrari, E. Giorgi, V. V. Ganusov, B. F. Keele, G. H. Learn, E. L. Turnbull, M. G. Salazar, K. J. Weinhold, S. Moore, N. Letvin, B. F. Haynes, M. S. Cohen, P. Hraber, T. Bhattacharya, P. Borrow, A. S. Perelson, B. H. Hahn, G. M. Shaw, B. T. Korber, and A. J. McMichael. 2009. The first T cell response to transmitted/founder virus contributes to the control of acute viremia in HIV-1 infection. *J Exp Med* 206:1253-1272.

43. Salazar-Gonzalez, J. F., M. G. Salazar, B. F. Keele, G. H. Learn, E. E. Giorgi, H. Li, J. M. Decker, S. Wang, J. Baalwa, M. H. Kraus, N. F. Parrish, K. S. Shaw, M. B. Guffey, K. J. Bar, K. L. Davis, C. Ochsenbauer-Jambor, J. C. Kappes, M. S. Saag, M. S. Cohen, J. Mulenga, C. A. Derdeyn, S. Allen, E. Hunter, M. Markowitz, P. Hraber, A. S. Perelson, T. Bhattacharya, B. F. Haynes, B. T. Korber, B. H. Hahn, and G. M. Shaw. 2009. Genetic identity, biological phenotype, and evolutionary pathways of transmitted/founder viruses in acute and early HIV-1 infection. *J Exp Med* 206:1273-1289.

44. El-Sadr, W. M., J. D. Lundgren, J. D. Neaton, F. Gordin, D. Abrams, R. C. Arduino, A. Babiker, W. Burman, N. Clumeck, C. J. Cohen, D. Cohn, D. Cooper, J. Darbyshire, S. Emery, G. Fatkenheuer, B. Gazzard, B. Grund, J. Hoy, K. Klingman, M. Losso, N. Markowitz, J. Neuhaus, A. Phillips, and C. Rappoport. 2006. CD4+ count-guided interruption of antiretroviral treatment. *N Engl J Med* 355:2283-2296.

45. Schooley, R. T., J. Spritzler, H. Wang, M. M. Lederman, D. Havlir, D. R. Kuritzkes, R. Pollard, C. Battaglia, M. Robertson, D. Mehrotra, D. Casimiro, K. Cox, B. Schock, and A. C. T. G. S. Team. AIDS clinical trials group 5197: a placebo-controlled trial of immunization of HIV-1-infected persons with a replication-deficient adenovirus type 5 vaccine expressing the HIV-1 core protein. *J Infect Dis* 202:705-716.

46. Hammer, S. M., J. J. Eron, Jr., P. Reiss, R. T. Schooley, M. A. Thompson, S. Walmsley, P. Cahn, M. A. Fischl, J. M. Gatell, M. S. Hirsch, D. M. Jacobsen, J. S. Montaner, D. D. Richman, P. G. Yeni, and P. A. Volberding. 2008. Antiretroviral treatment of adult HIV infection: 2008 recommendations of the International AIDS Society-USA panel. *JAMA* 300:555-570.

47. Ribaudo, H. J., D. W. Haas, C. Tierney, R. B. Kim, G. R. Wilkinson, R. M. Gulick, D. B. Clifford, C. Marzolini, C. V. Fletcher, K. T. Tashima, D. R. Kuritzkes, and E. P. Acosta. 2006. Pharmacogenetics of plasma efavirenz exposure after treatment discontinuation: an Adult AIDS Clinical Trials Group Study. *Clin Infect Dis* 42:401-407.

48. Interpretation and Use of the Western Blot Assay for Serodiagnosis of Human Immunodeficiency Virus Type 1 Infections MMWR 38(S-7);1-7; 07/21/1989

49. Fellay, J., K. Boubaker, B. Ledergerber, E. Bernasconi, H. Furrer, M. Battegay, B. Hirschel, P. Vernazza, P. Francioli, G. Greub, M. Flepp, and A. Telenti. 2001. Prevalence of adverse events associated with potent antiretroviral treatment: Swiss HIV Cohort Study. *Lancet* 358:1322-1327.

50. Benbrik, E., P. Chariot, S. Bonavaud, M. Ammi-Said, E. Frisdal, C. Rey, R. Gherardi, and G. Barlovatz-Meimon. 1997. Cellular and mitochondrial toxicity of zidovudine (AZT), didanosine (ddI) and zalcitabine (ddC) on cultured human muscle cells. *J Neurol Sci* 149:19-25.

51. Roubenoff, R., P. R. Skolnik, A. Shevitz, L. Snydman, A. Wang, S. Melanson, and S. Gorbach. 1999. Effect of a single bout of acute exercise on plasma human immunodeficiency virus RNA levels. *J Appl Physiol* 86:1197-1201.

52. Moss, B. 1996. Genetically engineered poxviruses for recombinant gene expression, vaccination, and safety. *Proc Natl Acad Sci USA* 93:11341-11348.

53. Moss, B., M. W. Carroll, L. S. Wyatt, J. R. Bennink, V. M. Hirsch, S. Goldstein, W. R. Elkins, T. R. Fuerst, J. D. Lifson, M. Piatak, N. P. Restifo, W. Overwijk, R.

Chamberlain, S. A. Rosenberg, and G. Sutter. 1996. Host range restricted, non-replicating vaccinia virus vectors as vaccine candidates. *Adv Exp Med Biol* 397:7-13.

54. Mayr, A., H. Stickl, H. K. Muller, K. Danner, and H. Singer. 1978. [The smallpox vaccination strain MVA: marker, genetic structure, experience gained with the parenteral vaccination and behavior in organisms with a debilitated defence mechanism (author's transl)]. *Zentralbl Bakteriol [B]* 167:375-390.

55. Harrer E, B. M., Ferstl B, et al. 2005. Therapeutic vaccination of HIV-1 infected patients on HAART with a recombinant HIV-1 nef-expressing MVA: safety, immunogenicity and influence on viral load during treatment interruption. *Antivir Ther* 10:285-300.

56. Vermont, C. L., H. H. van Dijken, C. J. van Limpt, R. de Groot, L. van Alphen, and G. P. van Den Dobbelsteen. 2002. Antibody avidity and immunoglobulin G isotype distribution following immunization with a monovalent meningococcal B outer membrane vesicle vaccine. *Infect Immun* 70:584-590.

57. Forthal, D. N., G. Landucci, and E. S. Daar. 2001. Antibody from patients with acute human immunodeficiency virus (HIV) infection inhibits primary strains of HIV type 1 in the presence of natural-killer effector cells. *J Virol* 75:6953-6961.
