# Supplementary material for: DNA/MVA Vaccination of HIV-1 Infected Participants with Viral Suppression on Antiretroviral Therapy, followed by Treatment Interruption: Elicitation of Immune Responses without Control of Re-Emergent Virus
Source: PLoS One. 2016 Oct 6;11(10):e0163164. doi: 10.1371/journal.pone.0163164 (PMC5053438; doi:10.1371/journal.pone.0163164)
Supplement: S1 Table — (DOCX) [file pone.0163164.s005.docx]

**S1 Table.** Clinical Adverse Events

| **Mild Events with Two or More Occurrences and All Moderate Events** | | | |
| --- | --- | --- | --- |
| **Preferred Term** | **Mild** | **Moderate** | **Total** |
| Headache | 5 | 2 | 7 |
| Upper Respiratory Tract Infection | 5 | 1 | 6 |
| Myalgia | 5 | 1 | 6 |
| Nasal Congestion | 6 | 0 | 6 |
| Oropharyngeal Pain | 5 | 1 | 6 |
| Cough | 4 | 1 | 5 |
| Diarrhea | 4 | 0 | 4 |
| Back Pain | 4 | 0 | 4 |
| Lymphadenopathy | 3 | 0 | 3 |
| Vomiting | 3 | 0 | 3 |
| Fatigue | 3 | 0 | 3 |
| Pyrexia | 2 | 1 | 3 |
| Vessel Puncture Site Haematoma | 3 | 0 | 3 |
| Throat Irritation | 3 | 0 | 3 |
| Contusion | 1 | 1 | 2 |
| Arthralgia | 2 | 0 | 2 |
| Sciatica | 1 | 1 | 2 |
| Insomnia | 2 | 0 | 2 |
| Productive Cough | 2 | 0 | 2 |
| Pulmonary Congestion | 2 | 0 | 2 |
| Night Sweats | 2 | 0 | 2 |
| Chest Discomfort | 0 | 1 | 1 |
| Gastroenteritis | 0 | 1 | 1 |
| Helicobacter Infection | 0 | 1 | 1 |
| Arthropod Bite | 0 | 1 | 1 |
| Depression | 0 | 1 | 1 |
| Upper-Airway Cough Syndrome | 0 | 1 | 1 |
