# Supplementary material for: DNA/MVA Vaccination of HIV-1 Infected Participants with Viral Suppression on Antiretroviral Therapy, followed by Treatment Interruption: Elicitation of Immune Responses without Control of Re-Emergent Virus
Source: PLoS One. 2016 Oct 6;11(10):e0163164. doi: 10.1371/journal.pone.0163164 (PMC5053438; doi:10.1371/journal.pone.0163164)
Supplement: S2 Table — (DOCX) [file pone.0163164.s006.docx]

**S2 Table.** Reactogenicity

| **Preferred Term** | **Mild** | **Mod** | **Sev** | **Ser** | **Total** |
| --- | --- | --- | --- | --- | --- |
| Diarrhea | 1 | 0 | 0 | 0 | 1 |
| Asthenia | 0 | 1 | 0 | 0 | 1 |
| Fatigue | 1 | 1 | 0 | 0 | 2 |
| Injection Site Erythema | 1 | 0 | 0 | 0 | 1 |
| Injection Site Edema | 1 | 0 | 0 | 0 | 1 |
| Injection Site Pain | 15 | 2 | 0 | 0 | 17 |
| Injection Site Warmth | 1 | 0 | 0 | 0 | 1 |
| Dysgeusia | 1 | 0 | 0 | 0 | 1 |
| Throat Irritation | 1 | 0 | 0 | 0 | 1 |
| Pruritus Generalised | 2 | 0 | 0 | 0 | 2 |
| **Total** | **24** | **4** | **0** | **0** | **28** |
