# Supplementary material for: DNA/MVA Vaccination of HIV-1 Infected Participants with Viral Suppression on Antiretroviral Therapy, followed by Treatment Interruption: Elicitation of Immune Responses without Control of Re-Emergent Virus
Source: PLoS One. 2016 Oct 6;11(10):e0163164. doi: 10.1371/journal.pone.0163164 (PMC5053438; doi:10.1371/journal.pone.0163164)
Supplement: S3 Table — (DOCX) [file pone.0163164.s007.docx]

**S3 Table.** Laboratory Adverse Events

| **Laboratory Value** | **Grade 1** | **Grade 2** | **Grade 3** | **Grade 4** |
| --- | --- | --- | --- | --- |
|  | **n** | **n** | **n** | **n** |
| Total Bilirubin | 13 | 8 | 5 | 0 |
| Carbon Dioxide | 22 | 0 | 0 | 0 |
| Blood Glucose | 13 | 1 | 0 | 0 |
| AST | 12 | 0 | 0 | 0 |
| Sodium | 11 | 0 | 0 | 0 |
| ALT | 10 | 0 | 0 | 0 |
| Potassium | 7 | 1 | 0 | 0 |
| Total Creatine Phosphokinase | 3 | 0 | 2 | 0 |
| Total Cholesterol | 3 | 1 | 0 | 0 |
| Calcium | 2 | 1 | 0 | 0 |
| Alkaline Phosphatase | 2 | 0 | 0 | 0 |
| LDL Cholesterol | 2 | 0 | 0 | 0 |
| Albumin | 0 | 0 | 1 | 0 |
| Creatinine | 1 | 0 | 0 | 0 |
| Absolute Neutrophils | 1 | 0 | 0 | 0 |
| **Total** | **102** | **12** | **8** | **0** |
